# A novel phosphocholine‐mimetic inhibits a pro‐inflammatory conformational change in C‐reactive protein

**DOI:** 10.15252/emmm.202216236

**Published:** 2022-12-05

**Authors:** Johannes Zeller, Karen S Cheung Tung Shing, Tracy L Nero, James D McFadyen, Guy Krippner, Balázs Bogner, Sheena Kreuzaler, Jurij Kiefer, Verena K Horner, David Braig, Habiba Danish, Sara Baratchi, Mark Fricke, Xiaowei Wang, Michel G Kather, Bernd Kammerer, Kevin J Woollard, Prerna Sharma, Craig J Morton, Geoffrey Pietersz, Michael W Parker, Karlheinz Peter, Steffen U Eisenhardt

**Affiliations:** ^1^ Department of Plastic and Hand Surgery, University of Freiburg Medical Centre Medical Faculty of the University of Freiburg Freiburg Germany; ^2^ Baker Heart and Diabetes Institute Melbourne Vic. Australia; ^3^ Department of Biochemistry and Pharmacology, Bio21 Molecular Science and Biotechnology Institute The University of Melbourne Parkville Vic. Australia; ^4^ Department of Cardiometabolic Health The University of Melbourne Parkville Vic. Australia; ^5^ ACRF Rational Drug Discovery Centre St. Vincent's Institute of Medical Research Fitzroy Vic. Australia; ^6^ School of Health and Biomedical Sciences RMIT University Melbourne Vic. Australia; ^7^ Centre for Integrative Signalling Analysis CISA University of Freiburg Freiburg Germany; ^8^ Centre for Inflammatory Disease Imperial College London London UK

**Keywords:** anti‐inflammatory therapy, C‐reactive protein, drug development, ischemia, x‐ray crystallography, reperfusion injury, Immunology, Microbiology, Virology & Host Pathogen Interaction, Pharmacology & Drug Discovery

## Abstract

C‐reactive protein (CRP) is an early‐stage acute phase protein and highly upregulated in response to inflammatory reactions. We recently identified a novel mechanism that leads to a conformational change from the native, functionally relatively inert, pentameric CRP (pCRP) structure to a pentameric CRP intermediate (pCRP*) and ultimately to the monomeric CRP (mCRP) form, both exhibiting highly pro‐inflammatory effects. This transition in the inflammatory profile of CRP is mediated by binding of pCRP to activated/damaged cell membranes via exposed phosphocholine lipid head groups. We designed a tool compound as a low molecular weight CRP inhibitor using the structure of phosphocholine as a template. X‐ray crystallography revealed specific binding to the phosphocholine binding pockets of pCRP. We provide *in vitro* and *in vivo* proof‐of‐concept data demonstrating that the low molecular weight tool compound inhibits CRP‐driven exacerbation of local inflammatory responses, while potentially preserving pathogen‐defense functions of CRP. The inhibition of the conformational change generating pro‐inflammatory CRP isoforms via phosphocholine‐mimicking compounds represents a promising, potentially broadly applicable anti‐inflammatory therapy.

## Graphical abstract

A novel low molecular weight compound C10M was designed to block the PC binding site on pCRP, thereby inhibiting the formation of the pro‐inflammatory isoforms pCRP*/mCRP, and thus showing broad anti‐inflammatory effects *in vitro* and *in vivo*.

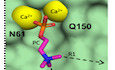

## Introduction

C‐reactive protein (CRP) is an evolutionarily highly conserved, acute phase protein that is synthesized in the liver under the regulation of the cytokine interleukin 6 (IL6) and circulates as a disc‐shaped homo‐pentamer (pCRP) (Zeller *et al*, 2022). While purified native human pCRP itself is described not to be pro‐inflammatory when injected into healthy individuals (Lane *et al*, 2014), it may cause a significant blood pressure drop when injected in rabbits (Bock *et al*, 2020), and it amplifies tissue injury in the context of inflammation and ischemia (McFadyen *et al*, 2018). There is strong evidence that administration of human pCRP can significantly increase tissue injury in animal models of myocardial infarction (Griselli *et al*, 1999; Pepys *et al*, 2006), stroke (Gill *et al*, 2004), and other organ ischemia/hypoxia and ischemia/reperfusion injuries (IRIs) (Padilla *et al*, 2007; McFadyen *et al*, 2018; Thiele *et al*, 2018). Furthermore, human CRP activates the rat complement system after dissociation to pCRP*/mCRP on the sites of inflamed tissue and thereby aggravates the ongoing inflammation, while rat CRP has been described not to activate autologous complement (de Beer *et al*, 1982; Griselli *et al*, 1999; Thiele *et al*, 2014; Braig *et al*, 2017). In line with the aggravating effects of human pCRP*/mCRP in animal models, recent clinical studies revealed a correlation between CRP concentration and cardiac infarct size (Ries *et al*, 2021), and clinical deterioration and harmful inflammatory responses in COVID‐19. Importantly, the therapeutic reduction of CRP levels by CRP‐apheresis was highly beneficial in these clinical settings (Ringel *et al*, 2021; Schumann *et al*, 2021; Torzewski *et al*, 2021; Esposito *et al*, 2022).

Previously, we identified a novel mechanism that can be viewed as a pro‐inflammatory “CRP‐activation” process (Eisenhardt *et al*, 2009a; Braig *et al*, 2014). In the context of inflammation and tissue damage, membrane changes on activated cell membranes mediated by phospholipase A2 lead to the exposure of bioactive lipids (Nijmeijer *et al*, 2002, 2003; Thiele *et al*, 2014). This results in the binding of circulating pCRP and subsequent changes in CRP conformation from the pentameric structure (pCRP) to a partially dissociated pentamer (pCRP*) and ultimately in dissociation to its monomeric form (mCRP) (Eisenhardt *et al*, 2009a; Molins *et al*, 2011; Braig *et al*, 2017). pCRP* and mCRP are strong pro‐inflammatory agents, and can induce IL‐8 secretion in neutrophils (Khreiss *et al*, 2005) and human coronary artery endothelial cells (Khreiss *et al*, 2004), promote neutrophil–endothelial cell adhesion (Zouki *et al*, 2001), and delay apoptosis of human neutrophils (Khreiss *et al*, 2002). pCRP* can also bind and activate complement C1q (Braig *et al*, 2017), which further contributes to aggravation of pre‐existing inflammation and detrimental tissue damage (Eisenhardt *et al*, 2009a; Thiele *et al*, 2014). Based on this central role of CRP in inflammatory reactions and diseases, it represents an attractive therapeutic target.

Phosphocholine (PC), phosphoserine (PS), and phosphoethanolamine (PE) head groups of bioactive lipids are exposed on the surface of activated and damaged cells. The PC, PS, and PE head groups are known to bind to a shallow groove containing two calcium cations located on one face of the monomeric subunit of pCRP; hence, there are five PC/PE binding pockets on the same face of the CRP pentamer (i.e., the B‐face). The concept of targeting the PC/PE binding pockets of pCRP with a synthetic ligand was first explored by Pepys *et al* (2006), when they synthesized and evaluated palindromic (or bivalent) compounds comprising two molecules of PC covalently linked through one of the phosphate oxygen atoms by a flexible carbon‐based linker; one such compound was 1,6‐bis(phosphocholine)‐hexane (bis‐PC, mw ~ 450 Da). It was demonstrated by X‐ray crystallography that each of the PC head groups of bis‐PC binds to two separate CRP pentamers, and when multiple bis‐PC molecules bind they bring the PC‐binding surfaces (i.e., the B‐face) of the two pentamers together in a parallel fashion. By binding in this manner, bivalent compounds like bis‐PC prevent pCRP from interacting with bioactive lipids on activated/damaged cell surfaces, thereby blocking the formation of pCRP* and mCRP. In addition to bivalent compounds like bis‐PC, other CRP‐targeting therapeutic strategies have been explored (Zeller *et al*, 2022). Helical polypeptides with covalently attached (via a carbon‐based linker) PC molecules (Tegler *et al*, 2011), peptide mimetics (mw > 700 Da) (Kumaresan *et al*, 2013), and anti‐sense oligonucleotides (ASO) (Warren & Flaim, n.d.) targeting CRP have been developed, with the latter reaching phase II clinical trials (ISIS‐329993 and ISIS‐353512, NCT01710852, NCT01414101, and NCT00734240; Warren *et al*, 2015). Furthermore, reducing circulating pCRP levels via CRP apheresis using PC‐linked resins is currently being investigated with promising initial results as an adjunct therapy to minimize cardiac injury in patients with myocardial infarction (Ries *et al*, 2019, 2021) and COVID‐19 (Pepys, 2021; Ringel *et al*, 2021; Schumann *et al*, 2021; Torzewski *et al*, 2021; Esposito *et al*, 2022). The therapeutic approaches discussed above target general pCRP inhibition and reduction of circulating pCRP levels. However, one theoretical concern of CRP reducing therapies is the potential risk of immunosuppression, especially in bacterial infections and sepsis, given CRP's well‐described role in the innate immune response. Intriguingly, in the ISIS studies, evaluating the reduction of endotoxin‐induced increase in CRP levels by pre‐treatment with ASO, healthy endotoxin‐challenged volunteers did not demonstrate any evidence of a compromised immune response and the ASO was well tolerated with no serious adverse events (Noveck *et al*, 2014). Moreover, in the phase II clinical trial, patients treated with a CRP ASO demonstrated no increase in infections compared to control patients (Warren *et al*, 2015). Other CRP lowering approaches, such as apheresis, have also shown that the therapeutic targeting of CRP does not appear to increase the risk of infection (Torzewski *et al*, 2020, 2021, 2022; Ries *et al*, 2021; Ringel *et al*, 2021; Schumann *et al*, 2021). Therefore, therapeutic targeting of CRP is an attractive anti‐inflammatory therapy approach and different strategies have been developed and are being tested with promising initial results.

We set out to design a low molecular weight monovalent tool compound that targets the PC/PE binding pocket on pCRP and thereby has the potential to prevent the formation of the pro‐inflammatory pCRP* and mCRP species. Our strategy follows a distinctly different design compared to the previously published bivalent approach, as it allows the CRP pentamer to remain intact and in circulation, with both the A‐ and B‐faces available to interact with ligands and retain associated functions. As proof‐of‐concept we present the data for a tool compound C10M, (3‐(dibutylamino)propyl)phosphonic acid, a monovalent CRP inhibitor with a molecular weight of just 250 Da. After confirming that C10M bound to the PC/PE binding pocket of pCRP using X‐ray crystallography and competitive affinity chromatography, we investigated the therapeutic potential of this compound in various *in vitro*, *ex vivo*, and *in vivo* experimental models of inflammation. We analyzed its mode of action and demonstrated that C10M prevents pCRP from binding to activated or damaged cell membranes, thereby blocking the formation of the pro‐inflammatory pCRP* and mCRP species. Furthermore, we tested the immunosuppressive impact of C10M on the CRP‐dependent innate immune response to bacterial pathogens to preserve protective capacities against *Streptococcus pneumoniae* (*S. pneumoniae*), *Escherichia coli* (*E. coli*), and yeast cell wall ligand zymosan (*Saccharomyces cerevisiae*) to present a targeted therapy against exacerbated inflammation. Overall, we introduced and characterized a novel anti‐inflammatory approach targeting the interaction of PC with pCRP, preventing the generation of pro‐inflammatory pCRP*/mCRP.

## Results

### Design of a tool compound, CRP inhibitor C10M

**Figure 1:**
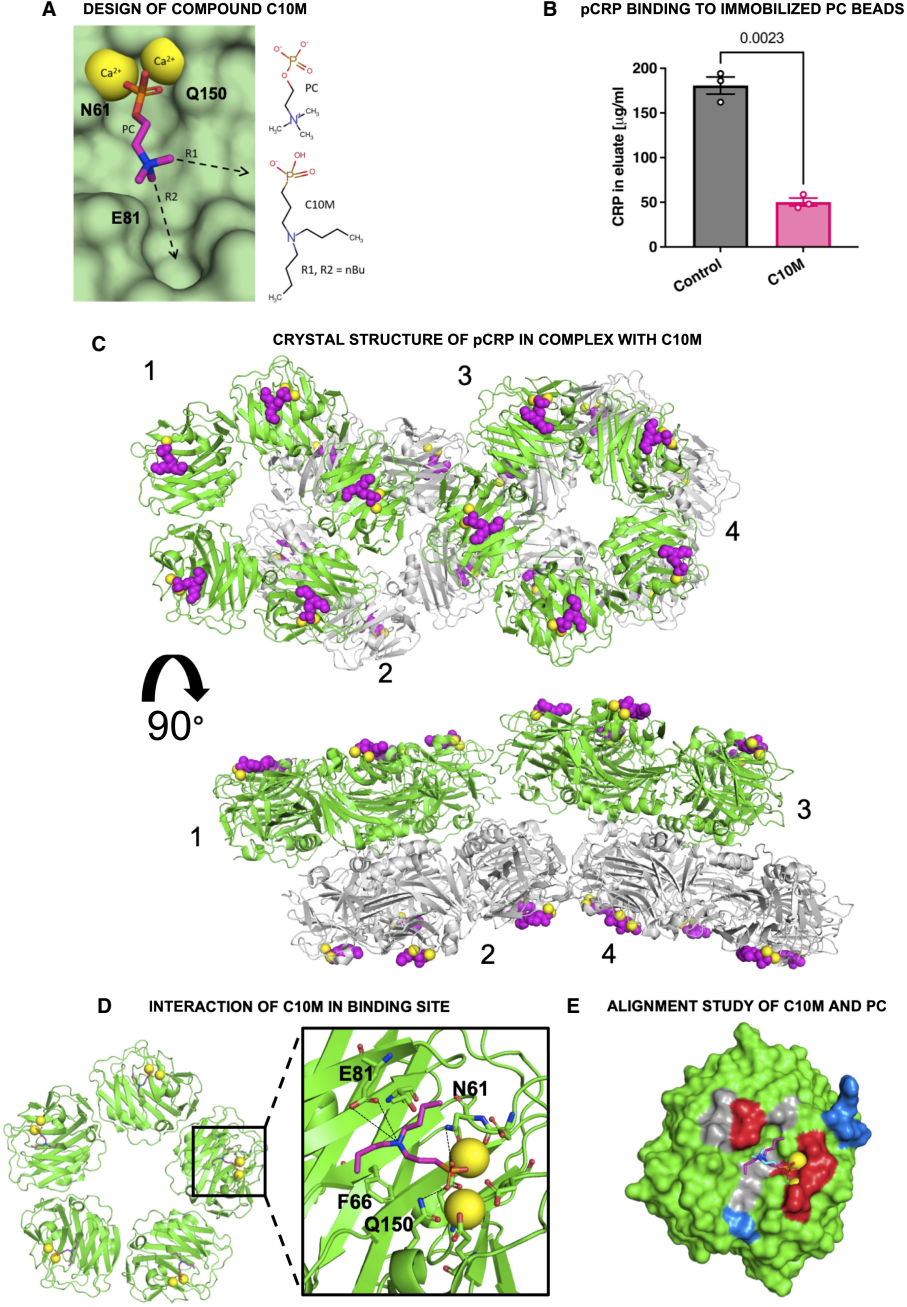
Binding studies of tool compound C10M to pCRP *in vitro* Design of the phosphonate compound C10M was guided by the PC:pCRP complex (PDB ID: 1B09), with two n‐butyl substituents (nBu) on the tertiary amine exploiting available space in the binding pocket along the denoted vectors R1 and R2. Calcium cations shown as yellow spheres and the location of pCRP residues Asn 61 (N61), Glu 81 (E81), and Gln 150 (Q150) are indicated. pCRP depicted as a light green molecular surface.C10M reduces binding of pCRP to immobilized PC. pCRP was incubated with p‐aminophenyl phosphoryl choline agarose beads under binding conditions with and without C10M. Porous solid column chromatography was then used to evaluate the binding capacity of pCRP to PC. Biological replicates, *n* = 3, mean ± SEM. *P* value was calculated with Student's *t*‐test.The crystal structure of pCRP (shown as cartoon) in complex with C10M (pink spheres) confirmed that the compound binds to the same pocket as PC/PE. The asymmetric unit consisted of four stacked pCRP pentamers; two pairs of pentamers, 1 & 2 and 3 & 4, are stacked A‐face to A‐face. Pentamers 2 and 4 are colored gray to show their relative location to pentamers 1 and 3 (colored green). C10M was bound to all CRP monomeric subunits (Fig EV1; Tables EV1 and EV2). Each CRP monomeric subunit contained two calcium cations (depicted as yellow spheres). Orthogonal views of the asymmetric unit are shown.Structure of one pentamer from the asymmetric unit. Interaction of the phosphonate moiety with the bound calcium cations (yellow spheres) and hydrogen bonds with Asn 61 (N61), Glu 81 (E81), and Gln 150 (Q150) (black dashed lines) anchor the compound in the binding pocket. Full list of interacting residues is given in Table EV1.Alignment of C10M (pink/blue/red/orange sticks) and PC (cyan/blue/red/orange sticks) in complex with pCRP via the Cα atoms of pCRP. One monomeric subunit of pCRP is depicted as a molecular surface, the location of acidic (red), basic (blue) and hydrophobic (gray) residues around the PC binding pocket is indicated. Calcium cations shown as yellow spheres. Source data are available online for this figure.

To design a proof‐of‐concept tool compound, a monovalent CRP inhibitor, we targeted the interactions between the PC/PE head groups of bioactive lipids with pCRP. The PC/PE binding pockets are located on what is designated as the B‐face of the disc‐shaped CRP pentamer (Braig *et al*, 2017). Once bound, the lipid PC/PE head groups anchor the pCRP protein to the damaged cell surface, which then initiates the conformational change to pCRP* and ultimately mCRP (Braig *et al*, 2017). The face of the CRP pentamer not containing the PC/PE binding pockets is known as the A‐face and is not involved in binding to activated/damaged cell membranes. PC is anchored to the shallow groove on the B‐face of pCRP monomers via (i) a salt bridge interaction between the positively charged quaternary amine and the negatively charged carboxyl group of Glu 81 and (ii) coordination of the negatively charged phosphate moiety to the positively charged calcium cations (Fig 1A). In addition, pCRP residues Asn 61 and Gln 150 lie on opposite sides of the binding pocket and their side chains participate in hydrogen bonds with oxygen atoms of the PC phosphate moiety, while the other residues lining the binding pocket contribute hydrophobic interactions only (the full list of interacting pCRP residues is given in Table EV1). The tool compound C10M (Fig 1A) was intended to mimic the anchoring interactions of PC. To retain the critical coordination to the calcium cations, while removing susceptibility to serum nuclease activities, the PC phosphate moiety was replaced with a phosphonate. To take advantage of the space in the binding pocket near the PC quaternary amine (indicated by the vectors R1 and R2, Fig 1A), we replaced the methyl substituents of PC with n‐butyl groups and reverted to a tertiary amine to mitigate any steric hindrance to accessing Glu 81 that the longer n‐butyl groups may cause. C10M was synthesized by a two‐step synthetic method from commercially available precursors in reasonable yield.

### C10M binds to and inhibits pCRP *in vitro*

The inhibitory effect of the low molecular weight tool compound C10M was initially investigated using solid column chromatography, with PC immobilized on agarose beads. C10M (1:100 pCRP:C10M molar ratio) reduced binding of pCRP (200 μg) to these beads by ~ 70% (Fig 1B).

To determine the mode of binding of compound C10M to pCRP, co‐crystallization experiments were undertaken and the structure of the complex was solved using X‐ray crystallography at a resolution of 3.5 Å (see Table EV2 for statistics and Fig 1C and D, and EV1A and B). The asymmetric unit consisted of four CRP pentamers with the A‐faces stacked against each other (Fig 1C). Each pCRP monomeric unit adopted a similar fold to that previously published for the PC:pCRP complex (PDB ID: 1B09; Thompson *et al*, 1999), with a root mean square deviation of 0.5 Å over all Cα atoms upon alignment. All of the pCRP monomers within the asymmetric unit had a C10M molecule bound. The location of the C10M molecules confirmed that the tool compound binds to the PC/PE binding pocket, with the phosphonate moiety being the main mediator of the interaction with pCRP via the calcium cations (Fig 1C through E). Residues Asn 61 and Gln 150 are in hydrogen bonding distance of oxygen atoms of the C10M phosphonate moiety, as observed in the PC:pCRP complex (Fig 1A and D; Table EV1). In contrast to the positively charged quaternary amine of PC, which interacts with Glu 81 via a salt bridge, the tertiary amine of C10M appears to form hydrogen bonds with Glu 81 (Fig 1D and E; Table EV1). The majority of interactions made by C10M with surrounding residues are hydrophobic or non‐polar. The n‐butyl amine substituents of C10M make numerous hydrophobic contacts with pCRP residues (a full list of putative interacting pCRP residues is given in Table EV1).

**Figure EV1:**
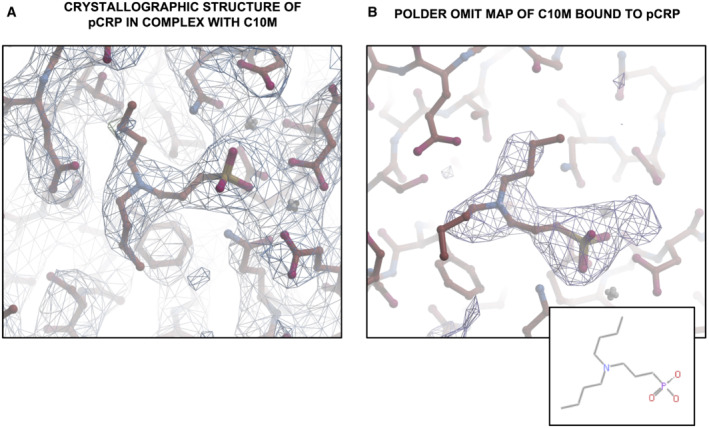
Crystallographic structure of pCRP in complex with tool compound C10M Crystallographic structure of pCRP in complex with C10M. 2*F*o‐*F*c electron density map of C10M bound to pCRP (contoured at 1σ).Polder omit map (Liebschner *et al*, 2017) of C10M bound to pCRP (contoured at 3σ). The 2D structure of C10M is shown in the inset at the bottom right.

### C10M inhibits pCRP binding to activated cell membranes

**Figure 2:**
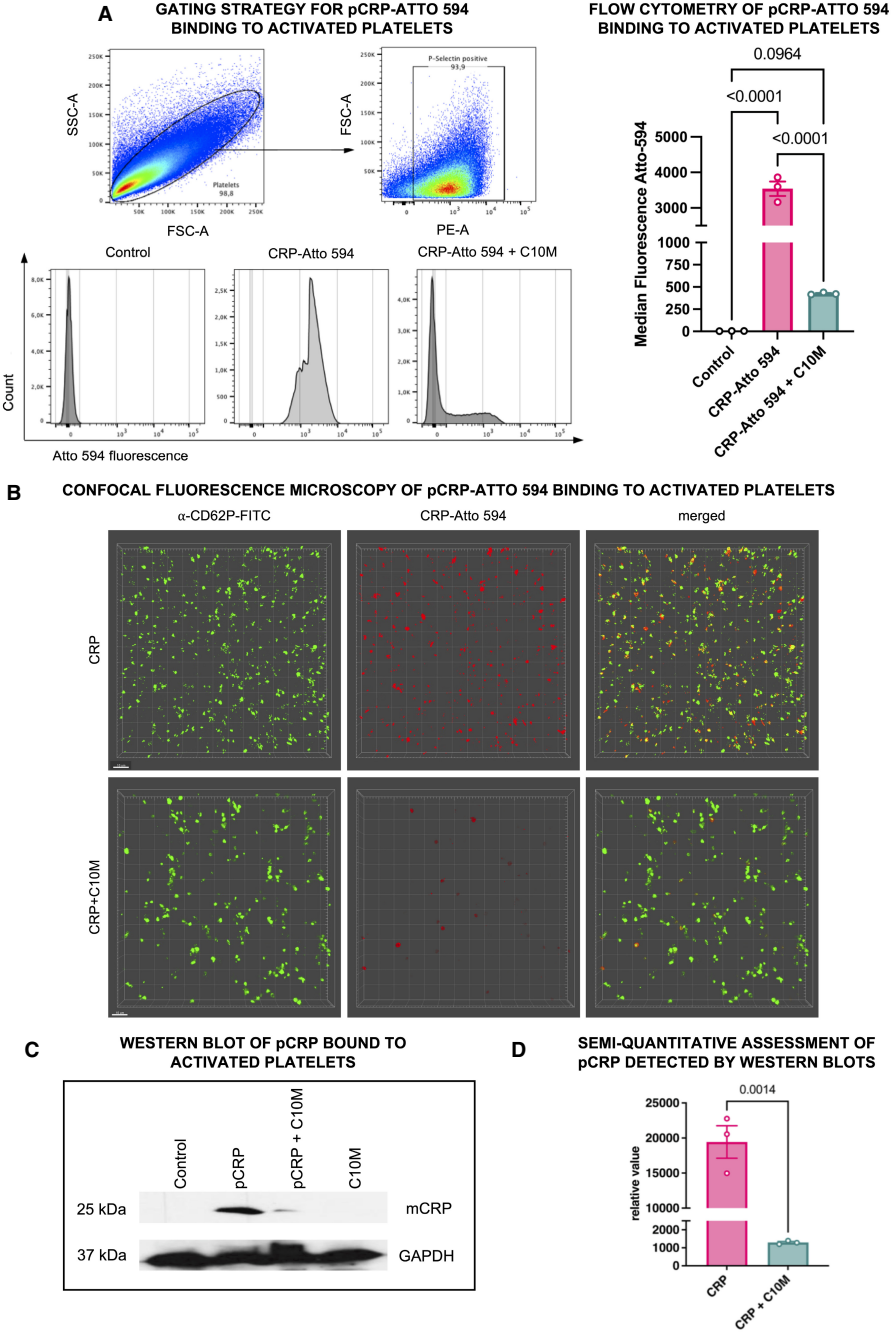
Studies on pCRP binding to activated platelets in the presence of C10M Flow cytometry was used to determine binding of pCRP to activated platelets. P‐selectin‐positive platelets were analyzed for CRP‐Atto 594 fluorescence. Platelets were incubated without labeled pCRP (control), and with labeled pCRP (50 μg/ml) ± C10M (1:100 molar ratio). Results are displayed as mean ± SEM. *P* values were calculated with ANOVA and Tukey's *post hoc* test. Biological replicate, *n* = 3.Confocal fluorescence microscopy of ADP‐activated human platelets in the presence of pCRP. Depicted are 3D reconstructions of monolayers of ADP (20 μM) activated platelets. Washed platelets were detected by anti‐CD62P (green). 50 μg/ml pCRP‐Atto 594 (red) was incubated with isolated platelets with (bottom) or without (top) C10M. pCRP localizes on platelets, which can be inhibited by C10M. Scale bar 10 μm.Western blot of pCRP binding to activated platelets. Platelets bind less pCRP when incubated with C10M as detected with the anti‐CRP antibody, compared to the anti‐GAPDH antibody as a control. Washed human platelets were incubated with pCRP and pCRP+C10M, respectively, lysed and separated on SDS‐PAGE.Densitometric quantification of protein bands of Western blots (biological replicates, *n* = 3) from the experiment described in (C). *P* value was calculated by Student's *t*‐test. Graph shows mean ± SEM. Source data are available online for this figure.

Activated platelet membranes expose the PC head groups of lysophosphatidylcholine (LPC), thereby mediating the conformational changes of CRP (Eisenhardt *et al*, 2009a, 2009b; Molins *et al*, 2011; Diehl *et al*, 2019). We have previously demonstrated that pCRP binds to activated cell membranes but not to membranes of healthy cells (Braig *et al*, 2017). After 120 min bound to activated cell membranes, pCRP begins to dissociate to the pro‐inflammatory pCRP* species; however, the proportion of pCRP* is greatest on the surface of microvesicles released by damaged cells. To investigate whether tool compound C10M binding to pCRP is sufficient to inhibit the pro‐inflammatory effects of CRP, we determined the ability of C10M to prevent pCRP binding to activated platelets. Binding of fluorescently labeled pCRP on isolated and ADP‐stimulated platelets was investigated by flow cytometry (Fig 2A). When incubated with C10M, significantly less CRP bound to activated platelets. Controls showed calcium‐dependent binding of CRP to platelets and no binding to isolated platelets in the absence of calcium: washed platelets in EDTA buffer showed no CRP binding, while washed platelets reconstituted with calcium showed CRP binding (shown in Appendix S1). Flow cytometric results were visualized and confirmed by confocal laser scanning microscopy (Fig 2B). This was further confirmed by Western blotting of activated platelets that were incubated with either pCRP alone or pCRP + C10M. After washing steps, only a small fraction of CRP could be detected by Western blot in the presence of C10M, whereas significant amounts of CRP were detectable in the platelet lysates without C10M. Data were quantified by densitometry (Fig 2C and D).

### C10M inhibits pCRP*/mCRP‐induced monocyte adhesion and pro‐inflammatory cytokine production

**Figure 3:**
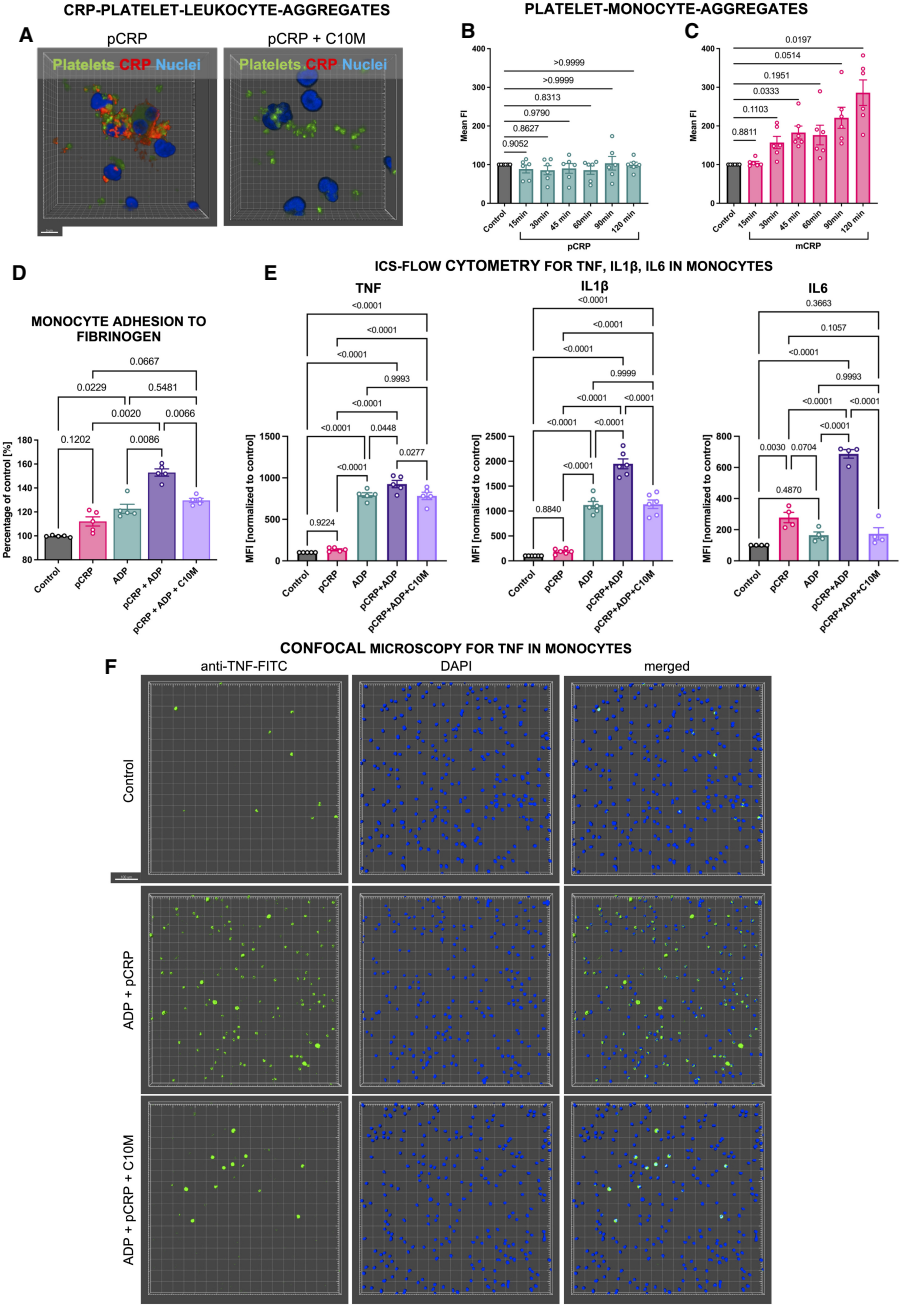
Studies on CRP‐dependent expression of pro‐inflammatory cytokines and inhibiting effects of C10M ACRP‐coated platelets visualized by confocal microscopy bound to whole blood leukocytes. Washed platelets (green) were incubated in serum supplemented with 50 μg/ml pCRP‐Atto 594 (red), without (left) and with C10M (right, 1:100 molar ratio). Platelets were then added to whole blood samples and imaged after RBC lysis. Shown are platelet–leukocyte aggregates with and without CRP attached. Scale bar indicates 5 μm.B, CCRP induces platelet–leukocyte aggregation in a time‐dependent and conformation‐specific manner. Whole blood samples stimulated with either 50 μg/ml pCRP (B) or mCRP (C) were analyzed by flow cytometry. Double positive events (CD62P and CD14) were identified as platelet–monocyte complexes. Incubation was stopped at indicated time points. Displayed are mean ± SEM. *P* values were calculated with ANOVA and Tukey's *post hoc* test. Biological replicates, *n* = 6.DStatic adhesion of monocytes to 20 μg/ml fibrinogen was tested as described before. Purified monocytes (3 × 10^6^ per ml) were stimulated with 50 μg/ml pCRP and 20 μM ADP and allowed to adhere for 35 min. ADP was used to activate supplemented platelets. Monocyte adhesion was quantified by calorimetric method and absorbance measured after 30 min. Results are shown as mean ± SEM. *P* values were calculated using ANOVA and Tukey's *post hoc* test. Biological replicates, *n* = 5.EExpression of TNF, IL1β and IL6 (from left to right) in monocytes analyzed by flow cytometry. The addition of C10M to the whole blood samples inhibits the CRP‐dependent expression of pro‐inflammatory cytokines. *P* values were calculated with ANOVA and Tukey's *post‐hoc* test. Biological replicates, *n* = 6 (IL6), *n* = 5 (TNF) and *n* = 4 (IL1β), respectively, bars indicate mean ± SEM.FRepresentative examples of whole blood samples treated with pCRP and C10M visualized by confocal fluorescence microscopy. Depicted are 3D reconstructions multiple focal planes at 20× magnification. The leukocytes were stained with DAPI (blue) and anti‐TNF‐FITC (green). Scale bar 50 μm. Source data are available online for this figure.

pCRP* and mCRP have previously been demonstrated to enhance leukocyte adhesion, transmigration, and subsequent cytokine production, all of which are key events in the inflammatory cascade. Therefore, we next investigated the inhibitory effects of C10M in abrogating these pro‐inflammatory properties of CRP. The potential of tool compound C10M to inhibit CRP‐induced monocyte adhesion, expression of pro‐inflammatory cytokines, and formation of platelet–leukocyte aggregates was evaluated *in vitro*. pCRP*/mCRP was generated by incubation of ADP‐stimulated platelets with pCRP as described previously (Eisenhardt *et al*, 2009a; Braig *et al*, 2017). The binding of pCRP on activated platelets in platelet–leukocyte aggregates was inhibited by C10M. Furthermore, mCRP itself leads to the formation of platelet–leukocyte aggregates, while pCRP shows no increased formation of aggregation (Fig 3A through C). In a static monocyte adhesion assay analyzing binding of monocytes to a fibrinogen matrix, pCRP* induces monocyte adhesion, which can be inhibited by C10M (Fig 3D). Notably, C10M is only reducing the pCRP*‐induced exacerbation of inflammation, which is represented by the increase in monocyte adhesion, but not the underlying increase in adhesion of monocytes that is induced by the ADP‐activated platelets.

We further analyzed the interaction of platelet‐bound pCRP*/mCRP with monocytes by intracellular staining (ICS) and flow cytometry. Cytokine expression levels of pro‐inflammatory cytokines, as measured by ICS, were found upregulated in monocytes (Fig 3E). Tumor necrosis factor (TNF), IL6 and IL1β were expressed at a low level when whole blood of healthy donors was incubated for 6 h or longer without stimulating agent (control). When incubated with pCRP, expression levels did not differ significantly from control. In contrast, ADP‐stimulated platelets caused increased expression of all three cytokines in monocytes, which, importantly, was significantly increased further by addition of pCRP. These exacerbating effects were blunted by C10M. To confirm the flow cytometric data, we performed confocal fluorescence microscopy (Fig 3F) and found TNF expression upregulated in cells incubated with ADP‐activated platelets and pCRP, resulting in pCRP* formation (middle row). In contrast, cells incubated with activated platelets, pCRP and C10M were not expressing more TNF than the control group (bottom row) due to the inhibition of pCRP* formation (Fig 3F).

### C10M inhibits pCRP binding to activated platelets and microvesicles and reduces pCRP*/mCRP induced ICAM‐1 and VCAM‐1 expression on human endothelial cells and activation of leukocytes

**Figure 4:**
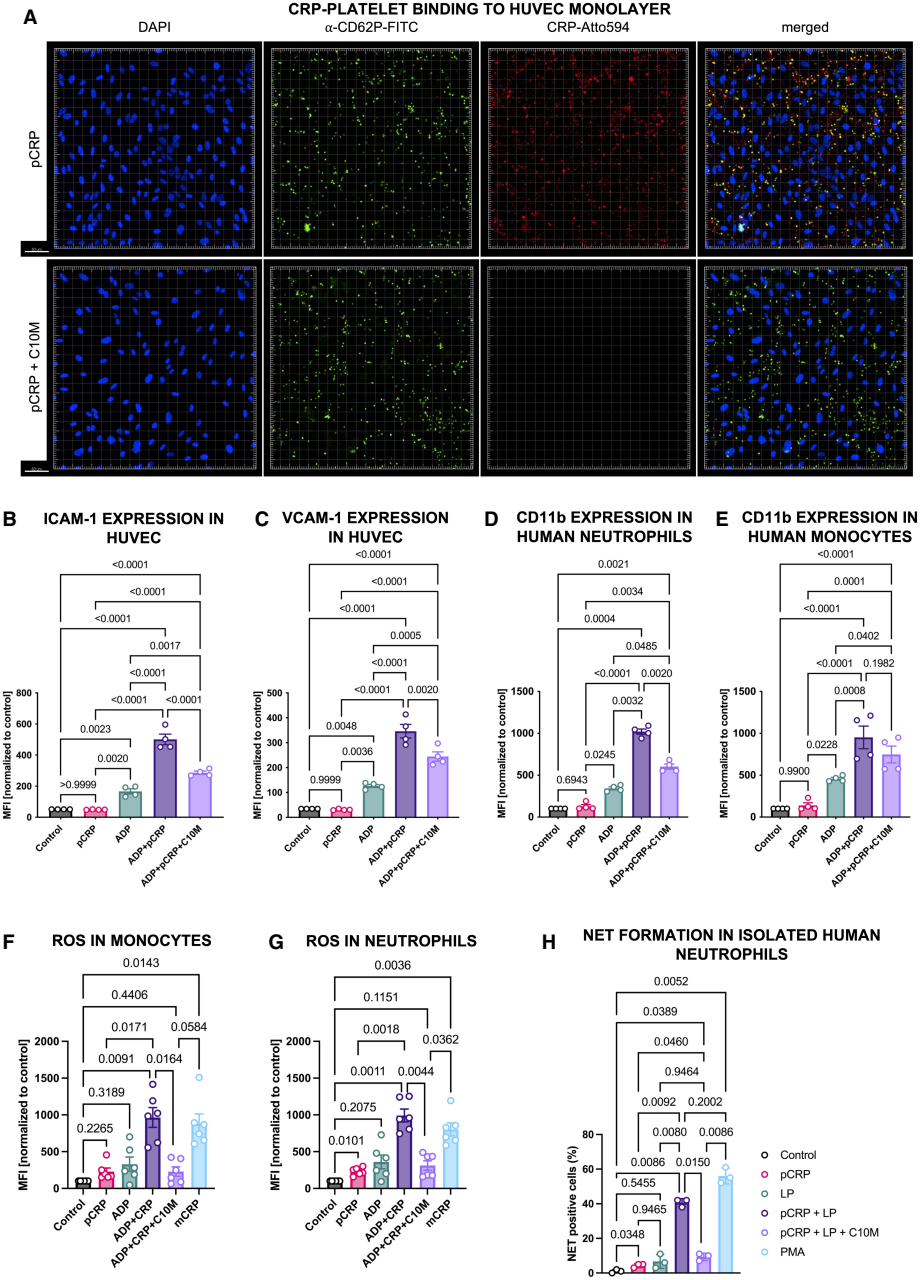
pCRP binding to ADP‐activated platelets is inhibited by C10M, reducing expression of adhesion molecules involved in leukocyte diapedesis in endothelial cells and leukocytes, ROS formation, and NET formation AConfocal fluorescence microscopy of ADP‐activated platelets bound to HUVEC mono cell layers. pCRP‐Atto 594 (depicted in red) was added and incubated with and without C10M. Anti‐CD62P‐FITC antibody was used to detect the platelets (green). HUVEC nuclei were counterstained with DAPI (blue). pCRP colocalizes with platelets on the endothelial cells. C10M inhibits CRP binding to activated platelets. Scale bar 50 μm.B, CQuantification of ICAM‐1 (B) and VCAM‐1 (C) expression on pCRP*/mCRP‐activated HUVECs. ICAM‐1 and VCAM‐1 expressions were measured by flow cytometry. ADP‐stimulated platelets were added to each sample (except for “Control” and “pCRP”) and served as activated cell membranes for pCRP dissociation to pCRP*/mCRP. C10M inhibits the generation of pCRP*/mCRP, thereby reducing the expression of ICAM‐1 and VCAM‐1. Mean fluorescence intensity (MFI) results in flow cytometry are shown with results normalized to control, mean ± SEM. *P* values were calculated with ANOVA and Tukey's *post‐hoc* test. Biological replicates, *n* = 4.D, EExpression of integrin subunit αM (CD11b) in neutrophils (D) and CD14^+^ monocytes (E) was accessed by flow cytometry as described previously (Kiefer *et al*, 2021). Human whole blood was incubated with 25 μg/ml pCRP, 20 μM ADP, and C10M (molar ratio 1:100, pCRP:C10M), respectively. CD11b expression was analyzed by flow cytometry in neutrophils (CD16^+^, SSC high) and monocytes (CD14^+^, SSC low). Shown are scatter plots of MFI results in flow cytometry with results normalized to control, mean ± SEM. *P* values were calculated with ANOVA and Tukey's *post‐hoc* test. Biological replicates, *n* = 4.F, GROS generation in whole blood detected in CD14^+^ monocytes (F) and neutrophils (G) by redox‐indicator dihydroethidium (DHE; 10 μg/ml). Blood samples incubated for 3 h at 37°C, 5% CO_2_ with 50 μg/ml pCRP and mCRP, 20 μM ADP and C10M (molar ratio 1:100, pCRP:C10M), respectively. Control was left unstimulated and mCRP served as a positive control (Thiele *et al*, 2018). Cells were washed after red blood cell lysis and analyzed by flow cytometry. Shown are MFI results with results normalized to control, mean ± SEM. *P* values were calculated with ANOVA and Tukey's *post‐hoc* test. Biological replicates, *n* = 6.HpCRP*/mCRP dependent NETosis in isolated human neutrophils detected by confocal immunofluorescence microscopy. Isolated neutrophils incubated for 3 h at 37°C, 5% CO_2_ with 100 μg/ml pCRP with and without PC:LPC liposomes (LP) and C10M (molar ratio 1:100, pCRP:C10M), respectively. Control was left unstimulated and 100 nM phorbol 12‐myristate 13‐acetate (PMA) served as a positive control. Cells were washed, fixed, and stained, and analyzed by confocal microscopy. Results are given as a ratio of NETing cells/all cells per ROI, with mean ± SEM. *P* values were calculated with ANOVA and Tukey's *post‐hoc* test. Biological replicates, *n* = 3. Source data are available online for this figure.

The interaction of immune cells with activated endothelial cells, leading to cell adhesion and transmigration, is a crucial event in localized tissue inflammation. We investigated the effects of pCRP* on endothelial cells and leukocytes to test the therapeutic anti‐inflammatory potential of C10M. Circulating pCRP binds to activated cells and is shed on microvesicles largely as pCRP*; this mechanism is crucial in transporting and mediating pCRP* in circulation *in vivo* (Habersberger *et al*, 2012; Braig *et al*, 2017). We therefore examined the effects of C10M on pCRP binding to human umbilical vein endothelial cell (HUVEC) monolayers and binding of ADP‐activated platelets and microvesicles derived from mononuclear cell lines (THP‐1) after LPS stimulation. pCRP on platelets and microvesicles, respectively, bind to HUVEC monolayers (Fig 4A, first row and Appendix S2). C10M significantly reduces this initial step of pCRP*‐mediated aggravation of inflammation (Fig 4A, second row and Appendix S2). To test the functional relevance of this process, we investigated the expression of ICAM‐1 and VCAM‐1 in HUVECs. C10M significantly reduces ICAM‐1 and VCAM‐1 expression induced by pCRP* on platelets and microvesicles, respectively (Fig 4B and C; Appendix S2). Upregulation of adhesion receptors represents the inflammatory response of the endothelial cells in the initial phase of inflammation (Rothlein *et al*, 1986; Yang *et al*, 2005). ICAM‐1 and VCAM‐1 are crucial endothelial ligands for receptors of the integrin family, essential for the adhesion and tissue infiltration of leukocytes. To look at this mechanism further, we examined the effects of pCRP* on neutrophil (Fig 4D) and monocyte (Fig 4E) activation, as determined by CD11b expression (Eisenhardt *et al*, 2007). C10M reduces pCRP*‐induced monocyte and neutrophil activation, however, this effect was not significant in monocytes, most likely due to CD11b‐involvement in the platelet–leukocyte‐aggregation cascade and the strong effect of ADP on this readout (Fig 4D and E; Cerletti *et al*, 2012; Ghasemzadeh & Hosseini, 2013).

**Figure EV2:**
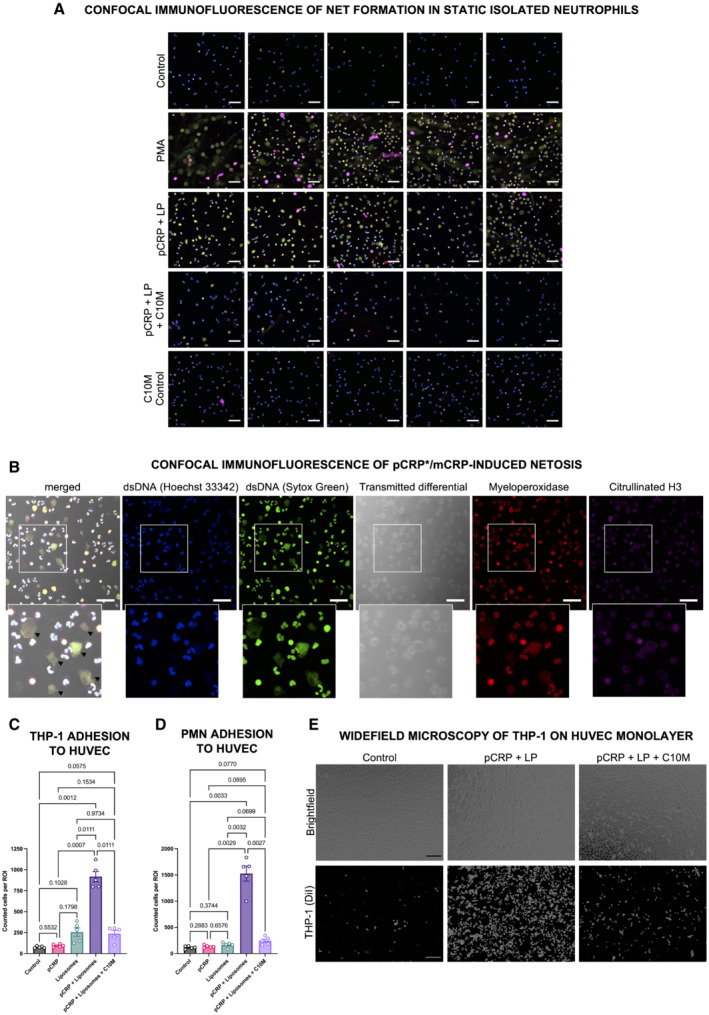
Inhibitory effects of C10M on pCRP*/mCRP‐induced NETosis and leukocyte–endothelial interaction ARepresentative confocal immunofluorescence images of isolated human neutrophils as summarized in Fig 4H. Cells were stained with anti‐dsDNA dyes Hoechst 33342 and Sytox Green, and anti‐MPO and anti‐citH3 antibodies. Shown are uncropped images of merged channels and at 40× magnification. Scale bars indicate 50 μm.BRepresentative single‐channel confocal immunofluorescence images of pCRP*/mCRP‐induced NETosis. Cells demonstrating NETs in the pCRP*/mCRP‐stimulated group showed disrupted cell membranes as visible in the transmitted differential and merged channel (black arrow heads), indicating a suicidal mode of NETosis. Scale bars indicate 50 μm.C–ELeukocyte adhesion on endothelial cells. HUVEC monolayers treated with different isoforms of CRP as described for Fig 4B and C were incubated with fluorescently‐labeled THP‐1 cells (C, E) and neutrophils isolated from human whole blood (PMN, D) for 30 min. After incubation, the monolayer was washed and adherent cells were fixed. THP‐1 cell and neutrophil binding to HUVEC monolayers were then evaluated by automated cell counting in five non‐overlapping ROIs at 10× magnification as demonstrated for THP‐1 in (E). Scale bars indicate 100 μm. Graph shows mean ± SEM. *P* values were calculated with ANOVA and Tukey's *post‐hoc* test. Biological replicates, *n* = 5 for THP‐1 and PMN, respectively. Source data are available online for this figure.

Intriguingly, the pCRP*/mCRP‐induced upregulation of ICAM‐1 and VCAM‐1 in HUVECs was reduced by C10M sufficiently to suppress an aggravated adhesion of the monocytic cell line THP‐1 (Fig EV2C and E) and neutrophils (Fig EV2D) to the monolayer.

Generation of reactive oxygen species (ROS) measured by redox‐indicator dihydroethidium (DHE) in flow cytometry served as another pro‐inflammatory readout in both monocytes and neutrophils (Fig 4F and G). A novel finding of our investigation into the mode of action of CRP‐regulated inflammation is that pCRP*/mCRP induces formation of neutrophil extracellular traps (NETs) (Figs 4H and EV2A), a process called NETosis. NETosis is a mediator of inflammation, a key event that modulates tissue and organ damage (Cahilog *et al*, 2020). Our finding that C10M reduces pCRP*/mCRP‐induced NETosis further highlights the relevance of CRP regulation as an important immune checkpoint and the therapeutic potential of C10M. pCRP*/mCRP induces suicidal NETosis after 3 h of stimulation (Fig EV2B), a mode of NETosis where the membrane integrity is lost during the process (Yipp & Kubes, 2013). However, further analyses of the exact pathway of pCRP*/mCRP‐induced NETosis have to be conducted to ultimately elucidate the underlying mechanism.

### C10M inhibits CRP‐induced aggravation of renal IRI

**Figure 5:**
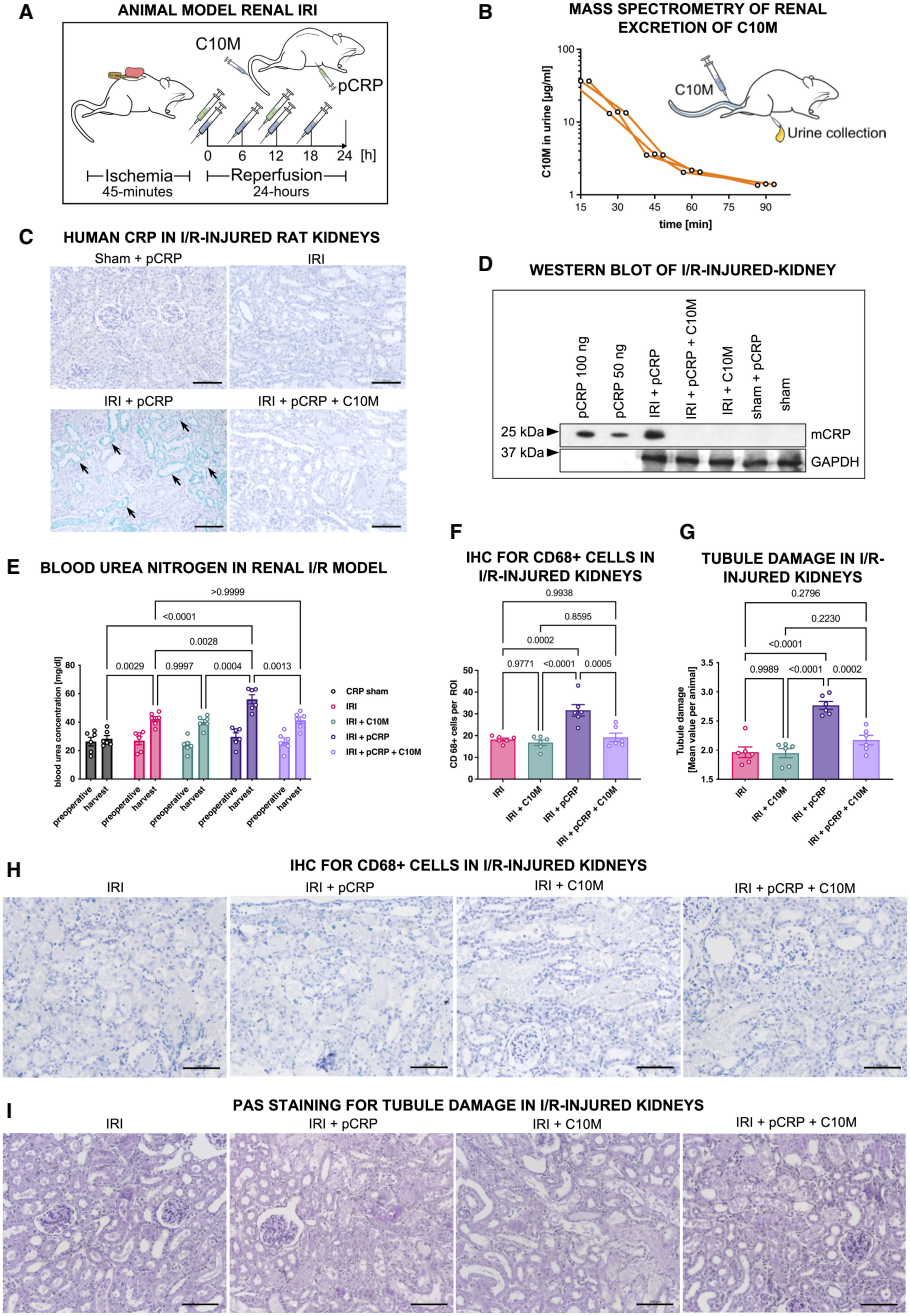
pCRP*/mCRP‐driven exacerbation of renal ischemia/reperfusion injury is reduced by C10M Depiction of the experimental protocol used for ischemic acute kidney injury. Male Wistar rats were subjected to IRI and received i.p. DPBS and pCRP twice (green syringe). C10M was i.v.‐injected separately every 6 h (blue syringe).Detection of C10M by mass spectrometry in rat urine. Renal excretion of C10M detected by mass spectrometry in three rats (biological replicates, *n* = 3) intravenously injected with C10M. Urine samples were collected at the indicated timepoints. 80% of the applied C10M mass was excreted after 90 min (Kather *et al*, 2021).Immunohistochemistry of rat kidneys subjected to IRI and i.p. pCRP application revealed distinct staining by anti‐pCRP*/mCRP‐9C9 antibody (green, arrows). C10M reduces the deposition of total CRP in the impaired tissue. No deposits in the non‐ischemic tissue (sham). Exemplary stainings out of at least three are shown. Scale bars, 100 μm.Tissue lysates of rat kidney were separated on SDS‐PAGE and total CRP was identified with anti‐CRP antibody. A band at the size of mCRP (~ 23 kDa) was detected in kidneys subjected to IRI and pCRP, but not in animals treated additionally with C10M. The household gene protein GAPDH served as a control for loading equal amounts of protein. 50 and 100 ng human pCRP, respectively, served as a positive control. Representative results are shown for replicated assays (*n* = 3).Renal excretion is impaired by pCRP*/mCRP‐driven tissue damage. Blood urea nitrogen (BUN) was utilized as surrogate marker for the excretion function of the kidney. Blood samples were taken before the surgical procedure (preoperative) and 24 h after the procedure (harvest). Graph shows mean ± SEM.Immunohistochemical detection of transmigrated CD68^+^ cells in IRI kidneys. Quantification of immunohistochemical results is shown as mean ± SEM. pCRP (25 μg/ml) increased the number of CD68^+^ cells transmigrated into injured renal tissue significantly, while C10M abolished these effects. Presented are mean cell counts per ROI in each animal.Periodic acid‐Schiff (PAS) stained kidney sections show increased damage after renal IRI in rats when pCRP (25 μg/ml) was injected i.v. The tubulointerstitial injury was quantified by the loss of tubular brush border and by cast formation following an established protocol (Megyesi *et al*, 1998, 2001). Quantification of immunohistochemical results is shown as mean ± SEM.Representative results for the immunohistochemical detection of transmigrated CD68^+^ cells in IRI kidneys. CD68^+^ cells are stained with HistoGreen substrate (green). Scale bars indicate 100 μm.Representative results for the PAS stained kidney sections quantified in (G). Scale bars indicate 100 μm. Data information: Statistical analysis (E–G) was performed with ANOVA and Tukey's *post‐hoc* test. Biological replicates, *n* = 6, precise *P*‐values are given. Source data are available online for this figure.

Ischemia/reperfusion injuries represents the prototypic sterile inflammation in which an exacerbated immune response leads to unwanted tissue damage. We have previously demonstrated that IRI‐associated tissue damage is induced by the pro‐inflammatory forms of human CRP (pCRP* and mCRP) and that the bivalent inhibitor bis‐PC can largely prevent this tissue damage by stabilizing the non‐inflammatory pCRP form (Thiele *et al*, 2018). Thus, an IRI‐induced acute renal injury model in rats represents an established and suitable *in vivo* model to evaluate the therapeutic potential of C10M (Thiele *et al*, 2018). First, the pharmacokinetic plasma half‐life (t_1/2_) of C10M was determined by mass spectrometry to be 90 min in the rat (Fig 5A and B) (Kather *et al*, 2021). C10M is cleared by the kidneys after i.v. administration. After IRI, the rat kidneys were examined for human CRP deposits by immunohistochemistry and Western blotting (Fig 5C and D). Staining for human CRP using the conformation‐specific anti‐pCRP*/mCRP antibody 9C9, which targets an epitope exposed in the pro‐inflammatory forms of human CRP but not in pCRP (Ying *et al*, 1989; Padilla *et al*, 2007; Braig *et al*, 2017), demonstrated deposition of the pro‐inflammatory forms of CRP specifically localized to the IRI‐exposed renal tissue (Fig 5C; Schwedler *et al*, 2003). After i.v. administration of C10M, CRP deposition could not be detected in the tissue. This was further confirmed by Western blots of tissue lysates separated by SDS–PAGE (Fig 5D). The beneficial effects of C10M in IRI were reflected by the significant improvement of excretory renal function as analyzed by blood urea levels (Fig 5E). To obtain further mechanistic data, we assessed the CD68^+^ monocytic cell infiltration (Fig 5F and H) in renal tissue and performed PAS staining of renal tissue (Fig 5G and I). In these assays, administration of pCRP leads to significant increase of IRI‐associated inflammatory cell infiltration and tissue damage that can be blunted by the administration of C10M.

### C10M inhibits CRP‐mediated allograft rejection in hindlimb transplantation

**Figure 6:**
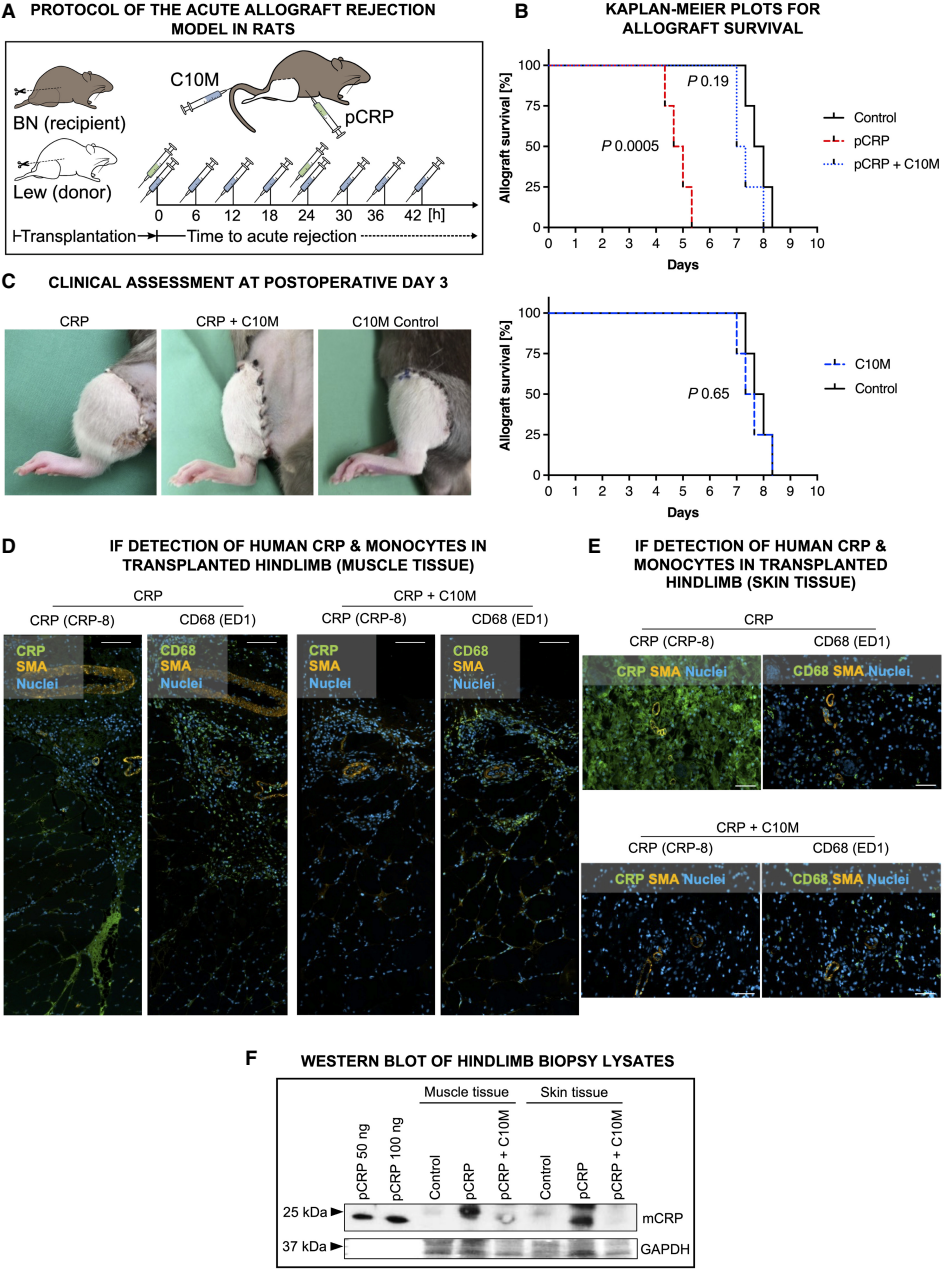
Compound C10M delays CRP‐driven transplant rejection in a hindlimb transplantation model AExperimental protocol of the acute allograft rejection model in rats. Lewis (Lew) rats served as donors, Brown‐Norway (BN) rats as recipients. Hindlimb rejection was assessed clinically and histologically. pCRP (green syringe) and C10M (blue syringe) were injected i.p. and i.v., respectively, at the time‐points indicated.BKaplan–Meier plots for control, pCRP, and pCRP+C10M (above) and C10M vs control (bottom) treatment for total allograft survival with and without CRP ± C10M. Kaplan–Meier curves for different treatments were compared by Mantel–Cox log‐rank test. Allograft survival was significantly reduced by pCRP administration (*P* = 0.0005, median survival control vs. pCRP, 7.8 vs. 4.8 days). C10M masks the CRP‐accelerated hindlimb rejection (median survival 7.2 days). Biological replicates, *n* = 4 per group.CRepresentative photographic examples of transplanted VCA hindlimb allografts. Shown are Lew hindlimb transplants in orthotopic situ on BN recipients 3 days after transplantation. Rats receiving i.p. pCRP presented VCA with massive edema (left). C10M treatment inhibits pCRP‐aggravated early graft rejection (middle). The depicted C10M control without CRP shows no clinical signs of rejection after 5 days (right).D, ERepresentative immunofluorescence images from transplanted grafts reveal distinct signals (green) using anti‐CD68 and anti‐CRP antibodies, respectively. In C10M‐treated animals, less CD68^+^ cells infiltrate the transplanted tissue. Scale bars, 100 μm. (D) Immunofluorescence of transplanted muscle and (E) skin tissue revealed distinct CRP deposition (green) in pCRP‐treated animals, but only minimal staining in rats treated additionally with C10M. Scale bars, 100 μm.FExemplary tissue lysates of muscle and skin probes separated on SDS‐PAGE are depicted. Total human CRP was identified with an anti‐CRP antibody. Source data are available online for this figure.

Ischemia/reperfusion injuries is a major aggravating factor in allograft rejection and damage after allograft transplantation (Dashkevich *et al*, 2016; Nieuwenhuijs‐Moeke *et al*, 2020). To further confirm the therapeutic potential of pCRP*/mCRP inhibition *in vivo*, we performed hindlimb transplantation on fully mismatched rat strains as a model for acute allograft rejection of vascularized composite allografts (VCA) and clinically assessed graft survival (Fig 6A). We found the injection of human pCRP to strongly promote the diapedesis of monocytes and tissue degradation, thereby accelerating VCA‐graft loss significantly compared to a transplanted control group (control vs pCRP, 7.83 vs. 4.83 days; *n* = 4, each group; *P* = 0.0005, log‐rank (Mantel‐Cox) test; Fig 6B, top, black vs red broken line). Most importantly, premature graft loss driven by pCRP was prevented by i.v. C10M application during the first 2 days after transplantation (Fig 6A and B, top, blue broken line). C10M effects are attributable to the inhibition of pCRP* and mCRP, as without extrinsic pCRP application C10M did not show protective effects (Fig 6B, bottom, blue dotted vs. black line). Transplanted rat hindlimbs showed significant clinical signs of rejection (edema, erythema) on day 3 after transplantation post CRP administration (Fig 6C) that were not present in the control group or when formation of pCRP*/mCRP was blocked with C10M. Skin and muscle biopsies were taken at day 3 and analyzed histologically. Monocyte infiltration was detected by immunofluorescence microscopy, which revealed significantly more monocyte infiltrates in VCA tissue of rats treated with pCRP compared to control rats. To investigate whether these exacerbating effects were specific for CRP, C10M was used to block the formation of the pCRP* and mCRP species. In rats treated with both pCRP and C10M, no CRP deposits were detected in either muscle (Fig 6D) or skin tissue (Fig 6E). We found the number of transmigrated CD68^+^ cells to be reduced to control levels when C10M was administered in the pCRP group. Furthermore, we analyzed the amount of deposited human pCRP*/mCRP in the tissue by Western blotting with antibody clone CRP‐8 and found human pCRP*/mCRP significantly reduced in both muscle and skin (Fig 6F; here, the CRP detection was found to run at slightly different sizes, which might correlate with different redox states of the deposited protein; Wang *et al*, 2011). These results indicate that compound C10M inhibits the CRP‐dependent activation and transmigration and thereby abrogates the CRP‐mediated local inflammatory exacerbation in transplant rejection.

### C10M does not suppress CRP‐independent host defense against pathogens

**Figure 7:**
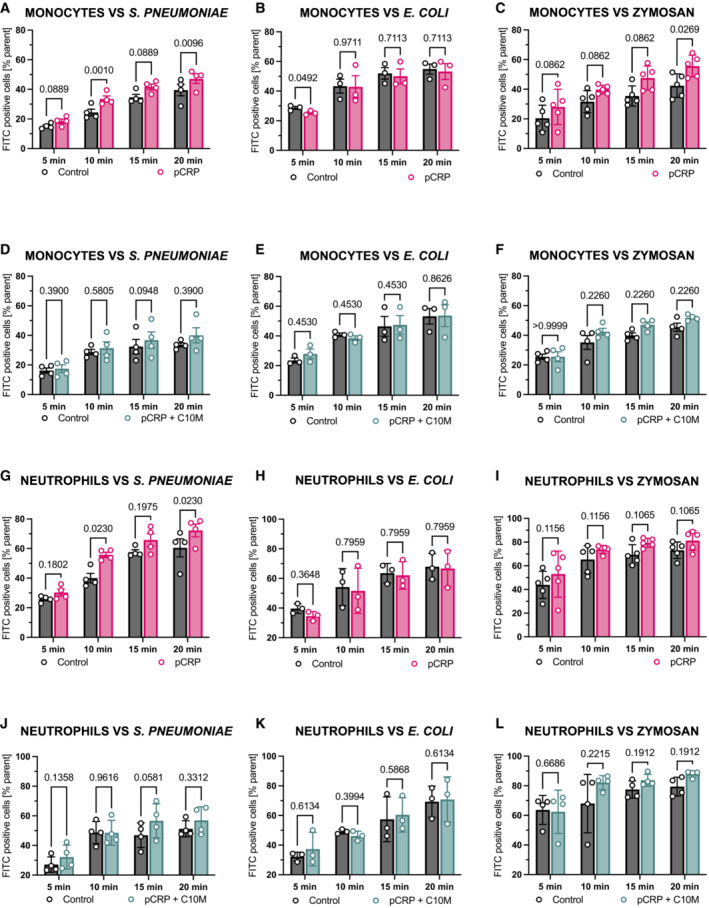
Flow cytometry‐based analysis of CRP‐dependent opsono‐phagocytosis of *S. pneumoniae*, *E. coli*, and zymosan in the presence of C10M Phagocytosis of pCRP‐opsonized, heat‐killed and FITC‐labeled *S. pneumoniae* by human monocytes serves as an exemplary flow cytometry‐based phagocytosis assay. Bar chart shows phagocytic index (percentage of target positive cells of subtype/all cells of subtype) of un‐opsonized (control, dark gray) and pCRP‐opsonized targets (red) after 5, 10, 15, and 20 min, respectively.Phagocytosis of pCRP‐opsonized, heat‐killed and FITC‐labeled *E. coli* by human monocytes serves as a second exemplary flow cytometry‐based phagocytosis assay. Bar chart shows phagocytic index as described above of un‐opsonized (control, dark gray) and pCRP‐opsonized targets (red) after 5, 10, 15, and 20 min, respectively.Phagocytosis of pCRP‐opsonized, heat‐treated, and FITC‐labeled zymosan by human monocytes serves as a third exemplary flow cytometry‐based phagocytosis assay. Bar chart shows phagocytic index as described above of un‐opsonized (control, dark gray) and CRP‐opsonized targets (red) after 5, 10, 15, and 20 min, respectively.Experiments described in (A) were repeated but with targets incubated with pCRP (100 μg/ml) and C10M (1:100 molar ratio) for 30 min, 37°C. Bar chart shows phagocytic index of *S. pneumoniae*‐positive monocytes for un‐opsonized (control, dark gray) and targets incubated with pCRP and C10M (green) after 5, 10, 15, and 20 min, respectively.Experiments described in (B) were repeated but with targets incubated with pCRP (100 μg/ml) and C10M (1:100 molar ratio) for 30 min, 37°C. Bar chart shows phagocytic index of *E. coli*‐positive monocytes for un‐opsonized (control, dark gray) and targets incubated with pCRP and C10M (green) after 5, 10, 15, and 20 min, respectively.Experiments described in (C) were repeated but with targets incubated with pCRP (100 μg/ml) and C10M (1:100 molar ratio) for 30 min, 37°C. Bar chart shows phagocytic index of zymosan‐positive monocytes for un‐opsonized (control, dark gray) and targets incubated with pCRP and C10M (green) after 5, 10, 15, and 20 min, respectively.Phagocytosis of pCRP‐opsonized, heat‐killed, and FITC‐labeled *S. pneumoniae* by human neutrophils. The same blood samples described in (A) were analyzed for the phagocytic index of *S. pneumoniae* by human neutrophils by flow cytometry. Bar chart shows phagocytic index of un‐opsonized (control, dark gray) and pCRP‐opsonized targets (red) after 5, 10, 15, and 20 min, respectively.Phagocytosis of pCRP‐opsonized, heat‐killed, and FITC‐labeled *E. coli* by human neutrophils. The same blood samples described in (B) were analyzed for the phagocytic index of *S. pneumoniae* by human neutrophils by flow cytometry. Bar chart shows phagocytic index of un‐opsonized (control, dark gray) and pCRP‐opsonized targets (red) after 5, 10, 15, and 20 min, respectively.Phagocytosis of pCRP‐opsonized, heat‐killed, and FITC‐labeled zymosan by human neutrophils. The same blood samples described in (C) were analyzed for the phagocytic index of *S. pneumoniae* by human neutrophils. Bar chart shows phagocytic index of un‐opsonized (control, dark gray) and pCRP‐opsonized targets (red) after 5, 10, 15, and 20 min, respectively.Phagocytosis of pCRP+C10M‐treated *S. pneumoniae* by human neutrophils. Results from experiments described in (D) were analyzed for the phagocytic index of *S. pneumoniae* by human neutrophils by flow cytometry. Bar chart shows phagocytic index of *S. pneumoniae*‐positive neutrophils for un‐opsonized (control, dark gray) and targets incubated with pCRP and C10M (green) after 5, 10, 15, and 20 min, respectively.Phagocytosis of pCRP+C10M‐treated *E. coli* by human neutrophils. Results from experiments described in (E) were analyzed for the phagocytic index of *S. pneumoniae* by human neutrophils by flow cytometry. Bar chart shows phagocytic index of *E. coli*‐positive neutrophils for un‐opsonized (control, dark gray) and targets incubated with pCRP and C10M (green) after 5, 10, 15, and 20 min, respectively.Phagocytosis of pCRP+C10M‐treated zymosan by human neutrophils. Results from experiments described in (F) were analyzed for the phagocytic index of *S. pneumoniae* by human neutrophils by flow cytometry. Bar chart shows phagocytic index of zymosan‐positive neutrophils for un‐opsonized (control, dark gray) and targets incubated with pCRP and C10M (green) after 5, 10, 15, and 20 min, respectively. Data information: Bar graphs with individual experiments (A–L) show mean ± SD of the phagocytic index (percentage of target positive cells of subtype/all cells of subtype) of un‐opsonized (control, dark gray), CRP‐opsonized targets (red), and CRP‐opsonized targets in the presence of C10M after 5, 10, 15, and 20 min, respectively. *P* values were calculated using multiple matched *t*‐tests. Biological replicates, *n* = 3–5. Precise *P*‐values are given. Source data are available online for this figure.

**Figure EV3:**
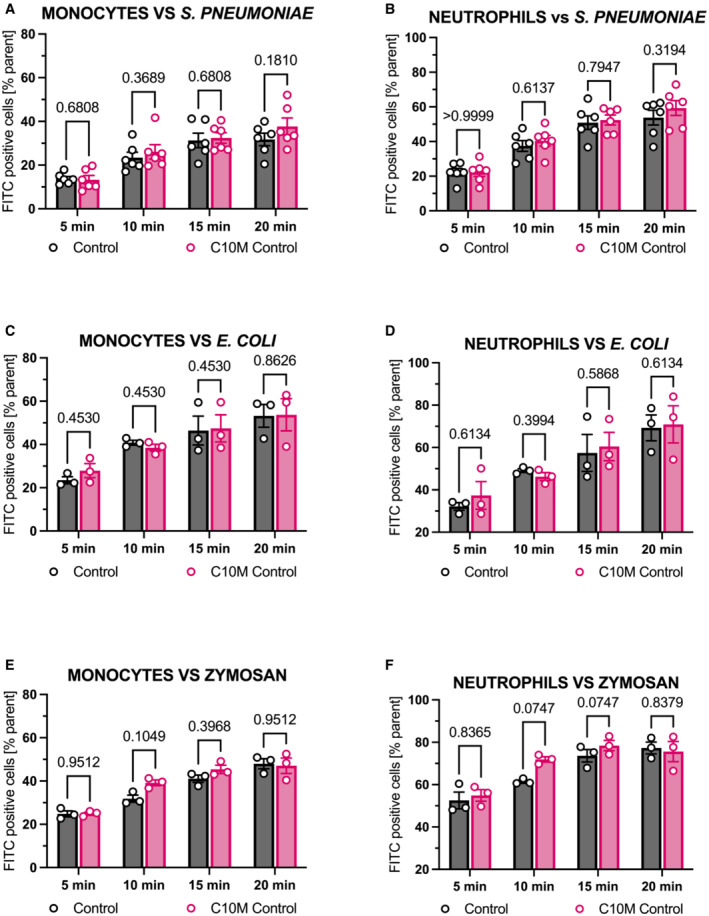
Flow cytometry‐based analysis of phagocytosis of *S. pneumoniae*, *E. coli*, and zymosan in the presence and the absence of C10M A, BPhagocytosis of heat‐killed and FITC‐labeled *S. pneumoniae* by monocytes (A) and neutrophils (B) was analyzed with or without C10M (in equal concentrations used as demonstrated in Fig 7). Bar chart shows phagocytic index (percentage of target positive cells of subtype/all cells of subtype) of an untreated control versus C10M treated cells after 5, 10, 15, and 20 min, respectively. Mean ± SEM are indicated.C, DPhagocytosis assay for heat‐killed and FITC‐labeled *E. coli* by monocytes (C) and neutrophils (D).E, FPhagocytosis of the yeast cell wall ligand zymosan (*Saccharomyces cerevisiae*) by monocytes (E) and neutrophils (F). Mean ± SEM are indicated. Data information: Statistical analysis for all assays shown was performed using multiple matched *t*‐tests. Biological replicates, *n* = 3 and 6. Precise *P*‐values are given. Source data are available online for this figure.

Phagocytosis of bacteria is a crucial protective mechanism of the innate immune response and CRP‐mediated phagocytosis has been previously described (Mold *et al*, 2001). To investigate the effects of C10M on phagocytosis, we challenged human whole blood with *S. pneumoniae*, *E. coli*, and zymosan, respectively, in a flow cytometry‐based phagocytosis assay. pCRP led to an increase in phagocytosis of *S. pneumoniae* serotype 27 in monocytes and neutrophils (Fig 7A and G), which was reduced by the addition of C10M (Fig 7D and J). Intriguingly, serotype 27 expresses PC as part of its capsule (Bennett & Bishop, 1977a, 1977b). Therefore, the capsule makes serotype 27 a target for pCRP opsonization (Edwards *et al*, 1982; Pilishvili *et al*, 2010) and a competitor for the C10M binding. However, there was no significant increase of phagocytosed *E. coli*, a gram‐negative bacterium, in the presence of pCRP nor were effects of C10M measured in monocytes or neutrophils (Fig 7B, E, H, and K), respectively. The effects of pCRP opsonization on phagocytosis of *E. coli* as a gram‐negative bacterium play a minor role (Mold *et al*, 1982), and the innate immune response is based on toll‐like receptors and CRP‐independent activation and opsonization by the complement system (C1q, mannose‐binding lectin, C4b, C3b/iC3b) and immunoglobins (Van Dijk *et al*, 1979; Newman & Mikus, 1985). For zymosan, we detected an increase (albeit non‐significant except for 20 min in monocytes) in phagocytosis in both leukocyte subtypes after pCRP opsonization (Fig 7C and I) and incubation with pCRP and C10M (Fig 7F and L), and the phagocytosis in the absence of pCRP was not affected by C10M for any pathogen target or leukocyte, respectively (Fig EV3). This suggests that the protective capacities of the innate immune system remain largely maintained in CRP targeting therapy, which is in line with earlier reports of clinical studies utilizing a CRP‐lowering approach as CRP ASO and CRP apheresis (Warren *et al*, 2015; Torzewski *et al*, 2020, 2021, 2022; Ries *et al*, 2021; Ringel *et al*, 2021; Schumann *et al*, 2021).

## Discussion

C‐reactive protein is a highly evolutionarily conserved, central player in inflammatory and cardiovascular diseases (Zeller *et al*, 2022). The circulating isoform of CRP, pCRP, binds to PC and PE head groups of bioactive lipids exposed on the membranes of damaged cells and microvesicles, which subsequently leads to the formation of the pro‐inflammatory CRP isoforms, pCRP* and mCRP (Molins *et al*, 2011; McFadyen *et al*, 2018). This mechanism has only recently been identified (Eisenhardt *et al*, 2009a; Thiele *et al*, 2014; Braig *et al*, 2017; McFadyen *et al*, 2018). It transforms a relatively inert molecule, pCRP, to highly pro‐inflammatory molecules, pCRP* and mCRP, both contributing to and aggravating tissue damage (Molins *et al*, 2011; Thiele *et al*, 2014, 2018). Disrupting the interaction between pCRP and PC/PE by blocking the PC binding site, and thereby inhibiting this pro‐inflammatory conformational change, represents a novel and attractive anti‐inflammatory strategy. Targeting the direct interaction between PC/PE and pCRP, we employed a combination of medicinal chemistry and computational modeling to develop a novel tool compound, a low molecular weight inhibitor that binds to the PC/PE binding pocket of pCRP, thereby blocking pCRP binding to exposed PC/PE head groups and consequently blocking the activating conformational change of CRP toward pCRP*/mCRP. Utilizing X‐ray crystallography as a direct protein binding assay, we demonstrate that the small molecule C10M, binds to the PC/PE binding pocket of pCRP. C10M inhibits pCRP*/mCRP‐induced monocyte adhesion, cytokine and ROS production, NET formation as well as pCRP*/mCRP‐mediated upregulation of endothelial ICAM‐1 and VCAM‐1. Most importantly, C10M affords significant protection from CRP‐mediated tissue injury in two distinct pre‐clinical models of inflammation, a model of renal IRI and of hindlimb transplantation.

Our approach contrasts to the previously described mode of action for bis‐PC, a compound that combines two PC moieties into one bivalent molecule. Bis‐PC prevents the formation of pCRP* and mCRP by each of its PC portions binding to the PC/PE binding pocket on two separate pCRP molecules, thereby bringing the two pentamers together with opposing B‐faces (Pepys *et al*, 2006). Within this decamer structure, the pCRP B‐faces are no longer available to bind to exposed PC/PE head groups of bioactive lipids and the pro‐inflammatory conformational change of CRP is blocked (Pepys *et al*, 2006). It had been suggested that the CRP‐inhibitory effect of bis‐PC was primarily due to its bivalency (i.e., two functional PC head groups); however, we hypothesized that a monovalent compound would be able to elicit an anti‐inflammatory response and in contrast to bis‐PC leave the B‐face accessible for protein–protein interaction outside of the PC‐binding site. Here, we explored this hypothesis by designing a low molecular weight (~ 250 Da) monovalent molecule, C10M, which mimics PC by binding to the PC/PE binding pocket on pCRP, thereby stabilizing the inert, non‐inflammatory form of CRP by competitively inhibiting the binding of pCRP to exposed PC/PE head groups of bioactive lipids.

The monovalent compound strategy allows both the A‐face and B‐face, with the exception of the PC binding pocket, of the CRP pentamer to remain available to interacting partner proteins and their associated functions. In contrast, only the A‐face of the CRP pentamer is available for interaction when a bivalent compound like bis‐PC is utilized. Proteins known to interact with the B‐face of pCRP and/or pCRP* include the fibrinogen‐like domain of M‐ficolin, FH‐like protein‐1, and human neutrophils (Buchta *et al*, 1987; Okemefuna *et al*, 2010; Zhang *et al*, 2011). Furthermore, there is evidence that the B‐face of pCRP binds to proteins whose secondary structure is predominantly β‐sheet (e.g., Amyloid‐β1‐38), as well as misfolded or aggregated proteins; and that this interaction is independent of the PC binding pocket and located in the vicinity of the interfaces between the monomers in the CRP pentamer (Singh *et al*, 2009; Hammond *et al*, 2010).

**Figure 8:**
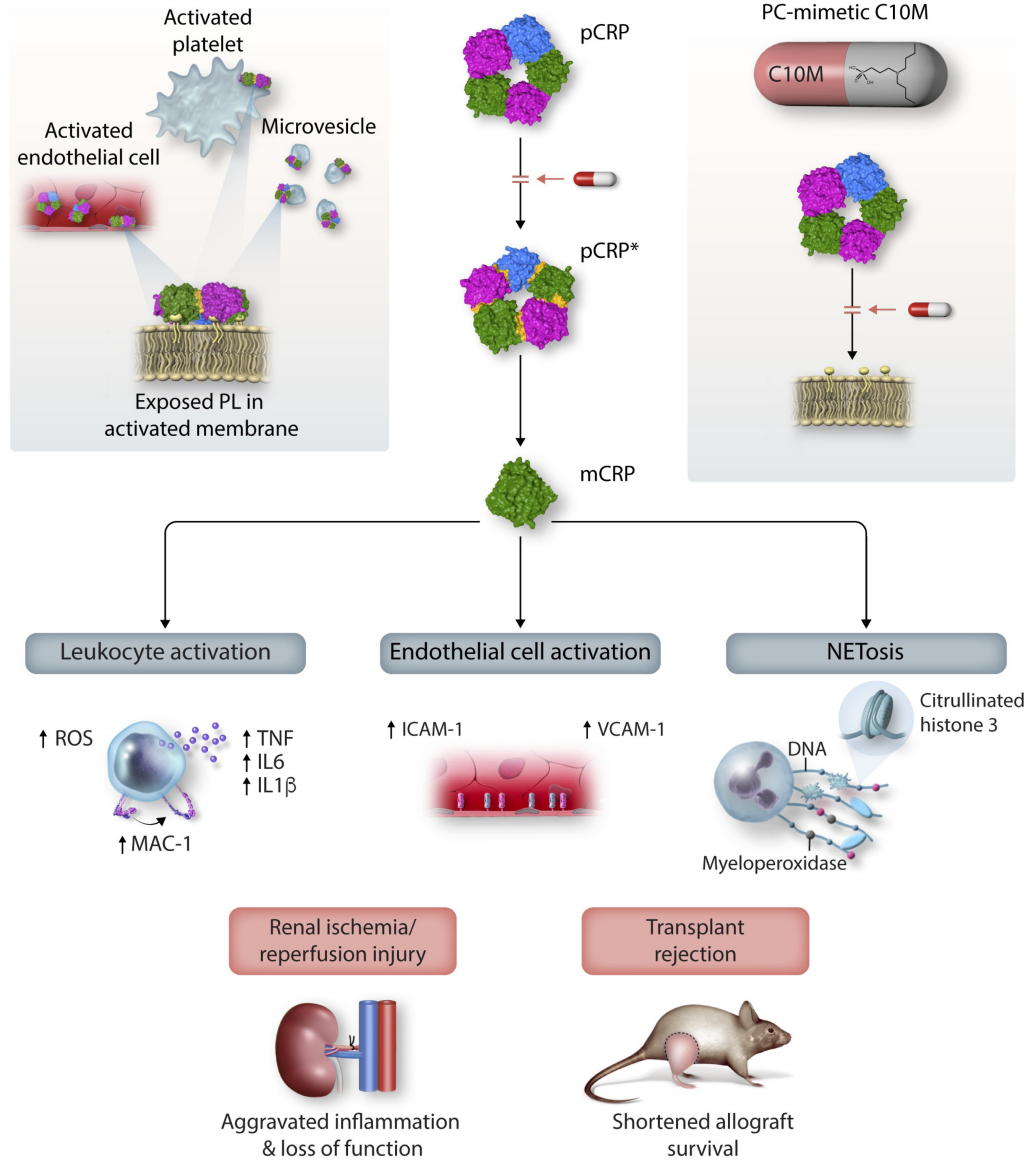
Schematic model of CRP conformational changes and C10M's *in vitro* and *in vivo* anti‐inflammatory effects Exposed phospholipids (PL) in activated cell membranes (e.g., endothelial cells or platelets) or microvesicles contain PC head groups derived from LPC and PE head groups derived from lysophosphatidylethanolamine (LPE), which bind to the PC/PE binding sites on the B‐face of pCRP, thereby anchoring pCRP to the membrane surface. Once bound to the activated membrane, pCRP dissociates into pCRP* and ultimately mCRP. This pro‐inflammatory conformational change results in the activation of leukocytes and endothelial cells as well as NETosis and contributes substantially to renal ischemia/reperfusion injury (IRI) and transplant rejection. Administration of C10M prevents the conformational change of pCRP toward pCRP*/mCRP, the consequent pro‐inflammatory cellular effects and reduces renal IRI and transplant rejection.

The binding mode of C10M to pCRP was confirmed by X‐ray crystallography, showing that the phosphonate moiety was the main anchor point of the compound to pCRP. The C10M:pCRP complex retains the pentameric shape of circulating pCRP and, apart from the occupied PC/PE binding pocket, the remainder of the B‐face is available for partner proteins to interact. The A‐face of the C10M:pCRP complex is unhindered, hence proteins which usually interact with the A‐face of circulating pCRP should be unaffected by the bound C10M. The mode of action of C10M was further investigated and the therapeutic potential of C10M is supported by our following findings (as summarized in the schematic depicted in Fig 8): (i) X‐ray crystallography reveals a specific and stable binding of C10M to the PC/PE binding pocket of pCRP. (ii) C10M binds pCRP and prevents pCRP binding to activated/damaged cell membranes. (iii) In turn, this prevents the conformational change from pCRP to pCRP*/mCRP and their tissue deposition in the area of inflammation. (iv) C10M is therefore inhibiting the exacerbation of inflammation by CRP, rather than being a general anti‐inflammatory drug. (v) C10M reduces tissue damage in a renal IRI model and reduces CRP‐mediated acceleration of allograft rejection. (vi) C10M does not inhibit CRP‐independent phagocytosis suggesting that protective innate immune properties remain intact. This work provides proof‐of‐concept reaching from the design of a novel drug template to *in vitro* and *in vivo* characterization of a novel anti‐inflammatory approach that is based on the most recent understanding of the conformational change leading to pro‐inflammatory isoforms.

pCRP binding to membrane phospholipids of apoptotic cells has been described previously (Gershov *et al*, 2000; Chang *et al*, 2002). The phospholipase A2‐dependent membrane changes in apoptosis and cell activation that lead to the generation of LPC appear to be crucial in mediating pCRP binding to cell membranes, as pCRP neither binds to non‐activated leukocytes (Braig *et al*, 2017) nor the ubiquitous PC head groups in the plasma membrane of healthy living cells. We recently showed that the membrane curvature of healthy cells prevents access to the PC/PE binding pocket of pCRP (Braig *et al*, 2017). Once cell membranes are damaged, the membrane curvature increases, as is the case in microvesicles that avidly bind and transport pCRP*/mCRP (Habersberger *et al*, 2012; Braig *et al*, 2017). Therefore, we chose the PC/PE binding pocket of pCRP as the target for our drug discovery approach. As PC/PE head groups are only accessible for pCRP binding after an inflammatory stimulus or in apoptosis, this therapeutic targeting concept aims at the inhibition of the binding events critical to the generation of pro‐inflammatory CRP isoforms, that is, pCRP* and mCRP. This was considered crucial in our inhibitor design, as inflammation can clearly be beneficial as a defense and repair mechanism of the organism. However, further preclinical and finally clinical studies, besides testing the anti‐inflammatory efficiency, will also have to assess potential side effects such as susceptibility to infections.

Generally, small molecular weight drugs can be tailored for oral administration. An oral anti‐CRP compound could complement the pre‐existing therapy options of either ASO, which would be administered intravenously as typical biologics, or the clinically proven, highly effective CRP‐apheresis, which has been tested in hospitalized patients. Given the potential ability to administer small‐molecule inhibitors via the oral or parenteral route, in addition to having an immediate onset, we assume C10M suitable to complement these existing CRP targeting therapies. However, based on physiochemical properties particularly the presence of a charged phosphonate group, we anticipate that C10M would be poorly bioavailable due to inefficient gut permeability. Charged phosphonates and phosphates in particular are functional groups that have to be incorporated in some small molecular inhibitors as they are crucial for targeting certain biological processes. As such, many chemical strategies are available to develop prodrugs or delivery systems for phosphonates that mask charged groups during absorption, but are unmasked during first‐pass liver metabolism; a promising strategy that could be applied to compounds like C10M (Wiemer, 2020). In contrast, the use of ASO to inhibit CRP expression requires pre‐treatment potentially over weeks making this approach unsuitable for acute applications such as IRI. While CRP apheresis is a highly effective and specific treatment option at removing large amounts of CRP acutely and thus highly attractive in the acute/emergency setting, the use of apheresis takes approximately 5 h and needs repeated runs on successive days depending on the indication and the additional use of anticoagulation, which might be critical in some patients (Torzewski *et al*, 2022). Therefore, a small‐molecule CRP inhibitor is a desirable addition to the established techniques given the potential ease of administration and suitability for acute and chronic indications.

It is important to emphasize that we adopted an approach that provides a selective therapeutic strategy, only inhibiting the uncontrolled exacerbation of inflammation by pCRP*/mCRP but not inflammation *per se*. We confirm this in our *in vitro* monocyte assays, in which we analyze the mode of action of our compound. In these assays, we use pCRP*/mCRP on the surface of ADP‐stimulated platelets to stimulate monocyte adhesion and cytokine expression. Furthermore, we adjusted the initial pCRP concentration in the *in vitro* assays (25 μg and 50 μg per ml) so that we were able to show a significant increase in leukocyte activation (CD11b expression, ROS generation, expression of pro‐inflammatory cytokines) and endothelial cell activation (ICAM‐1 and VCAM‐1 expression, leukocyte adhesion), respectively. Our data confirm our hypothesis that C10M is inhibiting the effects caused by pCRP*/mCRP, but not the increase in inflammation that is induced by ADP‐stimulated platelets, by stabilizing the non‐inflammatory pCRP species.

Further supporting the concept that the basic innate immune response is not affected by C10M, we demonstrate that phagocytosis is not inhibited by C10M. Indeed, neutrophil phagocytosis and killing of bacteria are essential for host defense against bacteria such as pneumococci (Agrawal *et al*, 2008) and pCRP mediates an increased resistance to bacterial infections (Paul Simons *et al*, 2014) via binding to bacterial PC and opsonization of bacteria (Mukerji *et al*, 2012), as demonstrated by the use of *S. pneumoniae* serotype 27. We demonstrate that C10M in the absence of pCRP does not inhibit the phagocytosis of pathogens *S. pneumonia*, *E. coli* or zymosan, respectively, by monocytes and neutrophils. The effects of pCRP opsonization on phagocytosis of *E. coli* as a gram‐negative bacterium (Mold *et al*, 1982) and for zymosan (Lu *et al*, 2008) play a minor role, and the innate immune response is based on toll‐like receptors and CRP‐independent activation and opsonization by the complement system (C1q, mannose‐binding lectin, C4b, C3b/iC3b) and immunoglobins (Van Dijk *et al*, 1979; Newman & Mikus, 1985; Lu *et al*, 2008), suggesting that the protective capacities of the innate immune system remain largely intact in CRP targeting therapy, which is in line with earlier reports of clinical studies utilizing a CRP lowering approach (Warren *et al*, 2015; Torzewski *et al*, 2020, 2021, 2022; Ries *et al*, 2021; Ringel *et al*, 2021; Schumann *et al*, 2021).

Furthermore, the process of NETosis was described to happen as both a vital and a suicidal form with the vital form found to serve specifically anti‐microbial functions (Clark *et al*, 2007; Pilsczek *et al*, 2010; Yipp *et al*, 2012). As part of this study, we showed pCRP*/mCRP‐induced NETosis, which appears to proceed as the suicidal form of NETosis.

This is crucial for an anti‐inflammatory treatment with reduced side effects. These are important observations since a major aim of our therapeutic approach is to target the uncontrolled exacerbation of inflammation rather than inflammation or innate immunity in general.

To validate the effect of C10M *in vivo*, we used two distinct animal models of inflammation. First, we exploited an established renal IRI model in rats (Thiele *et al*, 2018). In this model, the administration of human pCRP at previously described concentrations (25 μg/ml serum) demonstrated an aggravated inflammatory response to IRI (Thiele *et al*, 2014, 2018; Braig *et al*, 2017). We demonstrated that the dissociation of human pCRP to pCRP*/mCRP leads to enhanced leukocyte activation, tissue infiltration, and generation of ROS resulting in aggravation of tissue injury (Thiele *et al*, 2018). This model represents an established model to investigate the anti‐inflammatory properties of C10M as IRI represents the prototypic, sterile inflammatory setting that results in increased tissue damage. Second, the relevance of IRI for allograft survival after tissue transplantation was investigated. The initial inflammatory stage after transplantation is characterized by IRI and has a crucial impact on long‐term allograft survival. Indeed, the infiltration of kidney allografts by macrophages within 10 days of transplantation is associated with worse clinical outcome (Raftery *et al*, 1989; McLean *et al*, 1997). Furthermore, episodes of acute allograft rejection in the first post‐transplantation period have a severe negative impact on long‐term allograft survival (Matas *et al*, 1994). Therefore, we used a well‐described allograft rejection model (hindlimb transplantation as a model of vascularized composite tissue allotransplantation [VCA]; Radu *et al*, 2012) to test the therapeutic potential of C10M. We demonstrate that administration of human pCRP and following dissociation to pCRP*/mCRP accelerates allograft rejection via aggravation of IRI and activation of the innate immune response. In both animal models, we establish C10M's unique benefits in reducing pCRP*/mCRP‐mediated tissue damage. In the VCA model, the acceleration of acute allograft rejection by dissociation of exogenous human pCRP in previously characterized concentrations is reversed by administration of C10M. In the IRI model, renal function is significantly improved and histological signs of kidney injury are markedly reduced. The deposition of human pCRP*/mCRP in the tissue of renal IRI is significantly reduced after administration of C10M, confirming our *in vitro* findings that pCRP binding to activated cell membranes, which we have demonstrated to be a prerequisite for subsequent tissue deposition in the area of inflammation, is reduced (Thiele *et al*, 2014). For our *in vivo* experiments, we utilized rat models. Although rats have abundant pCRP (300–600 μg/ml plasma in normal healthy pathogen‐free rats), rat CRP is not utilized as an acute phase protein and rat complement is not activated by rat CRP (de Beer *et al*, 1982). This is in contrast to human pCRP*/mCRP that activates both rat and human complement, but not mouse complement (Reifenberg *et al*, 2005). Therefore, rats supplemented with human pCRP are a suitable animal model for CRP research (Griselli *et al*, 1999; Gill *et al*, 2004; Pepys *et al*, 2006; Thiele *et al*, 2014, 2018; Braig *et al*, 2017).

The application of human pCRP in rats is a widely accepted animal model, as it mimics most effects human CRP has in humans in a small animal model. Therefore, it has become particularly valuable in models testing therapeutic strategies (Pepys *et al*, 2006) as it is the only model where the final protein target, human pCRP *in vivo*, can be tested. However, the application of human pCRP into the rat has limitations based on the xenogenic nature of this approach and may also not fully reflect CRP's pathophysiological role in humans. Therefore, in the future, additional validation should be conducted in animal models with endogenous CRP, such as rabbits and pigs (Barrett *et al*, 2002; Sheriff *et al*, 2015), to further preclinical drug development. Another limitation of our study is the limited time period over which the effects of pCRP and its inhibition were assessed. CRP inhibition might have to be timed in a tightly fashion as to not interfere with resolution of inflammation required after the acute inflammatory disease state.

While purified native human pCRP itself is described not to be pro‐inflammatory when injected into healthy individuals (Lane *et al*, 2014), earlier studies using recombinant human CRP reported endothelial dysfunction and augmented pro‐coagulant responses, which were not attributed to potential LPS contaminations (Bisoendial *et al*, 2005, 2007). Notably, purified native human pCRP injected in healthy rabbits showed a significant blood pressure drop (Bock *et al*, 2020). These partially contradictory findings highlight the need for further research on the direct effects of pCRP.

Importantly, C10M i.v. application was successful in obtaining protective effects in two animal models of severe, localized inflammation. The development of the tool compound C10M as a novel small‐molecule inhibitor of human pCRP provides important proof‐of‐concept that inhibition of pCRP conformational change toward pro‐inflammatory isoforms, that is, pCRP*/mCRP represents a highly effective anti‐inflammatory approach and paves the way for future design of pharmacologically tailored anti‐inflammatory drugs. However, C10M is not designed to interfere with either mCRP or signaling through Fcγ receptors or lipid rafts. The design of C10M rather aims to inhibit the interactions that result in the formation of pCRP*/mCRP. In conclusion, competitively blocking the PC/PE binding pocket on pCRP is a highly promising and attractive strategy towards reduction of CRP‐mediated inflammation. Given the wide range of clinical conditions where pCRP*/mCRP‐mediated tissue damage has been demonstrated, the therapeutic targeting of pCRP with small‐molecule inhibitors is likely to be of broad clinical relevance and can potentially be used in many diseases driven by inflammation.

## Materials and Methods

### Reagents and Tools table

**Table:** 

Reagent/resource	Reference or source	Identifier or catalog number
**Experimental models**		
Rat (rattus norvegicus)	Charles‐River Laboratories, Research Models and Services, Sulzfeld, Germany	Brown‐Norway; male
Rat (rattus norvegicus)	Charles‐River Laboratories, Research Models and Services, Sulzfeld, Germany	Lewis; male
Rat (rattus norvegicus)	Charles‐River Laboratories, Research Models and Services, Sulzfeld, Germany	Wistar; male
THP‐1 (*human monocytic cell line*)	DKMZ, Braunschweig, Germany	1‐year‐old boy with AML; https://www.dsmz.de/collection/catalogue/details/culture/ACC‐16
HUVEC (*human umbilical vein endothelial cell*)	PromoCell, Heidelberg, Germany	https://promocell.com/product/human‐umbilical‐vein‐endothelial‐cells‐2‐huvec‐2/
**Antibodies**		
pCRP*/mCRP (clone 9C9); 1:10	Prof Lawrence A. Potempa, College of Pharmacy, Roosevelt University, Schaumburg, IL, USA; lpotempa01@roosevelt.edu	Ying *et al* (1989)
pCRP (clone 8D8); 1:10	Prof Lawrence A. Potempa, College of Pharmacy, Roosevelt University, Schaumburg, IL, USA; lpotempa01@roosevelt.edu	Ying *et al* (1989)
CRP (clone CRP‐8); 1:100 (IF, IHC), 1:2,000 (WB)	Sigma‐Aldrich	Ying *et al* (1989)
Goat anti‐mouse antibody; 1:5,000 (WB)	BD Biosciences	558121
CD11b (clone ICRF44); 1:20 (FC)	BD Biosciences	562793
CD14 (clone M5E2); 1:50 (FC)	BD Biosciences	558121
CD16 (clone 3G8); 1:50 (FC)	BD Biosciences	555407
HLA‐DR (clone TU36); 1:50 (FC)	BD Biosciences	555560
CD2 (clone RPA‐2.10); 1:50 (FC)	BD Biosciences	555327
CD15 (clone VIMC6); 1:50 (FC)	BD Biosciences	562371
CD19 (clone HIB19); 1:50 (FC)	BD Biosciences	555413
CD56 (clone MY31); 1:50 (FC)	BD Biosciences	347747
NKp46 (clone BAB281); 1:50 (FC)	BD Biosciences	557991
TNF (clone MAb11); 1:33 (FC)	Invitrogen, Thermo Fisher	14‐7349‐81
IL1β (clone REA1172); 1:50 (FC)	Miltenyi Biotec	130‐120‐951
IL6 (clone MQ2‐13A5); 1:50 (FC)	Miltenyi Biotec	130‐096‐093
CD54 APC (clone HA58); 1:50 (FC)	BD Biosciences	752310
CD106 PE (clone 51‐10C9); 1:50 (FC)	BD Biosciences	746536
MPO (clone REA491); 1:50 (FC)	Miltenyi Biotec	130‐120‐241
CD41a APC (clone HIP8); 1:20 (FC)	Thermo Fisher	12‐0419‐42
SMA (clone 1A4); 1:200 (IF)	Sigma‐Aldrich	C6198
CD68 (clone ED‐1); 1:100 (IF)	Merck	MAB1435
Anti‐mouse IgG (H+L)‐CF^TM^ 488; 1:200 (IF)	Sigma‐Aldrich	SAB4600388
GAPDH HRP (0411); 1:1,000 (WB)	Santa Cruz Biotechnology	Sc‐47724
CD62P PE (Clone AK‐4); 1:5 (FC)	BD Pharmingen	555524
Goat anti‐human MPO (polyclonal); 1:80 (IF)	R&D Systems	AF3667
Rabbit anti‐citrullinated histone 3 (polyclonal); 1:100 (IF)	Abcam	Ab5103
Goat anti‐rabbit IgG AF647 (polyclonal); 1:2,000 (IF)	Thermo Fisher	A‐21245
Donkey anti‐goat IgG AF546 (polyclonal); 1:2,000 (IF)	Thermo Fisher	A‐11056
**Chemicals, enzymes and other reagents**		
Human C‐reactive protein from human fluids (used for cell assays and animal studies)	Calbiochem, EMD Millipore Corp.	https://www.merckmillipore.com/AU/en/product/C‐Reactive‐Protein‐Human‐Ascites,EMD_BIO‐236600
Recombinant human C‐reactive protein (used for biophysical assays and crystallization studies)	Merck, Germany	https://www.sigmaaldrich.com/AU/en/product/sigma/c4063
p‐aminophenyl phosphoryl choline agarose beads	Thermo Fisher	https://www.thermofisher.com/order/catalog/product/20307
Pierce™ 0.8 ml centrifuge columns	Thermo Fisher	https://www.thermofisher.com/order/catalog/product/89868
C10M (*3‐*(*dibutylamino*)*propyl*) *phosphonic acid*)	Anthem Biosciences, India	Please contact the authors
SYTOX™ orange nuclei acid stain	Thermo Fisher	https://www.thermofisher.com/order/catalog/product/S11368
CellFIX™ cell fixation solution	BD Biosciences	https://www.bdbiosciences.com/en‐de/products/reagents/flow‐cytometry‐reagents/clinical‐diagnostics/buffers‐and‐supporting‐reagents‐ivd‐ce‐ivd/bd‐cellfix‐10x‐concentrate.340181
Endothelial Cell Basal Medium (SupplementMix, PromoCell).	PromoCell, Heidelberg, Germany	https://www.bdbiosciences.com/en‐de/products/reagents/flow‐cytometry‐reagents/clinical‐diagnostics/buffers‐and‐supporting‐reagents‐ivd‐ce‐ivd/bd‐cellfix‐10x‐concentrate.340181
**Software**		
GraphPad Prism v9.0 for Mac	GraphPad Software, La Jolla, California, USA	Statistical Analysis
IMARIS image visualization and analysis software	Oxford Instruments, Abingdon, UK	3D reconstructions of confocal immunofluorescence images
ImageJ	Schneider *et al* (2012)	Image analysis
PyMOL Molecular Graphics System Version 2.0	Schrodinger, LLC (https://pymol.org)	
Marvin version 20.20.0	ChemAxon (https://www.chemaxon.com)	
PKSolver add‐in for Microsoft Excel Excel	Zhang *et al* (2010)	
**Other**		
Qubit® 3.0 Fluorometer, Invitrogen™	Life Technologies™, Carlsbad, CA, USA	https://tools.thermofisher.com/content/sfs/manuals/qubit_3_fluorometer_man.pdf
Histopaque 1077 and Histopaque 1119	Sigma‐Aldrich, St. Louis, MO	
Lipopolysaccharide (LPS, *E. coli* serotype O127:B8)	Sigma‐Aldrich, St. Louis, MO	
ibidi® μ‐slides VI 0.4 (tissue culture treated polymer μ‐slides)	ibidi GmbH, Planegg, Germany	https://ibidi.com/channel‐slides/57‐‐slide‐vi‐04.html
26G catheter	Abbocath‐ T, ICU Medical B.V., Netherlands	
Isoflurane	Abbott, Wiesbaden, Germany	
Nylon suture (9/0)	Serag‐Wiessner, Naila, Germany	
BD Micro‐Fine^TM^ +Demi, 30G insulin syringes	BD Medical, NJ, USA	https://www.bd.com/en‐uk/products/diabetes/diabetes‐products/insulin‐syringes/microfine‐insulin‐syringes
Slide‐A‐Lyzer™ Dialysis Cassettes, 10k MWCO	Pierce Biotechnology, Rockford, IL USA	https://www.thermofisher.com/order/catalog/product/66380
CM5 chip (Biacore S200)	GE Healthcare	https://www.cytivalifesciences.com/en/us/shop/protein‐analysis/spr‐label‐free‐analysis/spr‐consumables/sensor‐chips/sensor‐chip‐cm5‐p‐05858
ECLTM Western blotting analysis system	GE Healthcare, UK	
Medical x‐ray film	Fujifilm, Japan	
CURIX 60 developer	AGFA	

### Methods and Protocols

#### Experimental design

The homo‐pentamer and acute phase protein CRP (pCRP) amplifies tissue injury in the context of inflammation and ischemia in a conformation‐specific manner. The “CRP cascade” localizes and amplifies inflammation via dissociation of the native pCRP, which is mainly a functionally inert molecule, to the highly pro‐inflammatory isoforms pCRP* and monomeric CRP (mCRP). The binding of pCRP to PC and phosphoethanolamine (PE) head groups exposed on the surface of activated and damaged cells initiates the process of the pro‐inflammatory “CRP cascade.” We designed a low molecular weight tool compound, C10M, modeled after the structure of PC and shown by X‐ray crystallography to bind to the PC binding pocket in pCRP. C10M is able to block pCRP from binding to damaged cells/tissue and inhibits the formation of the pro‐inflammatory CRP isoforms, pCRP* and mCRP, and thereby prevents conformational changes in pCRP. This novel CRP inhibiting therapeutic approach demonstrates strong anti‐inflammatory effects *in vitro* and *in vivo*, the latter shown in a rat model of IRI and a rat model of VCA hindlimb transplantation. Inhibiting the binding of pCRP to PC/PE via low molecular weight compounds represents a novel, broadly applicable therapeutic approach for many inflammation‐driven diseases.

#### Synthesis of compound C10M

##### Schematic: synthetic route for C10M

Synthesis of compound C10M was carried out by Anthem Biosciences (India) using standard synthetic methods as follows:

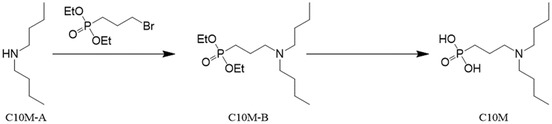

##### C10M‐B diethyl‐(3‐(dibutylamino)propyl)phosphonate.

To a solution of dibutylamine (Sigma Aldrich, 0.32 g, 2.5 mmol) in dimethylformamide (8 ml) was added sodium iodide (0.03 g, 0.25 mmol), potassium carbonate (1 g, 7.5 mmol), and diethyl‐(3‐bromopropyl)‐phosphonate (Sigma Aldrich, 1.42 g, 5.5 mmol) at 0°C. Reaction mixture was heated to 100°C for 14 h. The progress of the reaction was monitored by thin layer chromatography. Reaction mixture was cooled to 0°C and quenched with water (15 ml) and extracted with ethyl acetate (15 ml × 2). Combined organic layer was washed with brine (20 ml), dried over sodium sulfate, and concentrated under vacuum. The residue was separated by silica gel chromatography to provide C10M‐B diethyl‐(3‐(dibutylamino)propyl)‐phosphonate (215 mg, 0.7 mmol) in 28% yield.

##### Tool compound C10M (3‐(dibutylamino)propyl)phosphonic acid

To a solution of diethyl‐(3‐(dibutylamino)propyl)phosphonate (0.28 g, 0.91 mmol) in dichloromethane (25 ml) was added trimethylsilyl bromide (2.79 g, 18.2 mmol, 20 eq) at 0°C. Reaction mixture was heated to 45°C for 14 h. Solvent was concentrated completely. Residue was dissolved in methanol (20 ml) and dichloromethane (20 ml), solvents were then removed under vacuum. The residue was triturated under dichloromethane:hexane (1:1) (30 ml) and heated with stirring at 40°C for 30 min. The solvents were decanted and the residue was dried under vacuum to provide C10M (3‐(dibutylamino)propyl)phosphonic acid as a bromide salt (100 mg, 0.4 mmol) in 44% yield and > 95% purity as determined by HPLC. ^1^H nmr (D_2_O) δ = 3.08 ppm (m 6H, CH_2_N), 1.85 (m 2H, CH_2_P), 1.60 (m 6H, CH_2_), 1.31 (m 4H 7.2 Hz CH_2_), and 0.82 (t 7.2 Hz 6H, CH_3_). ^13^C nmr (D_6_ DMSO) δ = 52.6 ppm (CH_2_N), 25.4 (CH_2_), 19.8 (CH_2_), and 14.0 (CH_3_) MS/+ve ESI Calc C_11_H_26_NO_3_P [M + H] + = 252.1; found [M + H] + = 252.1.

#### Reagents and antibodies

As described previously by our group (Braig *et al*, 2017), we used the conformation‐specific CRP antibodies anti‐mCRP‐9C9 and anti‐pCRP‐8D8 (purified hybridoma culture supernatants; Ying *et al*, 1989). Mouse anti‐human CRP antibody (clone CRP‐8) was purchased from Sigma‐Aldrich. Mouse anti‐human CD14 Pacific Blue™ (clone M5E2), anti‐human CD16 phycoerythrin‐cyanine 7 (PE‐Cy7, clone 3G8) and anti‐human HLA‐DR allophycocyanin (APC, clone TU36) antibodies were purchased from BD Pharmingen™, BD Biosciences. Used lineage markers for exclusion: Mouse anti‐human CD2 PE (clone RPA‐2.10), anti‐human CD15 PE (clone VIMC6), anti‐human CD19 PE (clone HIB19), anti‐human CD56 PE (clone MY31), and anti‐human NKp46 PE (clone BAB281) were from BD Pharmingen™, BD Biosciences. For intracellular cell staining anti‐human TNF fluorescein isothiocyanate (FITC, clone MAb11, Invitrogen), IL1 beta (IL1β) FITC (clone REA1172, Miltenyi Biotec), and IL6 FITC (clone MQ2‐13A5, Miltenyi Biotec) were used. Mouse anti‐human CD54 APC (intercellular adhesion molecule 1, ICAM‐1, clone HA58), anti‐CD62P PE (clone AK‐4) and CD106 PE (vascular cell adhesion molecule 1, VCAM‐1, clone 51‐10C9) were obtained from BD Pharmingen™, BD Biosciences. Recombinant anti‐human myeloperoxidase FITC (MPO, clone REA491, Miltenyi Biotec), mouse anti‐human CD41a APC (clone HIP8, Thermo Fisher), mouse anti‐human α‐smooth muscle Cy3 antibody (SMA clone 1A4, Sigma‐Aldrich), mouse anti‐rat CD68 (clone ED‐1, Merck), goat anti‐mouse IgG (H + L)‐CF™488 antibody (polyclonal, Merck), and dsDNA binding SYTOX™ orange (Thermo Fisher) were used for widefield and confocal fluorescence microscopy. For Western blotting, anti‐glyceraldehyde‐3‐phosphate dehydrogenase horseradish peroxidase (GAPDH HRP) (0411) was purchased from Santa Cruz Biotechnology (Dallas, TX, USA). Lipopolysaccharide from *Escherichia coli* serotype O127:B8 (LPS) and phosphatase substrate were obtained from Sigma‐Aldrich. For the biophysical assays and crystallization studies, pCRP was commercially acquired from Merck (Product number: 236608). For the cell assays and animal studies, human pCRP purified from human ascites was purchased from Calbiochem (EMD Millipore Corp., Germany, Product number: 236600) and prepared as described below.

#### Preparation of pCRP

Preparation of human pCRP was performed as described previously by our group (Braig *et al*, 2017). Human CRP was purchased from Calbiochem (EMD Millipore Corp.) at a protein concentration of 1.03 mg/ml in liquid form suspended in 150 mM NaCl, 20 mM Tris, 2 mM CaCl_2_, 0.1% NaN_3_ at pH 7.5. Protein purity was determined by a single band in SDS‐PAGE. The addition of NaN_3_ to commercially available CRP preparations was reported to attribute to falsely claimed pro‐inflammatory potential of pCRP (Griselli *et al*, 1999; Volanakis, 2001; Lane *et al*, 2014). Hence, for unaffected analysis of CRP effects, native CRP was dialyzed to eliminate contaminations prior to every experiment. Slide‐A‐Lyzer™ Dialysis Cassettes, 10 k MWCO (Pierce Biotechnology, Rockford, IL, USA) were used as recommended by the producer. The pCRP solution was dialyzed against *Dulbecco*'s phosphate‐buffered saline (DPBS) supplemented with calcium and magnesium (supplemented DPBS) in a 1:1,000 v/v ratio at 4°C and overnight, as a modification of a protocol described previously (Gill *et al*, 2004; Pepys *et al*, 2006). The pCRP solution was placed in the pretreated dialysis cassette and dialyzed in 500 ml 4°C cold supplemented DPBS under constant stirring using a magnetic stir bar and a beaker. After 2 h, the dialysis solution was discarded and fresh 500 ml 4°C cold supplemented DPBS was added and the pCRP solution was stored at 4°C overnight. The protein concentration of the purified native pCRP was determined as described below and afterwards stored at 4°C in a polypropylene tube until use for a maximum of 20 days.

#### Preparation of monomeric C‐reactive protein (mCRP)

Monomeric CRP was generated by treating dialyzed pCRP with 8 M urea and 100 mM EDTA for 1 h at 37°C, followed by dialysis against 25 mM Tris–HCl (pH 8.5) overnight at 4°C as described by Bíró *et al* (2007). The protein concentration was determined after each dialysis and dissociation procedure by a benchtop fluorometer (Qubit® 3.0 Fluorometer, Invitrogen™ by life technologies™, Carlsbad, CA, USA).

#### Competitive solid column affinity chromatography for C10M binding to pCRP

pCRP (Calbiochem, EMD Millipore Corp., Germany) binding to the ligand PC was analyzed in the presence of C10M by solid column chromatography as described by Volanakis *et al* (1978). 200 μg human pCRP was incubated with ready‐to‐use 80 μl p‐aminophenyl phosphoryl choline agarose beads (Thermo Fisher) in binding buffer (0.1 M Tris, 0.1 M NaCl, 2 mM CaCl_2_, pH 8) with and without C10M (1:100 molar ratio, pCRP:C10M) for 1 h at room temperature. Pierce™ 0.8 ml centrifuge columns (Thermo Fisher) were used following the manufacture's protocol. Bound pCRP was eluted with elution buffer (0.1 M Tris, 0.1 M NaCl, 2 mM EDTA, pH 8) and collected in fresh tubes and measured by a fluorometric protein assay (Qubit® 3.0 Fluorometer). The data were expressed as mean ± SEM (*n* = 3).

#### Crystallization and X‐ray crystallography

The pCRP protein (Merck, Product number: 236608) was prepared for crystallization by adding 1 μl 100 mM CaCl_2_ to 50 μl pCRP at 8 mg/ml in 20 mM Tris, 140 mM NaCl, 2 mM CaCl_2_. Compound C10M was dissolved in water and added to pCRP to achieve a 1:3 molar ratio of pCRP:C10M. Drops of 2 μl size (1:1 volume ratio of well buffer and protein+C10M) were set up over 500 μl reservoir solution at 20°C in a 24 well Linbro plate using hanging drop vapor diffusion methods. Crystals of pCRP in complex with C10M were obtained with 100 mM Tris pH 9, 10% PEG 4000, 50 mM LiCl and 200 mM MgCl_2_ as the well buffer. The crystals were frozen using liquid nitrogen after application of 20% ethylene glycol as cryoprotectant. Diffraction data were collected on the MX2 beamline at the Australian Synchrotron. The data were processed with XDS (Kabsch, 2010) and CCP4 (McCoy *et al*, 2007). The structure was solved by molecular replacement using the previously published structure of the PC:pCRP complex as the input model (PC molecules were removed, PDB ID: 1B09; Thompson *et al*, 1999). Refinement was performed using PHENIX (Adams *et al*, 2010). The PDB coordinates have been deposited in the Protein Data Bank under PDB ID: 7TBA. The PyMOL Molecular Graphics System Version 2.0 Schrodinger, LLC (https://pymol.org) was used for visualization and preparing the structural images of pCRP in complex with PC and C10M. Marvin was used for drawing chemical structures and reactions, Marvin version 20.20.0, ChemAxon (https://www.chemaxon.com).

#### Human *ex vivo* studies

Whole blood and cells isolated from peripheral venous blood used in the assays described hereafter were taken from healthy human volunteers after informed consent. All human studies were approved by the Ethics Committee of the University of Freiburg Medical Center (# 112/17) and conducted in accordance with the principles set out in the WMA Declaration of Helsinki and the Department of Health and Human Services Belmont Report.

#### *In vitro* and *ex vivo* testing of C10M

All cell cultures were tested for mycoplasma contamination on a regular basis. HUVECs were purchased from PromoCell (Heidelberg, Germany) and cultured in supplemented Endothelial Cell Basal Medium (SupplementMix, 10% FCS, 50 U/ml penicillin, 50 μg/ml streptomycin; PromoCell). The acute monocytic leukemia cell line THP‐1 (DKMZ, Braunschweig, Germany) was used for microvesicle preparation and was cultured in RPMI 1640 medium supplemented with 10% FCS, 2 mM L‐glutamine and 50 U/ml penicillin and 50 μg/ml streptomycin. Identity of the utilized cell line was confirmed by *Multiplex Human Cell Line Authentication Test* (Multiplexion, Heidelberg, Germany). Peripheral blood human primary monocytes were isolated from peripheral whole blood using density gradient centrifugation. Blood was taken from normal and healthy volunteers (aged 22–29, male and female).

#### Binding of pCRP to ADP‐stimulated platelets

Platelet‐rich plasma (PRP) was isolated from freshly taken human whole blood anticoagulated with 3.2% trisodium citrate solution as described elsewhere (Cazenave *et al*, 2004; Jarvis, 2004). Two‐thirds of the PRP were transferred into 1:15 v/v *Tyrode's* buffer supplemented with 8.9 mM EDTA. Briefly, PRP was obtained from fresh anticoagulated human blood by centrifugation at 250 *g* without brake for 10 min and room temperature. The supernatant was designated PRP. The supernatant was then washed two times against sequestrene buffer (17.5 mM Na_2_HPO_4_, 8.9 mM Na_2_EDTA, 154 mM NaCl, pH 6.9) 1:10 v/v. Contaminating white blood cells were depleted by a first centrifugation step at 200 *g* for 10 min with no brake applied. The supernatant was transferred into a fresh tube and centrifuged at 2,000 *g* for 10 min without brake at room temperature. The pellet was resuspended in fresh *Tyrode's* buffer containing 8.9 mM EDTA and centrifuged again under same conditions. The second pellet was resuspended in DPBS and counted using a hemocytometer and adjusted to 2 × 10^8^ platelets/ml. 100 μl of the solution was incubated with 50 μg/ml pCRP‐Atto 594 with or without 13 mM CaCl_2_ for 15 min at 37°C, 5% CO_2_. 20 μl of anti‐CD62P PE (1:5; clone AK‐4, BD Pharmingen™) were added to each sample and transferred to 350 μl FACS buffer 0.5% bovine serum albumin (BSA) and fixed with 50 μl 4% paraformaldehyde in PBS. Flow cytometry was performed within 30 min.

For confocal fluorescence microscopy, samples were prepared as described above and transferred into tissue culture treated polymer μ‐slides (ibidi® μ‐slides VI 0.4). Slides incubated for 1 h at 37°C, 5% CO_2_ and were washed with supplemented DPBS, then fixed with 4% paraformaldehyde in PBS and embedded in mounting medium (ibidi®). Slides were analyzed by confocal fluorescence microscopy using a Zeiss LSM 710 and processed using IMARIS image visualization and analysis software (Oxford Instruments).

For SDS–PAGE, platelets were isolated as described and treated with 20 μM ADP. 50 μg/ml pCRP with or without C10M (molar ratio as described above) and C10M alone were added to the platelets, with one sample untreated (control). After 15 min, all platelet samples were washed in supplemented DPBS 3 times at 2,000 *g* for 10 min with brakes at room temperature. Samples were then treated as described hereafter and analyzed by densitometric quantification of the protein bands in Western blots (*n* = 3) using ImageJ (Schneider *et al*, 2012).

#### Static monocyte adhesion assay

The inhibitory property of C10M on CRP isotypes was tested in a static monocyte adhesion assay as described previously with minor modifications (Eisenhardt *et al*, 2009a; Thiele *et al*, 2014). Briefly, human monocytes were isolated by Ficoll density gradient centrifugation. Greiner® 96‐well reader plates were covered with 100 μl/well of a 20 μg/ml fibrinogen solution in DPBS supplemented with calcium and magnesium overnight at 4°C. Unspecific binding to the exposed polystyrene surface was prevented by 0.1% agarose gel solution. Wells were washed from excessive agarose with supplemented DPBS. The cell pellet containing isolated monocytes was resuspended in DPBS supplemented with calcium and magnesium, cell count was determined manually by hemocytometer and automated by CASY cell counter and adjusted for 3 × 10^6^ cells/ml. 100 μl of the designated cell suspension was pipetted in triplicate and allowed to adhere for 35 min at 37°C, 5% CO_2_. Cells were stimulated with pCRP (50 μg/ml) with or without C10M (1:100 molar ratio). Quantification of monocyte adhesion was performed as described previously by the addition of a phosphatase substrate stain (50 mM sodium acetate pH 5.0, 1% Triton X‐100 and 40 mg of phosphatase substrate) and the absorbance was measured after 30 min at 405 nm by spectroscopy after extensive washing (Eisenhardt *et al*, 2009a).

#### ICR for cytokines in human whole blood

Heparinized human whole blood was freshly taken with informed consent from healthy donors without any drug intake history of anti‐inflammatory medication in at least the last 2 weeks. The CRP‐dependent expression of TNF, IL1β, and IL6 in human whole blood was assessed by ICR and flow cytometry using BD LSR Fortessa Cell Analyzer (BD Biosciences). We have previously demonstrated that upon binding to ADP‐activated platelets pCRP undergoes transitional conformational changes (Eisenhardt *et al*, 2009a; Braig *et al*, 2017) and the pro‐inflammatory isoforms of CRP lead to increased expression of TNF, IL1β, and IL6 in CD14^+^ monocytes (Kiefer *et al*, 2021).

#### Determination of the relevant CRP conformation for pro‐inflammatory cytokine expression

100 μl samples were incubated with 50 μg/ml pCRP, ADP, and C10M, respectively, or stayed unstimulated (*control*) for 6 h at 37°C, 5% CO_2_. After the first 30 min, 3 μg/ml brefeldin A was added to each sample to inhibit the cytokine excretion (O'Neil‐Andersen & Lawrence, 2002). Sedimentation of the cellular portion of the blood was prevented by gentle vortex mixing every 60 min. CellFIX™ cell fixation solution (BD Biosciences) was prepared following the manufacture's protocol and 50 μl was added to each sample for 5 min at room temperature to stop the reaction. Fixed samples were subsequently washed with 4°C DPBS supplemented with calcium and magnesium at 450 *g* and 4°C for 5 min. The supernatant was discarded and red blood cells (RBC) were lysed in 2 ml freshly prepared RBC lysis solution (80.2 g NH_4_Cl, 8.4 g NaHCO_3_, 126 ml EDTA [100 mM] at pH 7.4) on ice. Cells were washed again twice in cold supplemented PBS and resuspended in 100 μl filtered 0.5% bovine serum albumin‐PBS (BSA from Sigma‐Aldrich, designated FACS buffer). For immunophenotyping, 2 μl of mouse anti‐human CD14 Pacific Blue™ (1:50), anti‐HLA‐DR APC (1:50), and anti‐human CD2‐PE (1:50), CD15‐PE (1:50), CD19‐PE (1:50), CD56‐PE (1:50), and anti‐NKp46‐PE (1:50) antibody were added and incubated for 20 min on ice. Cells were washed again in FACS buffer, resuspended in 100 μl fresh FACS buffer and fixed with 100 μl medium A, Fix & Perm™ cell permeabilization kit (Thermo Fisher) for 15 min at room temperature. Cells were washed in 2 ml FACS buffer and the supernatant discarded. The pellet was resuspended in 100 μl FACS buffer and 100 μl of medium B was added for permeabilization. 2–3 μl of mouse anti‐human TNF‐FITC (1:33), IL1β‐FITC (1:50), and IL6‐FITC (1:50) was added to each sample with one FMO control, and left for incubation on ice for 20 min. Subsequently, cells were washed in 2 ml cold FACS buffer and then prepared for flow cytometry: cells were resuspended in 350 μl FACS buffer and fixed with 50 μl 4% PFA in PBS, and were kept at 4°C and dark until analysis (within 2 h). For immunophenotyping of classical and non‐classical monocytes by flow cytometry, we used a gating strategy described previously (Belge *et al*, 2002). The FSC‐A threshold was set to 5,000.

#### Determination of leukocyte activation in human whole blood

As described previously (Kiefer *et al*, 2021), 100 μl human whole blood samples were incubated with 25 μg/ml pCRP or mCRP, C10M and 20 μM ADP, respectively, or stayed unstimulated for 90 min at 37°C, 5% CO2. CellFIX™ cell fixation solution at 21°C was added as described above to each sample for 5 min at room temperature to stop the reaction. Fixed samples were subsequently washed with 4°C DPBS supplemented with calcium and magnesium at 450 *g* and 4°C for 5 min. The supernatant was discarded and RBCs were lysed in 2 ml freshly prepared RBC lysis solution at 4°C for 5–10 min. Cells were washed again twice in cold supplemented PBS and resuspended in 100 μl filtered FACS buffer. 2 μl of mouse anti‐human CD14‐Pacific Blue™ (1:50), anti‐human CD16‐PE‐Cy7 (1:50), anti‐HLA‐DR APC (1:50), and anti‐human CD2‐PE (1:50), CD15‐PE (1:50), CD19‐PE (1:50), CD56‐PE (1:50), and anti‐NKp46‐PE (1:50) antibody and 5 μl of anti‐human CD11b‐FITC (1:20) antibody were added and incubated for 20 min on ice. Subsequently, cells were washed in 2 ml cold FACS buffer and then prepared for flow cytometry as described above. Cells were kept in cool and dark conditions until analysis within 1 h. The FSC‐A threshold was set to 5,000.

#### Dissociation of pCRP on ADP‐stimulated platelets and analysis of pro‐inflammatory cytokine expression

Cells were treated as described above with minor modifications. 50 μg/ml pCRP was added with or without compound C10M (molar ratio pCRP:C10M, 1:100–10,000) to adenosine 5′‐diphosphate (ADP, Sigma‐Aldrich) stimulated whole blood to simulate the physiological process of pCRP dissociation *in vitro* (Eisenhardt *et al*, 2009a). 10 μl of ADP in supplemented DPBS were added to 100 μl human whole blood (end concentration 10 and 20 μM ADP) for 30 min at 37°C, 5% CO_2_. Unstimulated controls were not treated with ADP. pCRP and C10M, respectively, were added and whole blood samples incubated again at 37°C, 5% CO_2_. After 6 h, the stimulation was stopped by 50 μl CellFIX™ solution and analysis proceeded as described above. For confocal fluorescence microscopy, cells were treated as described above without immunophenotypic staining. Cell solutions were transferred into tissue culture‐treated polymer μ‐slides (ibidi® μ‐slides VI 0.4 channel slides, ibidi GmbH, Planegg, Germany) and incubated for 1 h at 37°C, 5% CO_2_. Subsequently, cells were washed thoroughly with supplemented DPBS to remove debris and adherent cells were fixed with 4% PFA in PBS and embedded in mounting medium containing DAPI (ibidi GmbH, Planegg, Germany). Slides were analyzed by confocal fluorescence microscopy using a Zeiss LSM 710 and LSM 980, Carl Zeiss Microscopy GmbH, in 3D reconstructions of multiple focal planes (Z‐stack) at 20× magnification, with pinhole diameter of 1 airy unit. DAPI was excited at 405 nm, and fluorescein at 488 nm. Images were processed using IMARIS image visualization and analysis software (Oxford Instruments).

#### HUVEC interaction with platelet‐bound CRP

Activated platelets transform pCRP into pCRP* and mCRP (Eisenhardt *et al*, 2009a; Braig *et al*, 2017), resulting in pro‐inflammatory properties. Hence, the inhibitory compound C10M was tested in a static assay using activated platelets on endothelial cells. Cryopreserved HUVECs were purchased from PromoCell, Heidelberg, Germany. Cells were handled as described by the distributor. For subsequent experiments, no cell passages higher than passage 5 were used. In brief, cells were thawed, transferred to 15 ml Falcon tubes, and washed in DPBS (centrifugation step 200 *g*, 4°C, 5 min) to remove DMSO from cryopreservation. The cell pellet was resuspended in supplemented Endothelial Cell Basal Medium (ECGM, SupplementMix by PromoCell, 10% FCS, 50 U/ml penicillin and 50 μg/ml streptomycin), counted and transferred to T‐75 cell culture flasks. HUVECs were incubated at 37°C, 5% CO_2_ and ECGM was replaced every 2–3 days. Upon 70–90% confluency, cells were split and transferred to appropriate plates for further analysis. For analysis of ICAM‐1 and VCAM‐1 expression analysis by flow cytometry, confluent HUVECs in six‐well plates were serum starved for 4 h and then incubated with 50 μg/ml pCRP with or without C10M and ADP‐activated platelets in 20% normal human serum in ECGM for 6 h as described previously (Braig *et al*, 2017). Cells were detached with trypsin–EDTA solution (1:10 0.5% trypsin–EDTA, Thermo Fisher), washed in 50% fetal calf serum in DPBS, and pelleted at 300 *g*, 4°C for 5 min. The supernatant was discarded, and the cells were resuspended in FACS buffer and stained with anti‐CD54‐PE (1:50) and anti‐CD106‐APC (1:50) antibody on ice for 20 min. Cells were then washed again and fixed with 4% PFA in DPBS and analyzed by flow cytometry within 2 h.

For analysis by confocal immunofluorescence microscopy, HUVEC mono cell layers were cultured in μ‐slides till confluent “cobblestone” appearance. Platelets were isolated from sodium‐citrate whole blood as described above. Pelleted platelets were resuspended in sequestrene buffer and stained with anti‐CD62P‐FITC antibody (1:5) for 15 min at room temperature. Subsequently, calcium was supplemented by adding 13 mM CaCl_2_. Platelet activation was induced by 20 μM ADP and confirmed by P‐selectin expression in flow cytometry (Appendix S1). Stained platelets were washed, counted, and resuspended in HEPES medium, supplemented with CaCl_2_. 100 μl of the solution was incubated with 50 μg/ml pCRP‐Atto 594 with or without C10M (1:100 molar ratio, pCRP to C10M) for 15 min at 37°C, 5% CO_2_. Confluent monolayers of first to third passage were used for experiments. Anti‐CD62P‐FITC antibody (1:5) was used to detect platelets and pCRP‐Atto 594 was used to detect pCRP depositions. Platelet solutions were subsequently added to HUVEC monolayers and incubated for 30 min at 37°C, 5% CO_2_, then monolayers were washed with DPBS supplemented with calcium and fixed in 4% PFA in DPBS for 5 min. HUVEC nuclei were counterstained with DAPI in the mounting medium. Image acquisition was performed by confocal immunofluorescence microscopy (Zeiss LSM 710) and processed with IMARIS (Oxford Instruments). Magnification of the objective was ×20 and ×63 oil, respectively, with pinhole diameter of 1 airy unit. DAPI was excited at 405 nm, fluorescein complex at 488 nm, and Atto 594 at 561 nm.

#### Microvesicle isolation and testing on HUVEC by flow cytometry and confocal fluorescence microscopy

The THP‐1 cell line (DKMZ, Braunschweig, Germany) was used for the generation of microvesicles according to a previously published protocol with the following minor modifications (Braig *et al*, 2017). THP‐1 cells were treated with 10 μg/ml LPS (*E. coli* serotype O127:B8, Sigma‐Aldrich, St. Louis, MO) for 4 h in filtered RPMI 1640, 10% FCS. A subsequent differential centrifugation of the cell suspension was conducted: the cell suspension was centrifuged for 15 min at 500 *g* and room temperature. The cell pellet was discarded and the supernatant containing the microvesicles transferred and centrifuged for 15 min at 1,500 *g* to pellet cellular debris. The supernatant containing microvesicles was collected again and subsequently centrifuged at 20,000 *g* for 40 min at 4°C to pellet the microvesicles. The supernatant was discarded and the designated microvesicle pellet was resuspended in cold PBS supplemented with 0.9 mM CaCl_2_ and 0.49 mM MgCl_2_ and washed twice at 20,000 *g* and 4°C. The total protein concentration was determined using a benchtop fluorometer (Qubit® 3.0 Fluorometer, Invitrogen™ by life technologies™, Carlsbad, CA, USA) and stored until usage at −80°C. HUVECs were treated as described above. For confocal fluorescence microscopy, THP‐1 cells were labeled with CMFDA (Life Technologies) according to the manufacturer's instructions before microvesicle release and purification. CMFDA‐labeled microvesicles were treated as described above. Confluent HUVECs were then serum‐starved for 4 h and then incubated with microvesicles (25 μg/ml of total protein), pCRP (50 μg/ml), pCRP+C10M (1:100 molar ratio, pCRP to C10M) for 6 h at 37°C and processed as described above.

#### Static assay for leukocyte–endothelial interaction

To answer whether the reduction in ICAM‐1 and VCAM‐1 expression is sufficient to inhibit leukocyte binding, leukocyte interaction with HUVEC was assessed as described before with minor modifications (Mangan *et al*, 2007). In brief, HUVECs (HUVEC‐001F‐M, AllCells, LLC) were grown in EGM™‐2 Endothelial Cell Growth Medium‐2 BulletKit™, 2% serum (Catalog #: CC‐3162, Lonza, Switzerland) till confluency and maximal passage 6. For leukocyte interaction assays, cells were subcultured in Falcon® 24‐well clear flat bottom cell culture plates (REF353047) and grown till confluency and cobblestone appearance.

THP‐1 cells cultured as described above and primary human neutrophils isolated from human whole blood as described above, respectively, were stained using the lipophilic cell membrane stain 1,1′‐Dioctadecyl‐3,3,3′,3′‐Tetramethylindocarbocyanine Perchlorate (DiI; Invitrogen™ Thermo fisher) at 4.3 μM (4 μg/ml) for 10 min at 37°C and 5% CO_2_ as described before (Spötl *et al*, 1995). Excessive DiI stain was removed by three washes with RPMI medium at 600 *g*, 7 min at room temperature. Cells were allowed to rest for 90 min before transferring to the HUVEC monolayer. Human neutrophils were isolated from fresh heparinized peripheral whole blood as described above. The isolated neutrophils were stained with 4.3 μM DiI for 10 min at 37°C and 5% CO_2_.

HUVECs were stimulated for 6 h before leukocytes (5 × 10^6^/monolayer and well) were added. A positive control was stimulated with TNF (50 U/ml). This concentration was described to be sub‐maximal (Mangan *et al*, 2007). 24‐well plates with leukocytes and HUVEC monolayer incubated for 30 min and shaking at 75 rpm and 37°C in a shaking incubator (SI4‐2, Sheldon Laboratory Systems, Cornelius, OR, USA) as described before (Mangan *et al*, 2007). Subsequently, the monolayer was washed with warmed PBS three times and adherent cells were fixed with 4% PFA.

For quantification, five non‐overlapping ROI per well at 10× magnification were taken using an Olympus IX71 inverted widefield microscope. Leukocytes were counted automatically in the DiI channel (excitation 550 nm, emission 568 nm) using ImageJ (Schneider *et al*, 2012) as described before (Grishagin, 2015): Images were converted to greyscale, threshold was adjusted to > 95%, and noise reduced (“despeckled” mode). In binary mode, “watershed” mode divided close cells into individual cells, and automated counting was performed afterwards. A mean was calculated for each well and the experiment was performed in five individual experiments (*n* = 5) for both leukocyte subtypes.

#### Formation of platelet‐bound CRP aggregates with human whole blood leukocytes

Heparinized human whole blood was incubated with autologous isolated platelets. Platelets incubated in serum supplemented with 50 μg/ml pCRP‐Atto 594 with and without C10M (1:100 molar ratio) were added to whole blood samples from the same healthy donor. After 30 min incubation at 37°C, 5% CO_2_ all whole blood samples were fixed with CellFIX™ and processed for confocal fluorescence microscopy as described above. Formation of platelet–monocyte aggregates were analyzed by flow cytometry. Heparinized human whole blood was incubated with either 50 μg/ml pCRP or mCRP. After the given time points, whole blood samples were fixed and processed as described above. For immunophenotyping, cells were stained with anti‐human CD14‐Pacific Blue™ (1:50) and CD62P‐PE (1:5) antibodies. Cell suspensions were counted on low flow rates and complexes positive for CD14 and CD62P were assumed to be platelet–monocyte aggregates.

#### ICR for ROS in human whole blood

ROS generation in CRP stimulated human whole blood was assessed by flow cytometry. DHE was used as a redox indicator for intracellular superoxide production (excitation_max_ 480 nm, emission_max_ 586 nm; Zhao *et al*, 2003; Kalyanaraman *et al*, 2012). Whole blood samples were treated as described above. Freshly taken blood was aliquoted into 100 μl samples and stimulated as described under “Dissociation of pCRP on ADP‐stimulated platelets and analysis of pro‐inflammatory cytokine expression,” with the incubation reduced to 3 h. 10 μg/ml DHE was added to each sample in the last 30 min with one FMO‐control untreated. Anti‐human CD14‐Pacific Blue™ (1:50), CD16‐PE‐Cy7 (1:50), and HLA‐DR‐APC (1:50) antibodies were added, resuspended in 400 μl FACS buffer, and analyzed by flow cytometry within 30 min. For all ROS assays, the FSC‐A threshold was set to 35,000. ROS generation in neutrophils and monocytes was differentiated by immunophenotyping and determined by DHE mean fluorescence intensity.

#### Liposome‐binding studies

The binding of pCRP to exposed PC head groups of perturbed membranes can be mimicked by liposomes including LPC in the lipid bilayer (Volanakis & Wirtz, 1979). Liposomes were produced as described before by our group (Braig *et al*, 2017). In brief, egg‐LPC and egg‐phosphatidylcholine were purchased from Avanti Polar Lipids (Alabaster, AL, USA). The lipids were dissolved in chloroform and methanol, 3:1 v/v and mixed in a molar ratio of 4:1 (PC:LPC). The resulting emulsion was dried and evaporated by vacuum. Lipids were then rehydrated in PBS and sonicated using a 3.2 mm microtip probe sonicator (Misonix S‐4000, Qsonica; 20 kHz operating frequency, 1 W sonication power at 10% amplitude, with 30 s ON/ 10 s OFF cycles and 10 cycles). The size of the resulting liposomes was checked using dynamic light scattering (Zetasizer Nano ZEN 3600, Malvern Pananalytical). The liposomes were stored at 4°C until use.

#### Quantitative assessment of NET formation in pCRP*/mCRP stimulated isolated human neutrophils by confocal microscopy

Neutrophils were isolated from human whole blood by density gradient separation method as described by Böyum (1968) and modified by Ferrante & Thong (1980) with minor modifications. In brief, neutrophils were isolated from freshly drawn human whole blood and anticoagulated with heparin as described above. The blood was layered over Histopaque 1077 and Histopaque 1119 (Sigma‐Aldrich, St. Louis, MO), and centrifuged for 30 min and room temperature at 600 *g* with brakes off. Cells were collected from the appropriate layer, diluted in Dulbecco's modified eagle medium (DMEM) supplemented with 10% heat‐inactivated serum (DMEM‐HIS). Isolation and culture was performed in the presence of autologous human serum to prevent spontaneous formation of NETs as described for serum‐free culture conditions (Kamoshida *et al*, 2017). The resulting cell solution was layered on Histopaque 1119 and centrifuged again to minimize RBC contamination. The final cell suspension was free of RBCs and was adjusted in DMEM to 10^6^ cells per ml. 8‐well on cover glass II slides (Sarstedt, REF 94.6190.802) were coated using poly‐L‐lysine (P8920, Sigma Aldrich) following the manufacturer's instructions and were used within 2 days. Cells were seeded in each well at 10^5^ cells per well.

Cells were stimulated with pCRP (100 μg/ml), with or without PC:LPC liposomes (LP; 20 μg/ml) and C10M (1:100 molar ratio, pCRP:C10M) for 3 h at 37°C, 5 %CO_2_. Phorbol 12‐myristate 13‐acetate (PMA; 100 nM) served as a positive control. The cell activity was stopped by adding 4% PFA, as PFA at concentrations of 1–4% fix but do not permeate the plasma membrane (Masuda *et al*, 2016).

Cells were then washed and serum blocked (1% BSA in PBS, supplemented with 0.1% Tween‐20). Staining was performed using polyclonal rabbit anti‐histone 3 (1:100; citrulline R2 + R8 + R17; abcam 5103) and polyclonal goat anti‐MPO (1:80; AF3667, R&D Systems) antibodies. Cells were washed again in 1% BSA in PBS‐T, and secondary antibody donkey anti‐goat IgG Alexa Fluor® 546 (1:2,000; A‐11056, Thermo Fisher) was added for 30 min at RT. Cells were washed again, and an antibody mix containing goat anti‐rabbit IgG with Alexa Fluor® 647 (1:2,000; A‐21245, Thermo Fisher), Hoechst 3342 (1 μg/ml; AB228551, abcam), and Sytox Green (1:10,000) were added for 30 min at RT. Cells were washed again and analyzed within 2 days.

Quantification was performed as described before with minor modifications (Kenny *et al*, 2017). Cells were measured using a Nikon A1r Plus si NIR modified inverted confocal microscope at 40× (CFI Apo LWD Lambda S 40XC WI) with pinhole diameter of 1 Airy Unit. For each well, Z‐stacks from 5 random, non‐overlapping ROIs were collected. The median of the five ROI values was used and the results were statistically analyzed using matched one‐way analysis of variance (ANOVA) and Tukey *post‐hoc* test. The experiment was conducted as a biological triplicate (*n* = 3).

#### Analysis of CRP‐depended opsono‐phagocytosis in human whole blood

CRP opsonization of pathogens was carried out as described previously (Bharadwaj *et al*, 1999; Mold *et al*, 2001). Here, we investigated the influence of C10M on phagocytosis in a human whole blood assay.

In brief, heparinized whole blood was taken from healthy human volunteers and processed within 30 min and challenged with *Streptococcus pneumoniae* serotype 27, *Escherichia coli* (XL2) and zymosan (*Saccharomyces cerevisiae*), respectively. Bacteria were heat‐killed before the experiments and each target particle was fluorescence tagged with FITC as described elsewhere (Nuutila & Lilius, 2005). 2.5 μl of zymosan‐FITC (10 mg/ml), 10 μl of *S. pneumoniae*‐FITC and 5 μl of *E. coli*‐FITC (each 10^9^ cells/ml) were added to 100 μl human whole blood for 5, 10, 15, and 20 min.

Cells were then fixed and washed, and RBC lysis performed as described above. For immunophenotyping, cells were incubated with an antibody mix containing anti‐human CD14‐Pacific Blue™ (1:50), CD16‐PE‐Cy7 (1:50), HLA‐DR‐APC (1:50) and anti‐human CD2‐PE (1:50), CD15‐PE (1:50), CD19‐PE (1:50), CD56‐PE (1:50), and NKp 46‐PE (1:50) antibodies for 15 min on ice. Cells were washed and processed as described above and analyzed within 2 h by flow cytometry (BD LSR Fortessa Cell Analyzer). Opsonization of target particles was done as described previously with minor modifications (Mold *et al*, 2001): Target particles were incubated before each experiment with 100 μg/ml pCRP in DPBS supplemented with 0.9 mM CaCl_2_ at 37°C, 5% CO_2_ for 30 min. Where indicated, C10M was added to the mixture in 1:100 molar ratio of pCRP:C10M. Target particles were then washed and added to whole blood. Effects of C10M on phagocytosis in the absence of CRP were analyzed using equivalent amounts of C10M. We further like to thank Dr. Mark van der Linden, head of National Reference Center for Streptococci, Department of Medical Microbiology, University Hospital (RWTH), Aachen, Germany, for providing *Streptococcus pneumoniae* serotype 27.

#### Animal ethics and experiments

The use of external administered human CRP in various rat models of acute inflammation represents the established standard of both *in vivo* and *ex vivo* studies of the inflammatory profile of human CRP (Gill *et al*, 2004; Pepys *et al*, 2006; Thiele *et al*, 2014; Braig *et al*, 2017).

All surgical procedures and experiments were conducted according to the recommendations of the animal ethics committee of the University of Freiburg Medical Center, Germany. The experimental protocols were approved by the animal ethics committee of the University of Freiburg Medical Center, Germany (35‐9185.81/G‐10/114; 35‐9185.81/G‐13/106; 35‐9185.81/G‐16/53). Animals were cared for by the professional animal keepers of the Center for Experimental Models and Transgenic Service (CEMT), University of Freiburg Medical Center, Germany. Rats were housed in light‐controlled rooms with a 12‐h day/night cycle at 24°C. Food and water were accessible *ad libitum*. Prior to all surgical procedures, the rats were kept in species appropriate animal husbandry. The renal IRI experiments were carried out on male Wistar rats. All rats used in the renal IRI experiments were 6 weeks old and body weight was between 180 and 220 g (Charles River Research Models and Services, Sulzfeld, Germany) as described previously (Thiele *et al*, 2018). An acute rejection model of a hindlimb allograft was performed as originally described by Doi (1979). Male Wistar, Brown‐Norway, and Lewis rats were used in the hindlimb allograft experiments (Charles River Research Models and Services, Sulzfeld, Germany).

#### Ischemic acute kidney injury model in rats

The experimental protocol has been described previously by our group (Thiele *et al*, 2018) and was conducted with minor modifications. In brief, male Wistar rats (6 weeks old, body weight 180–220 g) were anesthetized with 1.5–2 vol % isoflurane (Abbott, Wiesbaden, Germany). Both renal pedicles were dissected via two flank incisions and clamped for 45 min followed by a 24‐h reperfusion period. 25 μg pCRP per ml serum volume was injected intraperitoneally at the end of the ischemia and 12 h later. Rats intravenously received either DPBS (IRI, IRI + pCRP, sham + pCRP) or C10M (IRI + pCRP + C10M, IRI + C10M) in DPBS (1:100 in molar ratio, pCRP to C10M) four times every 6 h starting with the beginning of the reperfusion period. After 24 h, rats were killed and tissue prepared as described previously (Thiele *et al*, 2018). Renal excretory function in acute ischemic kidney injury was assessed as described previously by blood urea nitrogen (BUN) concentration in serum (Thiele *et al*, 2018). Thus, blood samples were taken at given time points from the tail vein into micro tubes with clotting activator (Micro tube 1.3 ml Z, Clotting Activator/Serum, Sarstedt) and centrifugated after clotting. BUN was measured using a Cobas 8000 modular analyzer (Roche, Basel). Hemolytic samples were discarded.

#### Immunostaining and histomorphological evaluation

Immunohistochemistry and histomorphological evaluation of the renal tissue was performed on formalin‐fixed paraffin‐embedded renal tissue sections (5 μm thick serial sections). Paraffin‐embedded sections were de‐paraffinized in xylol, rehydrated, and boiled for 20 min in concentrated citric acid (pH 6.0). Antigen unmasking for anti‐monocyte detection was done by application of pepsin solution (Digest‐All™ 3, life technologies) at room temperature for 20 min. Previously, both kidneys were flushed with DPBS followed by fixation in 4% PFA. Evaluation was performed as described previously (Thiele *et al*, 2018): histomorphological changes were evaluated in a blinded fashion by two researchers using a Zeiss microscope (Carl Zeiss Microscopy Axio Imager.M2, Germany) on Periodic acid–Schiff stained sections by quantitative measurement of tubulointerstitial injury, which was assessed by loss of tubular brush border and cast formation. The morphological assessment was scaled in five steps: not present (0), mild (1), moderate (2), severe (3), and to very severe (4). Transmigrated leukocytes were detected by anti‐rat CD68 antibody (clone ED‐1) in a 1:100 dilution and renal inflammation was evaluated by counting ED‐1 positive cells in 20 randomized areas of interest of the renal cortex at ×200 magnification. Sections were counterstained with Mayer's hematoxylin. Unspecific isotype matched primary antibodies served as negative control. Detection of human CRP on the renal tissue sections was performed using anti‐pCRP*/mCRP antibody 9C9 (1:10 dilution).

#### Hindlimb transplant rejection model

The two inbred stains Brown‐Norway (BN, recipient) and Lewis (Lew, donor) show a strong antigenic mismatch (Fealy *et al*, 1994) and were used for the acute rejection model. The method was first described by Doi (1979) and was performed with minor modifications. In brief, two experimenters performed transplantation together: while one is working on the donor rat, another was preparing the recipient rat. In both rats, hindlimbs are shaved and thoroughly disinfected, then a circumferential skin incision was performed at mid‐thigh level. The donor limb is first fixed by femoral bone osteosynthesis, which was achieved using an intramedullary rod made from a sterile 0.8 mm Kirschner wire. Muscles are then sutured with 4/0 nylon running sutures with adaption of the according functional groups (thigh extensors, adductors, gluteal muscles, and hamstrings). This model of acute rejection was set as a non‐functional hindlimb transplant, so no suturing of the nerves was performed. Revascularization was performed using 9/0 nylon sutures. Both vessels were sutured under the microscope with 6–8 single stiches. The inguinal fat flap (containing the superficial epigastric artery) from the donor hindlimb is used to cover the anastomoses to prevent major bleeding. The wounds were rinsed with 0.9% saline solution and the transplantation is completed by a Penrose drain including skin closure with running sutures (4/0 nylon). The skin is cleaned with non‐alcoholic disinfectant (Octenisept®, Schülke, Germany) after the skin suture is completed. The total operative time was on average 90 min. All rats received postoperative subcutaneous injections of 100 μg/100 g bodyweight of carprofen for pain relief and 1 ml/100 g bodyweight saline solution for volume compensation. A plastic collar was used to prevent auto‐mutilation and hindlimbs with self‐inflicted wounds were excluded from further evaluation. For postoperative management after the completion of the skin suture, sufficient reperfusion of the transplanted hindlimb was assessed again. Rats then received a first intraperitoneal bolus of 25 μg pCRP per ml serum volume and 500 μl of DPBS supplemented with calcium and magnesium (control), respectively. The second bolus was administered 24 h after the first. An intravenous catheter (Abbocath‐T 26G, 0.6 × 19 mm) in the tail vein was used to inject C10M (1:100 molar ratio to pCRP) in DPBS and DPBS, respectively, every 6 h for the first 42 h (eight applications in total, starting with the first intraperitoneal bolus; Fig 6A). Rats are allowed to awake from anesthesia and cared for until fully awake and warmed. All rats showed slight edema of the transplanted hindlimb within the first postoperative day. Rejection of the hindlimb graft was assessed by clinical control every 8 h and graded as described previously (Jindal *et al*, 2015) according to an established clinical classification for allograft rejection, from 0 (no clinical signs of rejection), 1 (edema), 2 (erythema), and 3 (epidermolysis and desquamation) to 4 (necrosis). Four experimental groups were included in this study (*n* = 4). In the control group (*n* = 4, Lew→BN), Brown‐Norway recipient rats received intraperitoneal DPBS administration. Rats in the pCRP group received two intraperitoneal boli of 25 μg pCRP (BD Micro‐Fine™ + Demi, 30G insulin syringes) per ml serum volume directly following to the surgical procedure and after 24 h. Serum volume was estimated as described previously as a function of the body weight (Thiele *et al*, 2012). Immediately after surgery, subcutaneous saline supplementation was given to avoid dehydration of the rats. In the C10M treatment group (*n* = 4, Lew→BN), rats were treated as in the pCRP group. Additionally, rats received intravenous compound C10M (1:100 molar ratio) via a 26G catheter (Abbocath‐ T, ICU Medical B.V., Netherlands) in the lateral tail vein every 6 h for the first two postoperative days. Biopsies were taken on day three after transplantation of skin and muscle tissue and immunohistochemistry performed on formalin‐fixed and paraffin‐embedded samples. After incubation with primary antibody anti‐CD68 (clone ED1, 1:100) and anti‐human CRP (clone CRP‐8, 1:200) for 1 h at room temperature, slides were incubated with secondary antibody anti‐mouse‐conjugated CF488 (green) following the manufacturer's protocol.

#### Pharmacokinetic studies in rats

Plasma concentrations of C10M were measured by an LC–MS method after a single intravenous injection into the tail vein of male Wistar rats (250–350 g) (Kather *et al*, 2021). Rats were anesthetized as described above and temperature controlled. 100 μg of C10M was injected and blood samples were taken at given time points (1, 5, 10, 15, 30, 45, 60, and 90 min after bolus injection). EDTA‐anticoagulated (1.6 mg/ml EDTA) blood samples were centrifugated for 10 min at 2,000 *g* and 4°C to remove the cellular portion. The resulting supernatant was snap frozen and stored at −80°C until further sample preparation with solid‐phase extraction.

Renal excretion of C10M was measured by a model previously described (Yaksh *et al*, 1986; Kather *et al*, 2021) with marginal modifications. Male Wistar rats (300–350 g bodyweight) were anesthetized as described above and the urinary bladder was carefully exposed and externalized under sterile conditions. Sterile urine was drawn from the bladder after a single intravenous application of C10M and C10M + pCRP 15, 30, 45, 60, and 90 min after the i.v. application, respectively. The urine samples were immediately snap frozen and stored at −80°C until measurements taken. Pharmacokinetic parameters were calculated using PKSolver add‐in for Microsoft Excel (Zhang *et al*, 2010).

#### SDS–PAGE and Western blotting

For SDS–PAGE, tissue lysates from rat kidneys, muscle and skin tissue samples were precipitated on ice with same volumes of 10% trichloroacetic acid after homogenization with a disperser tool on ice (Ultra Turrax^®^ IKA^®^, Germany). Pelleted protein was denaturated in SDS loading dye supplemented with DTT at 95°C, 5 min and then separated on 10–12% SDS‐polyacrylamide gels. After Western blot, nitrocellulose membranes were blocked in 5% BSA in TBS‐T and incubated with mouse anti‐human CRP antibody (clone CRP‐8, 1:2,000). HRP‐conjugated sheep anti‐mouse antibody 1:5,000 v/v in 1% BSA‐TBS‐T was used to detected bound CRP antibodies after washing steps. GAPDH served as loading control and was detected with anti‐GAPDH‐HRP (1:1,000 in 1% BSA in TBS‐T). Protein bands were visualized using ECLTM Western blotting analysis system (GE Healthcare, UK), medical x‐ray film (Fujifilm, Japan), and developed on a CURIX 60 developer (AGFA).

For detection of CRP deposits in IRI kidneys, snap frozen tissue was homogenized on ice using a high‐power disperser in lysis buffer with added protease inhibitors. After centrifugation of the homogenized tissue, the supernatant was transferred, and protein concentrations were determined with BCA protein assay, and processed as described above.

For semiquantitative analysis of CRP binding to activated human platelets, we performed SDS–PAGE and Western blotting as described previously (Eisenhardt *et al*, 2009a). Briefly, human platelets were isolated and washed from citrate‐anticoagulated whole blood by differential centrifugation in sequestrene buffer. pCRP (100 μg/ml) was incubated with ADP‐activated platelets and C10M at different concentrations (10 and 100 mM, Fig 2C showing 10 mM). Calcium‐depleted platelets served as a control. Platelets were then washed three times in DPBS supplemented with calcium. Platelets were pelleted, resuspended in 10 mM HEPES buffer at pH 7.4, 10 mM KCl, 1.5 mM MgCl_2_, 1 mM EDTA, with added protease inhibitors (10 μl of 200 mM PMSF in DMSO, protease inhibitor cocktail in DMSO, 100 mM Na‐orthovanadate per 1 ml; sc24948 RIPA Lysis Buffer System, Santa Cruz, Biotechnology, Dallas, TX, USA) and homogenized on ice by applying shear‐stress followed by freeze‐and‐thaw cycles in liquid nitrogen. The protein concentration of the lysates was determined by fluorometric assay using a Qubit fluorometer. After the separation by SDS gel electrophoresis and the transfer to nitrocellulose membranes (Hybond ECL, GE Healthcare, Munich, Germany), samples were probed with anti‐CRP antibody (clone CRP‐8; 1:2,000) overnight at 4°C. Monoclonal antibodies against GAPDH (1:1,000) were used to ensure protein equilibration. Secondary HRP‐conjugated anti‐mouse (1:5,000) antibodies and enhanced chemiluminescence (ECL, GE Healthcare) were used to detect protein signals and were conserved on FUJI Medical X‐Ray Film (FUJIFILM, Japan).

#### Statistical analyses

All statistical analyses were performed using GraphPad Prism v9.0 for Mac (GraphPad Software, La Jolla, CA, USA). Experiments were performed at least three times with precise statements for each experiment given. The data are shown as mean and standard error of the mean (mean ± SEM) as indicated. One‐way ANOVA and *post‐hoc* Tukey's test were used to compare more than two groups, with Gaussian distribution tested previously. If only two groups were compared, a two‐tailed Student's *t*‐test was employed. A *P* value of < 0.05 was considered statistically significant. For the hind limb allograft transplantation model, Kaplan–Meier curves for different treatments were compared using log‐rank test. Hindlimb survival interval was then compared by Mantel–Cox log‐rank test. Median survival is given for all groups.

**The paper explained**

1 **Problem**

CRP is the prototypic acute phase reactant, established as a marker of inflammation in general and in particular, a predictor of cardiovascular risk. Recent evidence indicates the role of CRP as a direct pathogenic pro‐inflammatory mediator in cardiovascular diseases and states of inflammation. The homo‐pentamer (pCRP) amplifies tissue injury in a conformation‐specific manner. The CRP system consists of at least two protein conformations with distinct pathophysiological functions. The binding of the native pCRP to activated cell membranes leads to a conformational change, resulting in two highly pro‐inflammatory isoforms, pCRP* and monomeric CRP (mCRP). pCRP*/mCRP was found to strongly aggravate localized tissue injury in various pathological conditions, including—but not limited to—atherosclerosis, thrombosis, myocardial infarction, and stroke. An inhibitor of the pro‐inflammatory effects of CRP would hold promise to be a broadly usable anti‐inflammatory therapeutic approach.

**Results**

A novel low molecular weight tool compound was modeled after the structure of the pCRP ligand PC, and was shown by X‐ray crystallography to bind to the PC‐binding site in pCRP. The low molecular weight inhibitor C10M successfully blocked the initial pCRP binding to damaged cells and thereby inhibited the formation of the pro‐inflammatory CRP isoforms, that is, pCRP* and mCRP. Thereby, the initiation of the pro‐inflammatory CRP cascade and consequently CRP‐driven inflammation was inhibited both *in vitro* and *in vivo*.

**Impact**

Inhibiting the binding of pCRP to ligands exposed on “activated cell membranes” via the monofunctional low molecular weight tool compound C10M represents a novel, potentially broadly applicable therapeutic approach for many inflammation‐driven diseases. This novel therapeutic approach promises to complement current treatment strategies involving CRP depletion in acute inflammation.

## Author contributions

**Johannes Zeller:** Conceptualization; data curation; formal analysis; supervision; validation; investigation; visualization; methodology; writing—original draft; project administration. **Karen S Cheung Tung Shing:** Investigation; visualization; methodology; writing—review and editing. **Tracy L Nero:** Visualization; methodology; writing—review and editing. **James D McFadyen:** Writing—review and editing. **Guy Krippner:** Visualization; methodology; writing—review and editing. **Balázs Bogner:** Investigation. **Sheena Kreuzaler:** Investigation. **Jurij Kiefer:** Investigation. **Verena K Horner:** Writing—review and editing. **David Braig:** Methodology; writing—review and editing. **Habiba Danish:** Investigation. **Sara Baratchi:** Methodology. **Mark Fricke:** Investigation. **Xiaowei Wang:** Methodology. **Michel G Kather:** Investigation. **Bernd Kammerer:** Methodology. **Kevin J Woollard:** Methodology. **Prerna Sharma:** Investigation. **Craig J Morton:** Investigation; methodology; writing—review and editing. **Geoffrey Pietersz:** Visualization; methodology; writing—review and editing. **Michael W Parker:** Conceptualization; resources; supervision; funding acquisition; writing—review and editing. **Karlheinz Peter:** Conceptualization; resources; supervision; funding acquisition; writing—review and editing. **Steffen U Eisenhardt:** Conceptualization; resources; supervision; funding acquisition; writing—original draft.

## Disclosure and competing interests statement

The authors JZ, TLN, JDM, CJM, GK, GP, MWP, KP, and SUE are inventors on a patent application for a CRP‐specific small‐molecule inhibitor. Therefore, we declare that this work was conducted under circumstances that could be interpreted as a potential conflict of interest as a financial competing interest. The remaining authors have no conflict of interest to declare.

## For more information

*Relevant database*, *Protein data bank* (*PDB*, http://www.wwpdb.org): The PDB coordinates of the ligand phosphocholine (PC) and pCRP, PC:pCRP complex, can be found under PDB ID: 1B09 (Thompson *et al*, 1999).

The PDB coordinates of the compound C10M:pCRP complex have been deposited under PDB ID: 7TBA.

*Corresponding Author's websites*:

Prof Michael W Parker: https://www.bio21.unimelb.edu.au/Michael‐parker.
Prof Karlheinz Peter: https://baker.edu.au/research/laboratories/atherothrombosis‐vascular.
Prof Steffen U Eisenhardt: https://www.uniklinik‐freiburg.de/plastischechirurgie/forschung/ag‐eisenhardt.html.

## Supporting information



Appendix

Expanded View Figures PDF

Table EV1

Table EV2

Source Data for Expanded View

PDF+

Source Data for Figure 1

Source Data for Figure 2

Source Data for Figure 3

Source Data for Figure 4

Source Data for Figure 5

Source Data for Figure 6

Source Data for Figure 7

## Data Availability

The coordinates of compound C10M:pCRP complex have been deposited under PDB ID: 7TBA (https://www.rcsb.org/structure/7TBA). This study includes no additional data deposited in external repositories.
