# Supplementary material for: A novel phosphocholine‐mimetic inhibits a pro‐inflammatory conformational change in C‐reactive protein
Source: EMBO Mol Med. 2022 Dec 5;15(1):e16236. doi: 10.15252/emmm.202216236 (PMC9832874; doi:10.15252/emmm.202216236)
Supplement: Supplementary file 1 — Appendix [file EMMM-15-e16236-s008.pdf]

Table of content

1. ARRIVE guideline information page 1-2
2. Appendix Figure S1 page 3
3. Appendix Figure S2 page 4
4. Multiplex human Cell line Authentication test for THP-1 cell line page 5

ARRIVE GUIDELINE INFORMATION

Study Design: Renal Ischemia/Reperfusion-Injury (IRI) Model

Sample Size: Sample size was calculated based on earlier *in vivo* studies performed in our labs (Thiele et al., Circulation, 2014; Braig et al., Nature Com, 2017). Initial expert statistical advice was given by Dr. M. Olschewski and Prof. D. Hauschke, Institute for Medical Biometry and Medical Informatics, University of Freiburg. For *a priori* sample size calculations, we used the statistic software G*Power (Faul, F., Erdfelder, E., Lang, A.-G., & Buchner, A. (2007). G*Power 3: A flexible statistical power analysis program for the social, behavioral, and biomedical sciences. Behavior Research Methods, 39, 175-191.). The calculations were approved as a part of the Animal Ethics Proposal by the Animal Ethics Committee of University of Freiburg Medical Center (Ethics approval number G13/106).

The analysis method (t-test for two means), the effect size (1.330). For the estimate of variability, we used the number needed to treat in a previously published study from authors of our group as a reference (Thiele, Zeller et al., Frontiers in Immunology). The experiments were conducted under identical conditions to the planned experiment described here. The power (1-β) was set to 80%. The significance threshold α was set at 0.05. Due to long-standing experience with animal experiments in both surgeons performing the experiments, the sample size in each group at the start of the study and the n-numbers in the analysis is the same (n=10). The character of the experiment not allowed for the re-use of animals between experiments.

Inclusion Criteria:

Healthy male Wistar rats, 6-weeks-old and weighing 180-220 g.

Exclusion Criteria:

Unexpected problems during operation (bleeding, injury of the parenchyma); pain as expressed by the Rat Grimace Scale (Sotocinal SG et al. (2011). The Rat Grimace Scale: a partially automated method for quantifying pain in the laboratory rat via facial expressions. Molecular Pain 7: 55.) higher than “not present” after the surgery.

Randomization:

Yes, rats were randomized allocated to a group before surgery. Randomization was performed by simple randomization.

Blinding: Yes, samples were pseudonymized. Allocation to each group was performed in a randomized manner prior to the experiments. Treatment (except for sham) was blinded as we applied pCRP or the vehicle, respectively, and all animals received i.v. applications with or without the compound of interest. *Ex vivo* analysis was performed in a blinded manner except for detection of pCRP in rat tissue by SDS-PAGE and Western blotting.

Groups	Sex	Age	Number (prior to experiment)	Number (after termination)	Littermates (Yes/No)	Other description
**Group 1 Sham**	male	6-weeks-old	10	10	No	Sham-operated rats. No Ischemia/Reperfusion-Injury (IRI) applied.
**Group 2 IRI**	male	6-weeks-old	10	10	No	As in Group 1, but IRI applied.
**Group 3 IRI + pCRP**	male	6-weeks-old	10	10	No	As in Group 2. Human pCRP is added by intraperitoneal application at given time-points.
**Group 4 IRI + pCRP + C10M**	male	6-weeks-old	10	10	No	As in Group 3. C10M is added by intravenous application at given time-points.
**Group 5 IRI + C10M**	male	6-weeks-old	10	10	No	As in Group 2. Human C10M is added by intravenous application at same time-points as in Group 4.
**Group 3 sham + pCRP**	male	6-weeks-old	10	10	No	As in Group 1, but Human pCRP is added by intraperitoneal application at given time-points (same as in Group 3+4)

Study Design: Hindlimb VCA Model

Sample Size: Sample size was calculated based on earlier *in vivo* studies performed in our labs (Thiele et al., Circulation, 2014; Braig et al., Nature Com, 2017). Initial expert statistical advice was given by Dr. M. Olschewski and Prof. D. Hauschke, Institute for Medical Biometry and Medical Informatics, University of Freiburg. For the calculations we used the statistic software G*Power (Faul, F., Erdfelder, E., Lang, A.-G., & Buchner, A. (2007). G*Power 3: A flexible statistical power analysis program for the social, behavioral, and biomedical sciences. Behavior Research Methods, 39, 175-191.)

The calculations were approved as a part of the Animal Ethics Proposal by the Animal Ethics Committee of University of Freiburg Medical Center (Ethics approval number 35-9185.81/G-16/53). The analysis method (t-test for two means), the effect size (1.330). For the estimate of variability, we used the number needed to treat in a previously published study from authors of our group as a reference. The experiments were conducted under identical conditions to the planned experiment described here. The power (1-β) was set to 80%. The significance threshold α was set at 0.05. Due to long-standing experience with animal experiments in both surgeons performing the experiments, the sample size in each group at the start of the study and the n-numbers in the analysis Is the same (n=6). The character of the experiment not allowed for the re-use of animals between experiments.

Inclusion Criteria:

Healthy male Brown-Norway (recipient) and Lewis (donor) rats, 6-week-old and of 200-250 bodyweight.

Exclusion Criteria:

Unexpected problems during operation (bleeding, unusually long ischemia time (> 30 min.)); pain as expressed by the Rat Grimace Scale (Sotocinal SG et al. (2011). The Rat Grimace Scale: a partially automated method for quantifying pain in the laboratory rat via facial expressions. Molecular Pain 7: 55.) higher than "not present" after the surgery; nerves were not coadapted during the replantation and transplantation procedures. Weight loss of > 10% of the initial body weight after transplantation. Auto-mutilation of the transplanted hind limb (rats were wearing protective collars/cones to prevent auto-mutilation).

Randomization:

Yes, rats were randomized allocated to a group before surgery. Randomization was performed by simple randomization.

Blinding: Yes, samples were pseudonymized. Allocation to each group was performed in a randomized manner prior to the experiments. Treatment (except for replantation) was blinded as we applied pCRP or the vehicle, respectively, and all animals received i.v. applications with or without the compound of interest. *Ex vivo* analysis was performed in a blinded manner except for detection of pCRP in rat tissue by SDS-PAGE and Western blotting.

Groups	Sex	Age	Number (prior to experiment)	Number (after termination)	Littermates (Yes/No)	Other description
**Group 1 Replantation**	male	6-weeks-old	10	10	No	Hindlimb replantation in Brown-Norway (BN) rats
**Group 2 Transplantation (Tx)**	male	6-weeks-old	10	10	No	Hindlimb transplantation in fully-mismatched Lewis (Lew, donor) to BN (recipient)
**Group 3 Tx with pCRP**	male	6-weeks-old	10	10	No	As in Group 2. Human pCRP is added by intraperitoneal application at given time-points.
**Group 4 Tx with pCRP + C10M**	male	6-weeks-old	10	10	No	As in Group 3. C10M is added by intravenous application at given time-points.
**Group 4 Tx with C10M**	male	6-weeks-old	10	10	No	As in Group 2. Human C10M is added by intravenous application at same time-points as in Group 4.

Appendix Figure S1

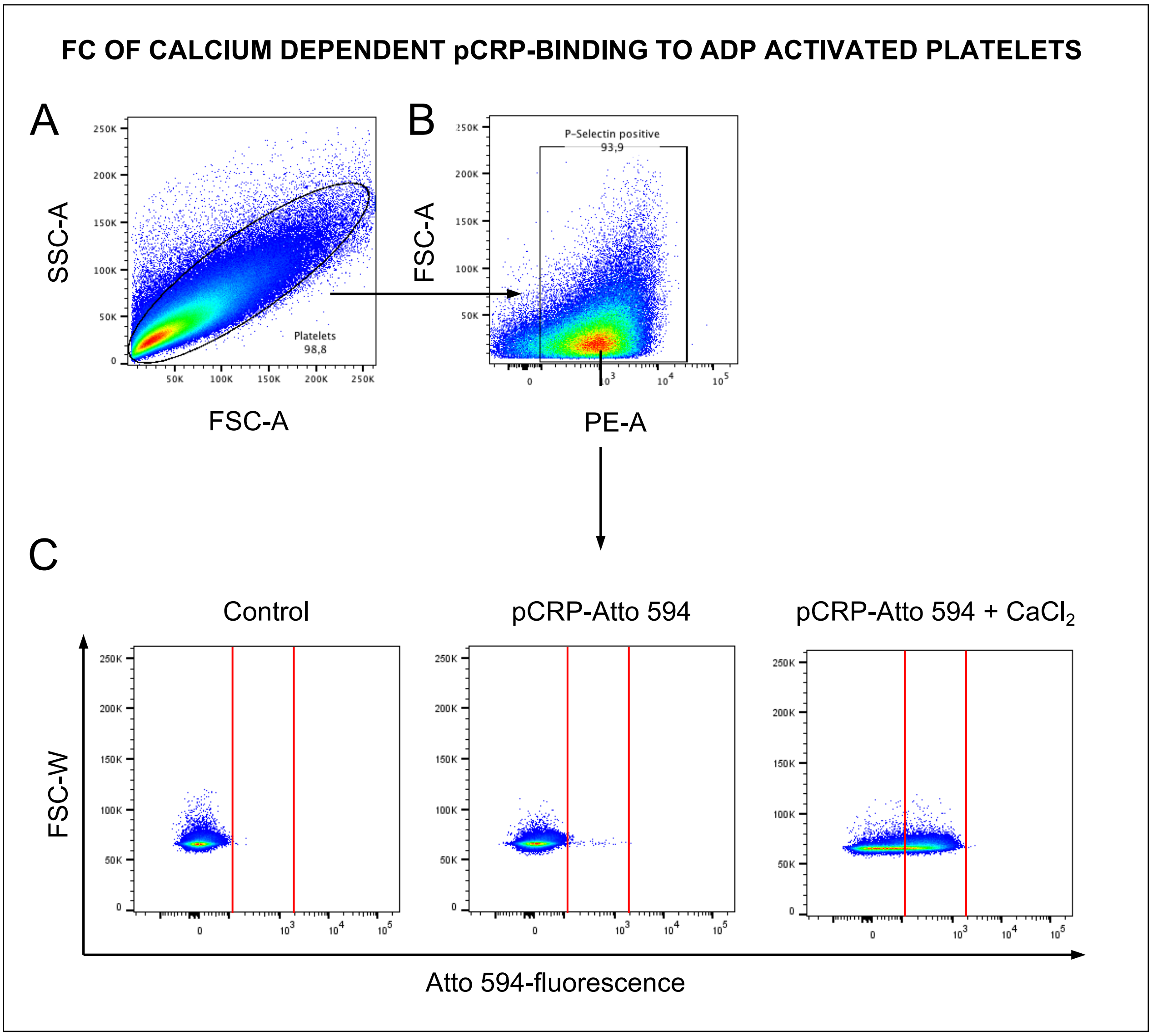

Appendix Figure S1: pCRP-binding to ADP-activated platelets is calcium-dependent. pCRP-Atto 594 incubated with ADP-activated washed platelets showed Ca2+-dependent binding of CRP to P-selectin expressing platelets. The shown gating strategy in flow cytometry (FC) served as control for the inhibition of pCRP-binding to activated platelets by C10M. Washed platelets isolated (A) were gated forward and P-selectin expression (B) was analyzed. P-selectin positive cells were further analyzed for Atto 594 fluorescence (C).

Appendix Figure S2

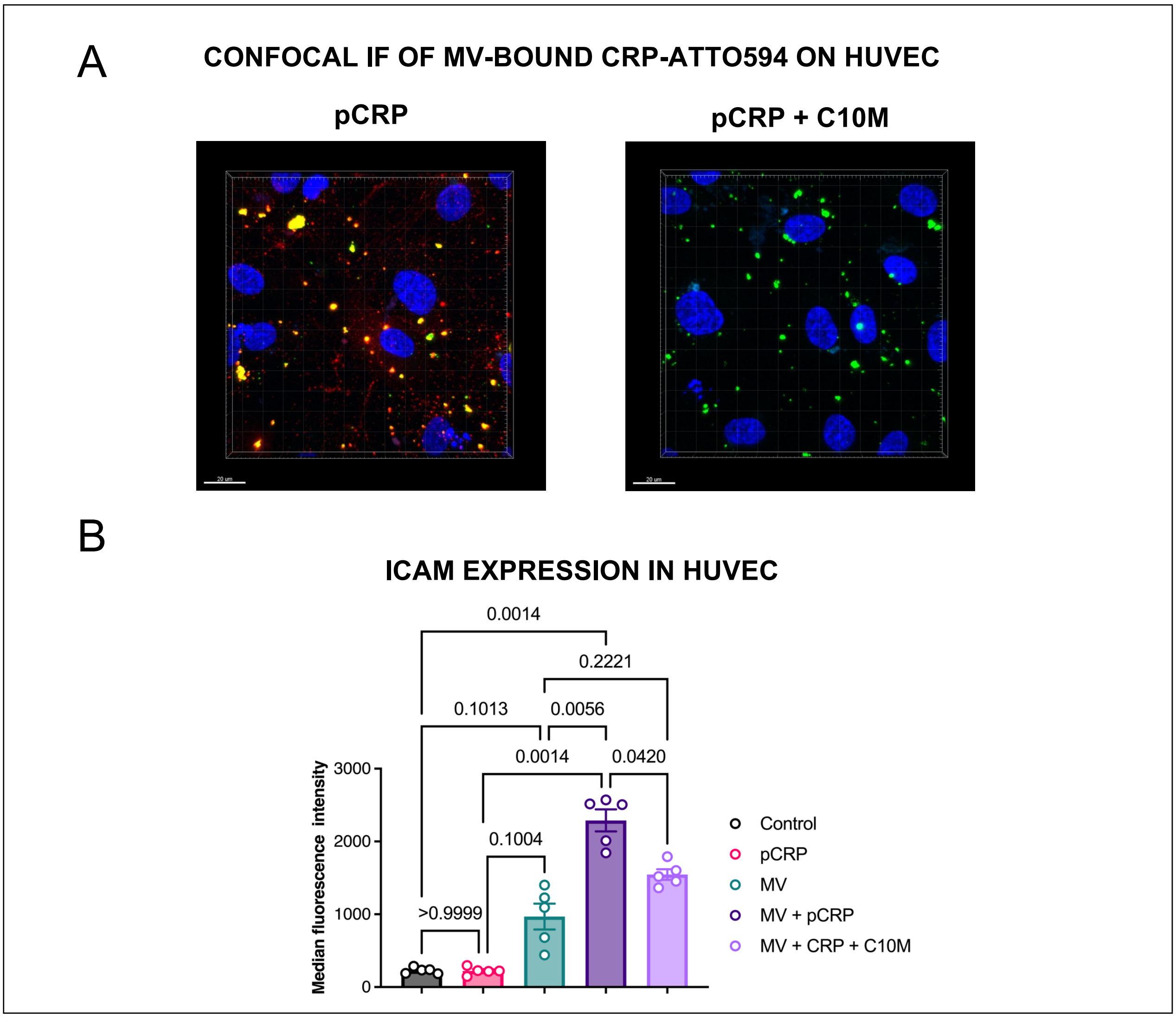

Appendix Figure S2: CRP-binding to microvesicles as a pro-inflammatory vehicle is abrogated by C10M. (A) pCRP-Atto 594 (red) incubated with CMFDA-labeled (green) microvesicles derived from THP-1 cells were found bound to HUVECs (DAPI, blue) by confocal fluorescence microscopy. THP-1 cells were CMFDA-labeled and LPS-stimulated in the presence of pCRP-Atto 594 (50 µg/ml) with and without C10M (1:100 molar ratio, pCRP to C10M). Shedded microvesicles were purified and added to HUVEC monolayers in µ-slides. After 2 hours, cells were fixed and embedded. Scale bar 20 µm. ICAM-1 expression in HUVECs induced by pCRP*/mCRP bound to microvesicles was performed as described in the Methods section of the main text. Isolated microvesicles (MV) treated with pCRP and pCRP+C10M, respectively, incubated with HUVECs for 6 hours. ICAM-1 expression was detected by specific antibody and assessed by flow cytometry. Scatter plots of median fluorescence intensity (MFI) results in flow cytometry are shown with results normalized to control = 100, mean ± SEM. *P* values were calculated with ANOVA (Gaussian distribution assumed, rejected equal SDs) and Dunnett correction. post-hoc test. n=6.

Multiplex human Cell line Authentication test for THP-1 cell line

MULTIPLEXION

Universitätsklinikum Freiburg
David Braig
Zentrale Klinische Forschung, Labor Stark
Breisacher Str. 66
79106

CEO: Dr. Markus Schmitt
Kapellenweg 1
69121 Heidelberg
Fon: (+49) 6221 6174933
Fax: (+49) 6221 424932
E-Mail: info@multiplexion.de
www.multiplexion.com

Human Cell Line Authentication Report

Report ID	157	Order ID	241
Report Date	20.03.2013	Order Date	13.03.2013
		Purchase No.	

Dear David Braig,

Many thanks for your order. The Multiplex human Cell line Authentication Test (MCA) was performed as described at www.multiplexion.de. Please find below the results.

Information from Customer				Results			Summary			
Sample ID	Sample Name	Cell line name	If other: exact name	DNA quality	Best Hit with DataBase	Identity (%)	Present in Database?	Cross-Contamin-ation?	Identity confirmed?	Genotype Code
229	THP-1	THP-1		ok	THP-1	100	no	no	identity confirmed	ATATTTTTTTAWAATTAAAAAAATTT AAATAAAAAAAAAWTTAAAAAT
