# Supplementary material for: A novel phosphocholine‐mimetic inhibits a pro‐inflammatory conformational change in C‐reactive protein
Source: EMBO Mol Med. 2022 Dec 5;15(1):e16236. doi: 10.15252/emmm.202216236 (PMC9832874; doi:10.15252/emmm.202216236)
Supplement: Supplementary file 2 — Expanded View Figures PDF [file EMMM-15-e16236-s005.pdf]

Expanded View Figures

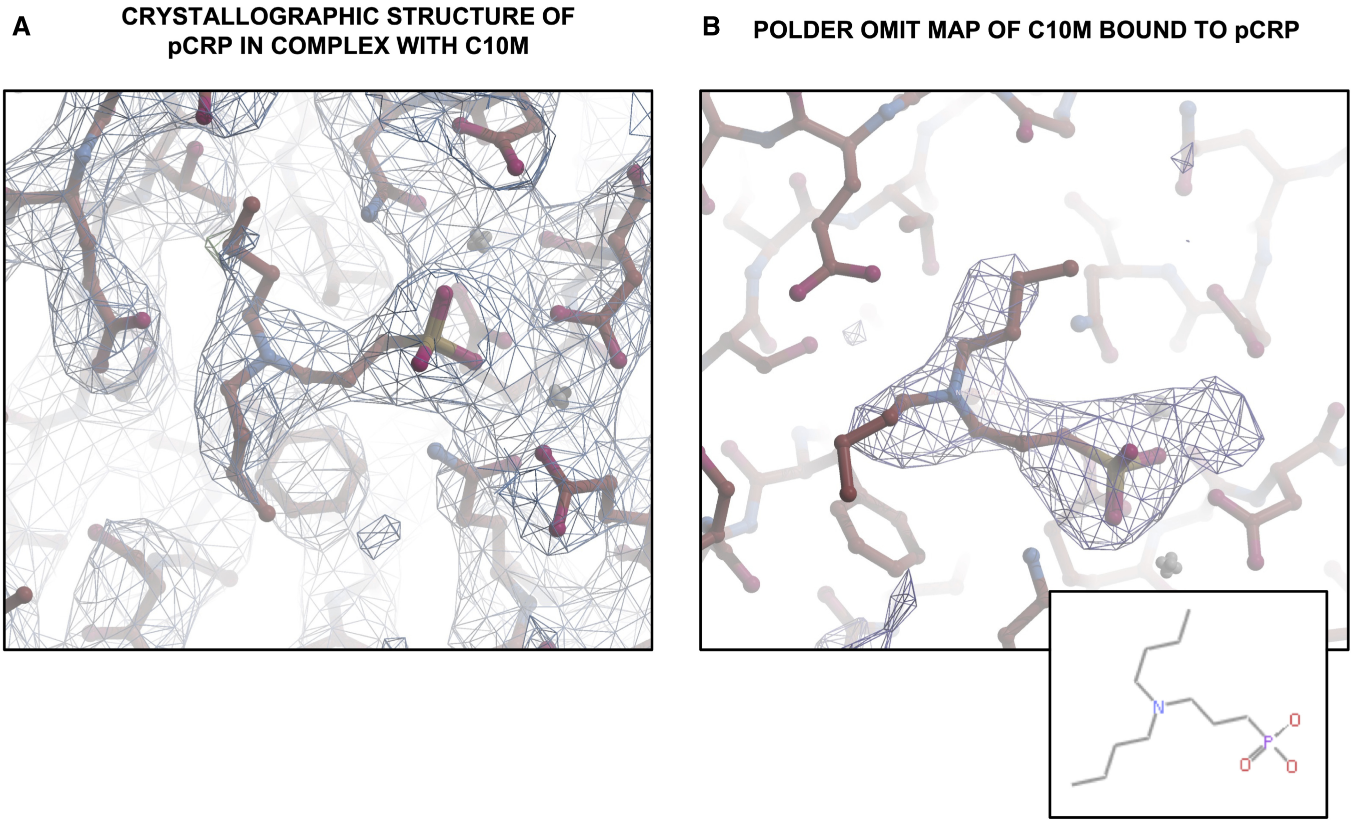

Figure EV1. Crystallographic structure of pCRP in complex with tool compound C10M.

A Crystallographic structure of pCRP in complex with C10M. 2*Fo*-*Fc* electron density map of C10M bound to pCRP (contoured at 1σ).

B Polder omit map (Liebschner *et al*, 2017) of C10M bound to pCRP (contoured at 3σ). The 2D structure of C10M is shown in the inset at the bottom right.

Figure EV2. Inhibitory effects of C10M on pCRP*/mCRP-induced NETosis and leukocyte–endothelial interaction.

A Representative confocal immunofluorescence images of isolated human neutrophils as summarized in Fig 4H. Cells were stained with anti-dsDNA dyes Hoechst 33342 and Sytox Green, and anti-MPO and anti-citH3 antibodies. Shown are uncropped images of merged channels and at 40× magnification. Scale bars indicate 50 μm.

B Representative single-channel confocal immunofluorescence images of pCRP*/mCRP-induced NETosis. Cells demonstrating NETs in the pCRP*/mCRP-stimulated group showed disrupted cell membranes as visible in the transmitted differential and merged channel (black arrow heads), indicating a suicidal mode of NETosis. Scale bars indicate 50 μm.

C–E Leukocyte adhesion on endothelial cells. HUVEC monolayers treated with different isoforms of CRP as described for Fig 4B and C were incubated with fluorescently-labeled THP-1 cells (C, E) and neutrophils isolated from human whole blood (PMN, D) for 30 min. After incubation, the monolayer was washed and adherent cells were fixed. THP-1 cell and neutrophil binding to HUVEC monolayers were then evaluated by automated cell counting in five non-overlapping ROIs at 10× magnification as demonstrated for THP-1 in (E). Scale bars indicate 100 μm. Graph shows mean ± SEM. *P* values were calculated with ANOVA and Tukey's *post-hoc* test. Biological replicates, $n = 5$ for THP-1 and PMN, respectively.

Source data are available online for this figure.

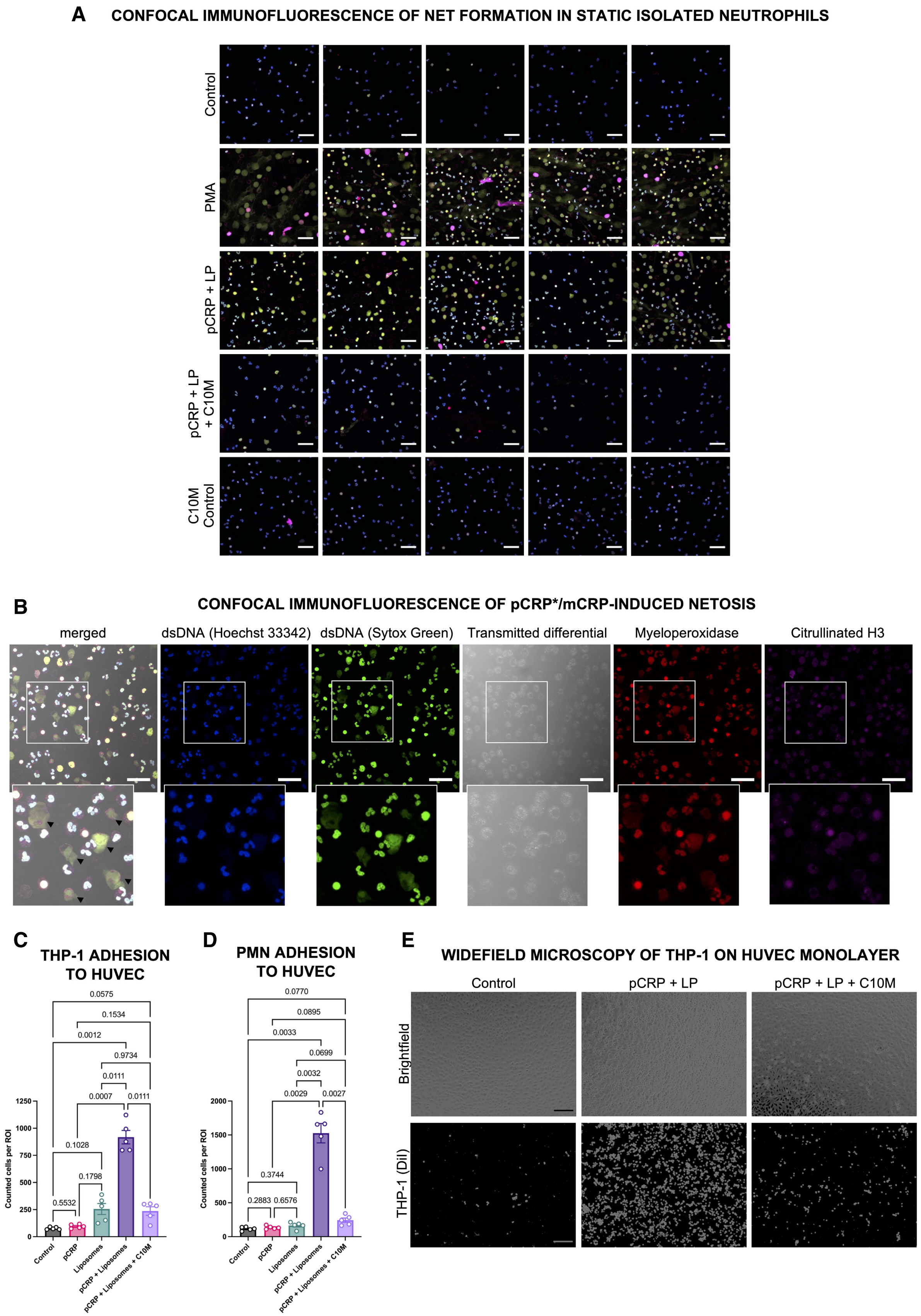

Figure EV2.

© 2022 The Authors

Figure EV3. Flow cytometry-based analysis of phagocytosis of *S. pneumoniae*, *E. coli*, and zymosan in the presence and the absence of C10M.

A, B Phagocytosis of heat-killed and FITC-labeled *S. pneumoniae* by monocytes (A) and neutrophils (B) was analyzed with or without C10M (in equal concentrations used as demonstrated in Fig 7). Bar chart shows phagocytic index (percentage of target positive cells of subtype/all cells of subtype) of an untreated control versus C10M treated cells after 5, 10, 15, and 20 min, respectively. Mean ± SEM are indicated.

C, D Phagocytosis assay for heat-killed and FITC-labeled *E. coli* by monocytes (C) and neutrophils (D).

E, F Phagocytosis of the yeast cell wall ligand zymosan (*Saccharomyces cerevisiae*) by monocytes (E) and neutrophils (F). Mean ± SEM are indicated.

Data information: Statistical analysis for all assays shown was performed using multiple matched *t*-tests. Biological replicates, $n = 3$ and 6. Precise *P*-values are given. Source data are available online for this figure.

© 2022 The Authors

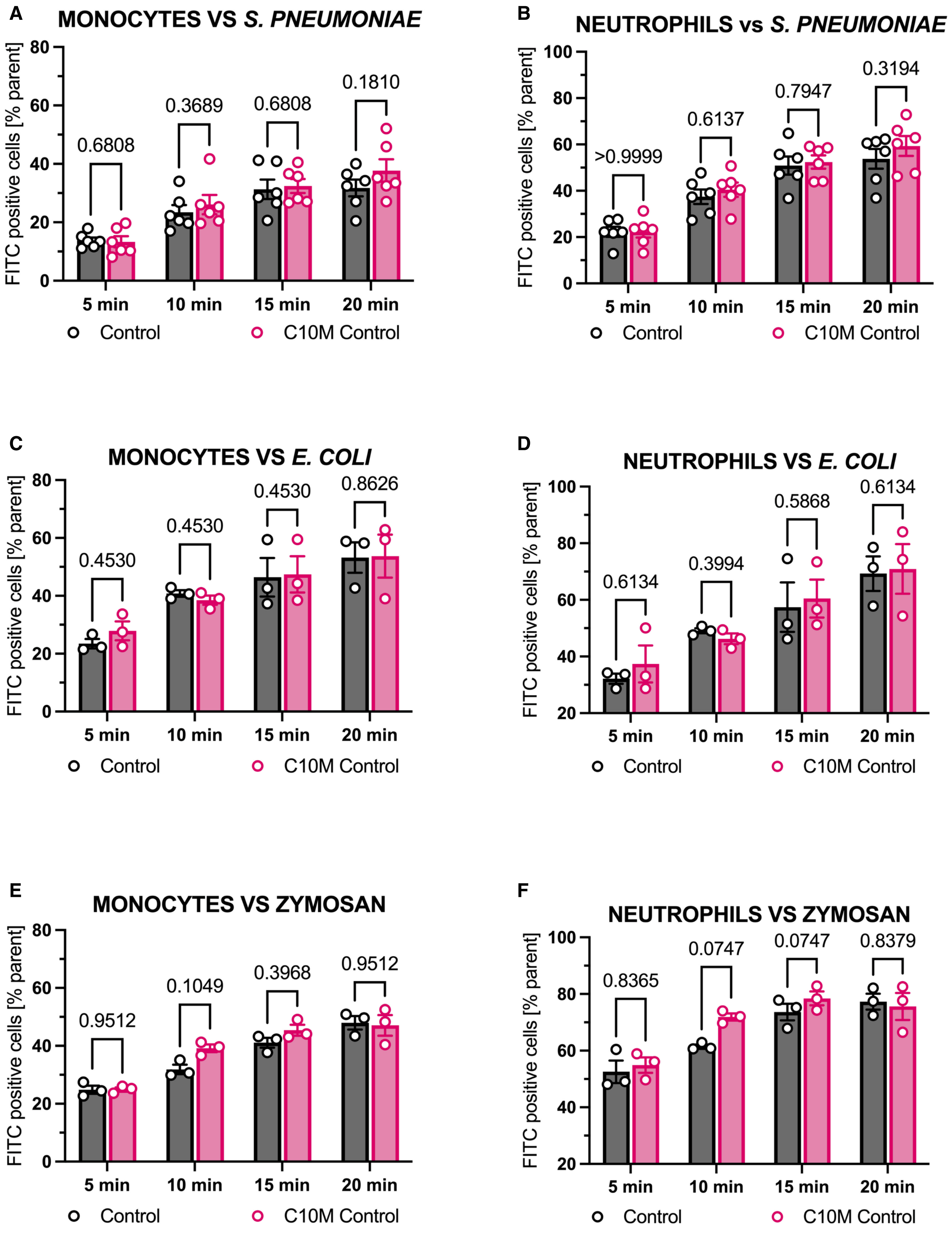

Figure EV3.

© 2022 The Authors
