# Supplementary material for: A novel phosphocholine‐mimetic inhibits a pro‐inflammatory conformational change in C‐reactive protein
Source: EMBO Mol Med. 2022 Dec 5;15(1):e16236. doi: 10.15252/emmm.202216236 (PMC9832874; doi:10.15252/emmm.202216236)
Supplement: Supplementary file 14 [file EMMM-15-e16236-s007.pdf]

EMBO Molecular Medicine

A novel phosphocholine-mimetic inhibits a pro-inflammatory conformational change of C-reactive protein

Johannes Zeller, Karen Cheung Tung Shing, Tracy Nero, James McFadyen, Guy Krippner, Balázs Bogner, Sheena Kreuzaler, Jurij Kiefer, Verena Horner, David Braig, Habiba Danish, Sara Baratchi, Mark Fricke, Xiaowei Wang, Michel Kather, Bernd Kammerer, Kevin Woollard, Prerna Sharma, Craig Morton, Geoffrey Pietersz, Michael Parker, Karlheinz Peter, and Steffen Eisenhardt

DOI: 10.15252/emmm.202216236

Corresponding authors: Steffen Eisenhardt (steffen.eisenhardt@uniklinik-freiburg.de) , Michael Parker (mparker@svi.edu.au), Karlheinz Peter (karlheinz.peter@baker.edu.au)

Review Timeline:

Submission Date:	29th Apr 22
Editorial Decision:	31st May 22
Author Correspondence:	15th Jun 22
Editor Correspondence:	15th Jun 22
Revision Received:	2nd Sep 22
Editorial Decision:	6th Oct 22
Revision Received:	29th Oct 22
Accepted:	6th Nov 22

Editor: Lise Roth

Transaction Report:

(Note: With the exception of the correction of typographical or spelling errors that could be a source of ambiguity, letters and reports are not edited. Depending on transfer agreements, referee reports obtained elsewhere may or may not be included in this compilation. Referee reports are anonymous unless the Referee chooses to sign their reports.)

31st May 2022

Dear Prof. Eisenhardt,

Thank you for the submission of your manuscript to EMBO Molecular Medicine, and please accept my apologies for the delay in getting back to you as one referee needed more time to complete his/her report. We have now received feedback from the three reviewers who agreed to evaluate your manuscript. They all acknowledge the interest of the study and are overall supporting publication of your work pending appropriate revisions. However, as you will see from the reports below, referee #3 raised a concern regarding the in vivo model used.

In a following consultation session, the other referees agreed with this point, and they mentioned that a rabbit model of ischemia/reperfusion injury would be more relevant. We would therefore suggest using such a model in a revised version of your manuscript. We are however aware that these experiments might be unrealistic, and would be open to alternatives. We could discuss this issue further if you wish.

Addressing the other reviewers' concerns in full will be necessary for further considering the manuscript in our journal, and acceptance of the manuscript will entail a second round of review. EMBO Molecular Medicine encourages a single round of revision only and therefore, acceptance or rejection of the manuscript will depend on the completeness of your responses included in the next, final version of the manuscript. For this reason, and to save you from any frustrations in the end, I would strongly advise against returning an incomplete revision.

Revised manuscripts should be submitted within three months of a request for revision; they will otherwise be treated as new submissions, except under exceptional circumstances in which a short extension is obtained from the editor.

When submitting your revised manuscript, please carefully review the instructions that follow below. We perform an initial quality control of all revised manuscripts before re-review; failure to include requested items will delay the evaluation of your revision.

We require:

1) A .docx formatted version of the manuscript text (including legends for main figures, EV figures and tables). Please make sure that the changes are highlighted to be clearly visible.

2) Individual production quality figure files as .eps, .tif, .jpg (one file per figure). For guidance, download the 'Figure Guide PDF' (https://www.embopress.org/page/journal/17574684/authorguide#figureformat).

3) A .docx formatted letter INCLUDING the reviewers' reports and your detailed point-by-point responses to their comments. As part of the EMBO Press transparent editorial process, the point-by-point response is part of the Review Process File (RPF), which will be published alongside your paper.

4) A complete author checklist, which you can download from our author guidelines (https://www.embopress.org/page/journal/17574684/authorguide#submissionofrevisions). Please insert information in the checklist that is also reflected in the manuscript. The completed author checklist will also be part of the RPF.

5) Please note that all corresponding authors are required to supply an ORCID ID for their name upon submission of a revised manuscript.

6) For data quantification: please specify the name of the statistical test used to generate error bars and P values, the number (n) of independent experiments (specify technical or biological replicates) underlying each data point and the test used to calculate p-values in each figure legend. The figure legends should contain a basic description of n, P and the test applied. Graphs must include a description of the bars and the error bars (s.d., s.e.m.). Please indicate exact p values.

7) We would also encourage you to include the source data for figure panels that show essential data. Numerical data should be provided as individual .xls or .csv files (including a tab describing the data). For blots or microscopy, uncropped images should be submitted (using a zip archive if multiple images need to be supplied for one panel). Additional information on source data and instruction on how to label the files are available at
.

8) Our journal encourages inclusion of *data citations in the reference list* to directly cite datasets that were re-used and obtained from public databases. Data citations in the article text are distinct from normal bibliographical citations and should directly link to the database records from which the data can be accessed. In the main text, data citations are formatted as

follows: "Data ref: Smith et al, 2001" or "Data ref: NCBI Sequence Read Archive PRJNA342805, 2017". In the Reference list, data citations must be labeled with "[DATASET]". A data reference must provide the database name, accession number/identifiers and a resolvable link to the landing page from which the data can be accessed at the end of the reference. Further instructions are available at .

9) We replaced Supplementary Information with Expanded View (EV) Figures and Tables that are collapsible/expandable online. A maximum of 5 EV Figures can be typeset. EV Figures should be cited as 'Figure EV1, Figure EV2" etc... in the text and their respective legends should be included in the main text after the legends of regular figures.
- For the figures that you do NOT wish to display as Expanded View figures, they should be bundled together with their legends in a single PDF file called *Appendix*, which should start with a short Table of Content. Appendix figures should be referred to in the main text as: "Appendix Figure S1, Appendix Figure S2" etc.
- Additional Tables/Datasets should be labeled and referred to as Table EV1, Dataset EV1, etc. Legends have to be provided in a separate tab in case of .xls files. Alternatively, the legend can be supplied as a separate text file (README) and zipped together with the Table/Dataset file.
See detailed instructions here:
.

10) The paper explained: EMBO Molecular Medicine articles are accompanied by a summary of the articles to emphasize the major findings in the paper and their medical implications for the non-specialist reader. Please provide a draft summary of your article highlighting
- the medical issue you are addressing,
- the results obtained and
- their clinical impact.
This may be edited to ensure that readers understand the significance and context of the research. Please refer to any of our published articles for an example.

11) For more information: There is space at the end of each article to list relevant web links for further consultation by our readers. Could you identify some relevant ones and provide such information as well? Some examples are patient associations, relevant databases, OMIM/proteins/genes links, author's websites, etc...

12) Conflict of interest: We updated our journal's competing interests policy in January 2022 and request authors to consider both actual and perceived competing interests. Please review the policy https://www.embopress.org/competing-interests and update your competing interests if necessary.

13) Every published paper now includes a 'Synopsis' to further enhance discoverability. Synopses are displayed on the journal webpage and are freely accessible to all readers. They include a short stand first (maximum of 300 characters, including space) as well as 2-5 one-sentences bullet points that summarizes the paper. Please write the bullet points to summarize the key NEW findings. They should be designed to be complementary to the abstract - i.e. not repeat the same text. We encourage inclusion of key acronyms and quantitative information (maximum of 30 words / bullet point). Please use the passive voice. Please attach these in a separate file or send them by email, we will incorporate them accordingly.
Please also suggest a striking image or visual abstract to illustrate your article as a PNG file 550 px wide x 300-600 px high.

14) As part of the EMBO Publications transparent editorial process initiative (see our Editorial at http://embomolmed.embopress.org/content/2/9/329), EMBO Molecular Medicine will publish online a Review Process File (RPF) to accompany accepted manuscripts.
In the event of acceptance, this file will be published in conjunction with your paper and will include the anonymous referee reports, your point-by-point response and all pertinent correspondence relating to the manuscript. Let us know whether you agree with the publication of the RPF and as here, if you want to remove or not any figures from it prior to publication.
Please note that the Authors checklist will be published at the end of the RPF.

EMBO Molecular Medicine has a "scooping protection" policy, whereby similar findings that are published by others during review or revision are not a criterion for rejection. Should you decide to submit a revised version, I do ask that you get in touch after three months if you have not completed it, to update us on the status.

I look forward to receiving your revised manuscript.

Yours sincerely,

Lise Roth

Lise Roth, PhD

Senior Editor
EMBO Molecular Medicine

***** Reviewer's comments *****

Referee #1 (Remarks for Author):

This manuscript contains an admirably comprehensive and well thought-out scientific work. It therefore has rather the size of half a book and frightens the reviewer.

Major and minor remarks for authors:

Title and throughout the manuscript:
"CRP activation" is an infelicitous term. This is about a conformational change.

Introduction:
Line 77/78: Publications missing here which show inflammatory effects under the same experimental design and another which demonstrated an immediate effect of injected human CRP on blood pressure:

• Bisoendial RJ, Kastelein JJ, Peters SL, Levels JH, Birjmohun R, Rotmans JI, et al. Effects of CRP infusion on endothelial function and coagulation in normocholesterolemic and hypercholesterolemic subjects. J Lipid Res. (2007) 48:952-60. doi: 10.1194/jlr.P600014-JLR200
• Bisoendial R, Birjmohun R, Keller T, van Leuven S, Levels H, Levi M, et al. In vivo effects of C-reactive protein (CRP)-infusion into humans. Circ Res. (2005) 97:e115-16. doi: 10.1161/01.RES.0000196746.75724.8b
• DOI: 10.1161/CIRCRESAHA.114.303216
• Front. Immunol. 11:1978. doi: 10.3389/fimmu.2020.01978
•
Line 79: ...ischemia and hypoxia and ... .
Line 79: Publications about clinical results missing here:
• Front Cardiovasc Med 8:591714. https://doi.org/10.3389/fcvm.2021.591714
• Front Immunol 12:708101. https://doi.org/10.3389/fimmu.2021.708101
• Am J Case Rep. https://doi.org/10.12659/AJCR.932964
• Am J Case Rep. https://doi.org/10.12659/AJCR.935263
• J. Clin. Med. 2022, 11,1956. https://doi.org/10.3390/jcm11071956

Line 80: ...human pCRP... --- This needs to be explained to get the full picture.
Line 80: and why are rabbit and pig with endogenous CRP ignored?:
• J. Clin. Apher. 2015, 30, 15-21. https://doi.org/10.1002/jca.21344
• J Pharmacol Exp Ther. (2002) 303:1007-13. doi: 10.1124/jpet.102.040600

Line 84: various literature on sPLA2 is missing here e.g.:
• Cardiovasc Res. (2002) 53:138–46. Doi: 10.1016/s0008-6363(01)00480-1
• Am J Physiol Heart Circ Physiol. (2003) 285:H2218-24. Doi: 10.1152/ajpheart.00887.2002

Line 95: PS is also exposed and in higher quantity than PE.

Line 116: missing recent literature:
• Front Cardiovasc Med 8:591714. https://doi.org/10.3389/fcvm.2021.591714
• Am J Case Rep. https://doi.org/10.12659/AJCR.935263
• J. Clin. Med. 2022, 11,1956. https://doi.org/10.3390/jcm11071956
• European Heart Journal, Volume 42, Issue 23, 14 June 2021, Pages 2280-2283, https://doi.org/10.1093/eurheartj/ehab169

Line 117-119: For ISIS and CRP apheresis, the maximum reduction of CRP concentration was approximately 76 %. How should this lead to immune suppression if the patient still has an increased CRP concentration and the liver has full synthesis capacity? As far as I remember, the ISIS patients were challenged by endotoxin to demonstrate the uncompromised immune response.
Line 123-126: I would be very happy to learn what advantage this has? Otherwise, the effect should be mentioned, but not called advantage.

The last sentence of the introduction reads like a conclusion. However, this should only occur at the end or in the abstract.

Results
Line 173: please provide moles and calculate the molar excess for C10M/pCRP. This is the more scientifically valuable description in this case.
Figure 1C: that's pretty small. Depending on the size of the image in print, you may not see C10M.
Supplemental figures: It is annoying because it is inconvenient for the reader if information is frequently referred to in the supplements, especially if it is new information. Especially in the case of Figure S3, it seems to me advisable to reconsider whether it should not be in the main text, at least the part about the NETs. This will interest the NETologists.
Line 204: Did you always use the same Ca2+ concentration in theses assays?
Line 274-275: NETosis is a mediator of inflammation but not only for sterile inflammation. So please remove the word "sterile".
Line 302ff: "C10M inhibits CRP-mediated allograft rejection in hindlimb transplantation". Can an effect of the high concentration of rat CRP be excluded? I don´t see it being even mentioned.
Line 325-326: Does CRP-8 bind rat CRP or pCRP*/mCRP?
General remark: What is the rational to use different CRP concentrations in the individual experiments? Please explain it in the manuscript.
Discussion:
Line 405-409: Why would blocked pCRP still be helpful in beneficial inflammatory processes? Is there any evidence for this?
Line 411: Why is the "oral route" mentioned? This is a step too far for C10M as long as there is no evidence for it.
Line 414-416: One would like to see a neutral discussion of other approaches. If potential disadvantages of CRP apheresis are to be discussed, then please also mention the advantages, such as practically no side effects and large amounts of CRP are removed from the body within hours instead of being blocked and still in the organism.
Line 452-476: In this section, curiously, CRP is mentioned in general and no longer the individual conformational variants. This leaves the stringent line of argumentation of the rest of the text.
Figures:
While it is a good idea to provide figures for lectures with headings describing the result of the experiment, one does not wish to do so for a publication, as these results are (supposed to be) described in the results section, please. Examples:
Fig. 1: C10M reduces binding of pCRP to immobilized PC.
Figure 2: C10M prevents pCRP binding to activated platelets. ... pCRP localizes on platelets, which can be inhibited by C10M. ... Platelets bind less CRP when incubated with C10M...
Figure 3: CRP-dependent expression of pro-inflammatory cytokines is abolished by C10M. ... Addition of C10M to the whole blood samples inhibits the CRP-dependent expression of pro-inflammatory cytokines.
And so on for all Figure legends. Please describe only what is needed to understand the figure.
Line 1463: what does the 35 mean?
Line 1468: Why do we see 3 CRP bands? This should be discussed in the results section.
Line 1489: it is not explained in the figure what exactly the control is?
Line 1543: of the PC groups of microvesicles, only the LPC derived PC groups can bind to the B-face of pCRP. The reader may not get the impression, that all PC groups are accessible.
Figure 8: Which CRP conformation is expected on the vesicles in the upper left scheme? To me it looks like pCRP.

Referee #2 (Comments on Novelty/Model System for Author):

This manuscript reports on the development and functional characterization of C10M, a phosphocholine-mimicking compound that inhibit formation of pCRP*/mCRP, which mediate the potent pro-inflammatory activities associated with CRP. The experiments are well-designed, properly controlled and powered, and the main conclusions are adequately supported by experimental data. The manuscript is of interest, but the authors need to address some specific points to increase the quality of the work.

While overall the model systems are appropriate, functional adhesion assays or assessment of NET formation in more depth could enhance the value the of the study.

Referee #2 (Remarks for Author):

The manuscript by Zeller and collaborators reports development of a phosphocholine-mimicking compound, compound C10M ((3-dibutylamino-propyl) phosphonic acid) that functions as a monovalent inhibitor of structural rearrangements in pCRP. The study combines X-ray crystallography, in vitro experiments and experiments in rat models of renal ischemia-reperfusion and graft rejection. The authors report that C10M binds to the PC/PE binding pocket of pCRP, and inhibits pCRP*/mCRP-evoked activation of monocytes, neutrophils and endothelial cells. Furthermore, treatment with C10M significantly attenuated CRP-mediated renal ischemia-reperfusion-induced and allograft rejection of hindlimb transplantation-associated tissue injury. The findings are of interest but the authors need to address some specific points to increase the quality of the work.
Major Comments.
1. The authors refer to CRP "activating" mechanism. Since native pCRP is known to play important roles in host defense (e.g.

opsonization of pathogens, activation of complement, inhibition of leukocyte adherence to the endothelium), "activation" is not a prerequisite for exerting biological functions. Indeed, "activation" appears to refer to changes in CRP conformation, leading to formation of pCRP* and then dissociation to mCRP. Is there a more precise way of describing "activation" (e.g. replacing with "conformational changes" or something similar)?
2. What was the rationale for using pCRP at various concentrations (ranging from 25-100 ug/ml) in the various experiments? It is unclear how these concentrations were chosen for the specific assays. 100 ug/ml appears to be a rather high concentration. Likewise, some information will be required on how C10M concentrations and in vivo doses were chosen.
3. Previous studies showed that monocyte recruitment is essential for timely resolution of inflammation. How could then C10M inhibition of pCRP*/mCRP-induced monocyte adhesion be reconciled with anti-inflammatory action?
4. C10M reduction of NETosis is interesting. However, the biological consequences of this effect cannot be deduced from the results presented for viable NETosis (as opposed to suicidal NETosis) is thought to play protective role when pathogens cannot be phagocytosed. Labeling of Figure S3 may be misleading for it depicts pCRP*/mCRP vs. CRP+C10M.
5. Can C10M interfere with mCRP binding or signaling through CD16 or lipid rafts?

Minor Points
1. Are reductions in ICAM-1 and VCAM-1 expression sufficient to prevent neutrophil or monocyte adherence to HUVEC?
2. The title to Figure 4 lists ROS and NET formation, while these measurements are not depicted on the figure.
3. Figure 5B. Please explain what the three curves illustrate. Also for panel D, please indicate the number of experiments for which representative results are shown.
4. Figure 8 depicts molecules/mechanisms (e.g. histone, elastase or chatepsin G) that are not addressed in the manuscript. Does C10M prevent pCRP* formation or binding of pCRP to cell membrane (as shown on the right panel)?
5. Lines 260 and 757. "HUVEC cell" is superfluous for "C" in HUVEC stands for "cell or cells".

Referee #3 (Comments on Novelty/Model System for Author):

The generated small molecule C10M able to inhibit the active forms of CRP is of great interest. The in vitro experiments are convincing of its efficacy but I have concerns about the suitability of the in vivo models that are based in the injection of human active CRP; a somehow artificial model.

Referee #3 (Remarks for Author):

This study reports the generation of a phosphocholine-mimicking tool compound (C10M) able to inhibit CRP activation. The design is based in the mechanism of activation of CRP identified by the authors in a previous study. It has the advantage of binding to inactive pentameric CRP (pCRP) preventing its interaction with bioactive lipids on activated cell membranes and its subsequent activation. X-ray crystallography studies show binding of C10M to pCRP. In vitro and in vivo studies reveal the ability of C10M to inhibit CRP-driven exacerbation of inflammatory responses. The in vitro models are convincing, but I have some concerns about the in vivo models.

Major points:
1. Figs 4D, 4E and S3B: the effect of C10 M is not statistically significant. Is is possible to use pre-activated platelets and analyzed other markers?
2. Figs 5 and 6: It can be read in the discussion that the rat model is ideal to investigate the role of human CRP to inflammation and the impact of C10M. However, I think injection of human pCRP is an artificial model, since it is observed the impact of C10M in the exacerbated pathology caused by human pCRP. Is is possible to use alternative animal models to analyze the impact of C10M in endogenously activated CRP?
3. Fig. 7: This experiment is important to evaluate if C10M only inhibit the pathological effects of CRP. Therefore, the effects of C10M in pCRP-induced phagocytosis needs to be studied. In addition, this in vitro result may be further validated by analyzing the effects of C10M in in vivo infection experiments.

Minor points:
1. All figures: labeling each panel will facilitate the interpretations of the results w/o the need of the legend that it is quite tedious.
2. Fig. 5C: label the CRP deposition
3. Fig. S3C: the labeling used is confusing and does not match the legend. The effects of C10M in NDET formation needs quantification.
4. Discussion: is there any advantage of C10M over bis-PC? Please, discuss.

Freiburg, the 15th of June 2022

Univ.-Prof. Dr. med. S.U. Eisenhardt
Full Professor and acting Chairman
Dept. Plastic and Hand Surgery - University of Freiburg Medical Center

To
Lise Roth, PhD
Senior Editor
EMBO Molecular Medicine

Regarding Manuscript #EMM-2022-16236

Dear Dr Roth,

Thank you very much for providing us with the overall positive Reviewer comments and the general interest in our study. We appreciate the opportunity you have given us to contact you regarding your and the Reviewers' comments.

We acknowledge the reported concerns regarding the optimal animal model for CRP research and in particular the question of whether a rabbit model of ischemia/reperfusion injury would be needed to provide further in vivo evidence for the inhibitory effects of our anti-CRP compound.

We have thoroughly considered the logistics of performing the requested rabbit in vivo model. A realistic time frame for us to establish this model, including ethics, and performing the required group comparisons will take us 2 to 3 years. Indeed, establishing the hindlimb ischemia/reperfusion model in rats has taken us 4 years and, despite having experience in one species, it may not be straightforward to translate our unique expertise in rats into a rabbit model. Establishing the rabbit model would seriously delay publication of our exciting results. We are sure our work will inspire follow up studies by many other labs around the world and hence timely publication is highly desirable.

Whilst we understand the concerns raised by referee #3 regarding the potentially artificial nature of the rat model and the request for a model in which endogenous CRP plays a more central role, we wish to highlight that the use of rats as an animal model in combination with human CRP (the actual target of our compounds) is nonetheless well established, and commonly utilized, in the field of CRP biology by other groups (Griselli et al. (J Exp Med., 1999); Gill et al. (J Cereb Blood Flow Metab., 2004); Pepys et al. (Nature, 2006) and our workgroup Thiele et al. (Circulation, 2014); Braig et al. (Nature Com, 2017). As such, although we agree that a rabbit model would provide further proof of concept, we believe that our current in vivo data, based on several years of establishing a quite unique and clinically relevant transplant model, is very strong and well-controlled. We would thus ask the Editor and Reviewers to consider our opinion that the additional experimental support that would be obtained by additional rabbit

experiments would not justify the enormous effort and time required necessary to establish and perform this additional work.

We are grateful for your consideration of these issues. Since we can address all other requests, including performing additional experiments as suggested, we would be most grateful for the approval to submit a revised version of our manuscript within 3 months without the need to perform additional rabbit experiments.

Thank you very much for your consideration and we are looking forward to your response.

Yours sincerely,

Steffen U. Eisenhardt
On behalf of the communicating authors.

Dear Prof. Eisenhardt,

Thank you for your email and careful consideration of the reviewers' requests.
I have discussed your letter with my colleagues, and we agree that establishment of a rabbit model of ischemia/reperfusion would take a lot of time and effort, which would be detrimental to the timely publication of your work. Therefore, we would be happy for you to instead discuss this issue in the manuscript and in your point-by-point rebuttal letter to the referees.

Looking forward to receiving your revised manuscript,

With kind regards,

Lise Roth

Lise Roth, PhD
Senior Editor
EMBO Molecular Medicine
l.roth@embomolmed.org

Manuscript Number EMM-2022-16236, "A novel phosphocholine-mimetic inhibits a pro-inflammatory conformational change of C-reactive protein"

Response to the reviewer comments

We thank the editor and the referees for their very positive evaluation of our manuscript and the highly constructive comments. In the following, we provide a detailed point-by-point response to the editor's and referees' comments. We repeat the reviewer comments in bold and our answer in indented paragraphs. Changes in the revised manuscript are highlighted in blue color.

Specific response to the editor:

As requested by the editor to clarify data quantification (Q6), we now indicate the exact p-values in every graph. In addition, all statistical test outcomes are now provided in each graph and the number of independent experiments are now listed in the figure legends.

We have amended the graph style as requested to a bar graph with single data dots visible (scatter plot with bar) and colorblind-safe color settings. Further, the citation style was changed to the style used by EMBO Molecular Medicine. Changes or additional references in the revised manuscript are indicated are highlighted in yellow.

Following the recommendation of two referees, we have changed the title of our manuscript to "A novel phosphocholine-mimetic inhibits a pro-inflammatory conformational change of C-reactive protein", thereby avoiding the ambiguous term "CRP activation".

The font style has been changed to Arial within all figures, including the graphical abstract, in Figure 5, 6, and 8, respectively. Furthermore, we have amended the graphical abstract in Figure 5 (Fig 5A) and Figure 6 (Fig 6A) depicting the surgical procedures to allow for easier understanding.

In accordance with the style of formatting of EMBO Molecular Medicine, we now refer to the figures as "Fig" rather than "Figure", e.g., "Fig 1A" which was before "Figure 1A".

The formatting of the figures was revised to allow for the titles of each panel. Consequently, for a better use of the space, the order in Fig 5 was changed.

Figure 7 has been edited to now demonstrate phagocytosis data from three target species, i.e., Streptococcus pneumoniae, Escherichia coli, and zymosan from Saccharomyces cerevisiae, and two phagocytes, i.e., human monocytes and neutrophils. We added further phagocytosis data in the respective EV figure.

Referee #1 (Remarks for Author):

This manuscript contains an admirably comprehensive and well thought-out scientific work. It therefore has rather the size of half a book and frightens the reviewer.

Answer:
We thank the referee for their kind words and appreciation of our efforts.
In the following, we provide a detailed point-by-point response to the referee's questions and concerns. Our apologies for changes in the referred lines: Due to an adaption to the formatting the lines of the original manuscript and the referee's comments and the revised manuscript respectively, may differ.

Major and minor remarks for authors:

Title and throughout the manuscript:
"CRP activation" is an infelicitous term. This is about a conformational change.

Answer:
We thank the Referee for raising this important point. We agree that this term may not be the most appropriate, and accordingly we have amended the title of our revised manuscript as below and omitted the term "CRP activation" throughout the manuscript accordingly. We trust this provides a more precise description of the CRP biology described. The new title of the manuscripts is now:

"A novel phosphocholine-mimetic inhibits a pro-inflammatory conformational change of C-reactive protein"

Introduction:
Line 77/78: Publications missing here which show inflammatory effects under the same experimental design and another which demonstrated an immediate effect of injected human CRP on blood pressure:
• Bisoendial RJ, Kastelein JJ, Peters SL, Levels JH, Birjmohun R, Rotmans JI, et al. Effects of CRP infusion on endothelial function and coagulation in normocholesterolemic and hypercholesterolemic subjects. J Lipid Res. (2007) 48:952-60. doi: 10.1194/jlr.P600014-JLR200
• Bisoendial R, Birjmohun R, Keller T, van Leuven S, Levels H, Levi M, et al. In vivo effects of C-reactive protein (CRP)-infusion into humans. Circ Res. (2005) 97:e115-16. doi: 10.1161/01.RES.0000196746.75724.8b
• DOI: 10.1161/CIRCRESAHA.114.303216
• Front. Immunol. 11:1978. doi: 10.3389/fimmu.2020.01978

Answer:
Indeed, these are important papers to cite, and therefore we have added a more thorough discussion of these effects of human CRP. We added this paragraph in the discussion of our manuscript to allow for a more thorough discussion, fitting to its relevance. We hope these changes provide a comprehensive discussion of the issue of CRP addition in different models. We added this information in line 612ff:

"Whilst purified native human pCRP itself is described to be not pro-inflammatory when injected into healthy individuals (Lane et al, 2014), earlier studies using recombinant human CRP reported endothelial dysfunction and augmented pro-coagulant responses, which were not attributed to potential LPS contaminations (Bisoendial et al, 2005, 2007). Notably, purified native human pCRP injected in healthy rabbits showed a significant blood pressure drop (Bock et al, 2020). These

partially contradictory findings highlight the need for further research on the direct effects of pCRP."

Line 79: ...ischemia and hypoxia and ... .

Answer:
We changed the wording accordingly (line 63ff):

"There is strong evidence that administration of human pCRP can significantly increase tissue injury in animal models of myocardial infarction (Griselli et al, 1999; Pepys et al, 2006), stroke (Gill et al, 2004) and other organ ischemia/hypoxia and ischemia/reperfusion injuries (IRI) (Padilla et al, 2007; Thiele et al, 2018; McFadyen et al, 2018)."

Line 79: Publications about clinical results missing here:
- **Front Cardiovasc Med 8:591714. https://doi.org/10.3389/fcvm.2021.591714**
- **Front Immunol 12:708101. https://doi.org/10.3389/fimmu.2021.708101**
- **Am J Case Rep. https://doi.org/10.12659/AJCR.932964**
- **Am J Case Rep. https://doi.org/10.12659/AJCR.935263**
- **J. Clin. Med. 2022, 11,1956. https://doi.org/10.3390/jcm11071956**

Answer:
Again, we thank the referee for the effort in forming a more comprehensive introduction. We included the suggested publications in the references (line 71ff):

"In line with the aggravating effects of human pCRP*/mCRP in animal models, recent clinical studies revealed a correlation between CRP concentration and cardiac infarct size (Ries et al, 2021), and clinical deterioration and harmful inflammatory responses in COVID-19 patients. Importantly, the therapeutic reduction of CRP levels by CRP-apheresis was highly beneficial in these clinical settings (Ringel et al, 2021; Torzewski et al, 2021; Schumann et al, 2021; Esposito et al, 2022)."

Line 80: ...human pCRP... --- This needs to be explained to get the full picture.

Answer:
We agree with the Referee that this is an important point. Therefore, we have added further information regarding the use of human CRP in rat models (line 67ff):

"Furthermore, human CRP activates the rat complement system after dissociation to pCRP*/mCRP on the sites of inflamed tissue and thereby aggravates the ongoing inflammation, while rat CRP has been described not to activate autologous complement (de Beer et al, 1982; Griselli et al, 1999; Thiele et al, 2014; Braig et al, 2017)."

Line 80: and why are rabbit and pig with endogenous CRP ignored?:
- **J. Clin. Apher. 2015, 30, 15-21. https://doi.org/10.1002/jca.21344**
- **J Pharmacol Exp Ther. (2002) 303:1007-13. doi: 10.1124/jpet.102.040600**

Answer:
We thank the referee for raising this important point. As suggested, and to allow for a more comprehensive overview, we have added the highlighted studies and provide a wider discussion about the animal models used in CRP research. We have also added a discussion regarding the potential limitations of our model and the potential

for future work utilizing alternative other animal models with endogenous CRP (line 599ff):

"The application of human pCRP in rats is a widely accepted animal model, as it mimics most effects human CRP has in humans in a small animal model. Therefore it has become particularly valuable in models testing therapeutic strategies (Pepys *et al*, 2006) as it is the only model where the final protein target, human CRP *in vivo*, can be tested. However, the application of human pCRP into the rat has limitations based on the xenogenic nature of this approach and may also not fully reflect CRPs pathophysiological role in humans. Therefore, in the future additional validation should be conducted in animal models with endogenous CRP, such as rabbits and pigs (Barrett et al, 2002; Sheriff et al, 2015), to further preclinical drug development."

Line 84: various literature on sPLA2 is missing here e.g.:
• Cardiovasc Res. (2002) 53:138−46. Doi: 10.1016/s0008-6363(01)00480-1
• Am J Physiol Heart Circ Physiol. (2003) 285:H2218-24. Doi: 10.1152/ajpheart.00887.2002

Answer:
In response to the Referee's comments, we added the recommended literature (line 79ff):

"In the context of inflammation and tissue damage, membrane changes on activated cell membranes mediated by phospholipase A2 lead to the exposure of bioactive lipids (Thiele et al, 2014; Nijmeijer et al, 2002, 2003)."

Line 95: PS is also exposed and in higher quantity than PE.

Answer:
We thank the referee for this important comment. Accordingly, we have added PS to the listed ligands of pCRP.
Indeed, as highlighted by the referee, PS is also exposed to the surface of activated and damaged cells and usually in higher quantities than PC and PE, respectively. Furthermore, PS binds to the PC binding site of pCRP as does PC and PE. Interestingly, unpublished data from our group indicate that PS binds weaker than PC in both ELISA and SPR. We modified the statement accordingly (line 94ff):

"Phosphocholine (PC), phosphatidylserine (PS), and phosphoethanolamine (PE) head groups of bioactive lipids are exposed on the surface of activated and damaged cells. The PC, PS, and PE head groups are known to bind to a shallow groove containing two calcium cations located on one face of the monomeric subunit of pCRP, hence there are five PC/PE binding pockets on the same face of the CRP pentamer (i.e., the B-face)."

Line 116: missing recent literature:
• Front Cardiovasc Med 8:591714. https://doi.org/10.3389/fcvm.2021.591714
• Am J Case Rep. https://doi.org/10.12659/AJCR.935263
• J. Clin. Med. 2022, 11,1956. https://doi.org/10.3390/jcm11071956
• European Heart Journal, Volume 42, Issue 23, 14 June 2021, Pages 2280-2283, https://doi.org/10.1093/eurheartj/ehab169

Answer:

We thank the Referee for pointing out this oversight. We have now added the suggested citations (line 116ff):

"Furthermore, reducing circulating pCRP levels via CRP-apheresis using PC-linked resins is currently being investigated with promising initial results as an adjunct therapy to minimize cardiac injury in patients with myocardial infarction (Ries et al, 2019, 2021) and COVID-19 (Esposito et al, 2022; Ringel et al, 2021; Torzewski et al, 2021; Pepys, 2021; Schumann et al, 2021)."

Line 117-119: For ISIS and CRP apheresis, the maximum reduction of CRP concentration was approximately 76 %. How should this lead to immune suppression if the patient still has an increased CRP concentration and the liver has full synthesis capacity? As far as I remember, the ISIS patients were challenged by endotoxin to demonstrate the uncompromised immune response.

Answer:
We thank the referee for these critical and well-justified comments. We agree that there is no indication that the reduction of CRP levels achieved may compromise the immune response and that our closing remark of this paragraph was rather speculative. In response to the referee's comment, we now omit the sentence "However, such broad therapeutic inhibition of CRP may come at the cost of some level of immune suppression, which can potentially lead to significant side effects."
Instead, we state the following (line 122ff):

"However, one theoretical concern of CRP reducing therapies is the potential risk of immunosuppression, especially in bacterial infections and sepsis, given CRP's well-described role in the innate immune response. Intriguingly, in the ISIS studies, evaluating the reduction of endotoxin-induced increase in CRP levels by pre-treatment with antisense oligonucleotides (ASO), healthy endotoxin-challenged volunteers did not demonstrate any evidence of a compromised immune response and the ASO was well tolerated with no serious adverse events (Noveck et al, 2014). Moreover, in the phase II clinical trial, patients treated with the CRP ASO demonstrated no increase in infections compared to control patients (Warren et al, 2015). Other CRP lowering approaches, such as apheresis, have also shown that the therapeutic targeting of CRP does not appear to increase the risk of infection (Torzewski et al, 2020; Ries et al, 2021; Torzewski et al, 2021; Ringel et al, 2021; Schumann et al, 2021; Torzewski et al, 2022). Therefore, therapeutic targeting of CRP is an attractive anti-inflammatory therapy approach and different strategies have been developed and are being tested with promising initial results."

Line 123-126: I would be very happy to learn what advantage this has? Otherwise, the effect should be mentioned, but not called advantage.

Answer:
We thank the Referee for this question and the opportunity to extend the discussion comparing the monovalent C10M and the bivalent bis-PC (see figure below).

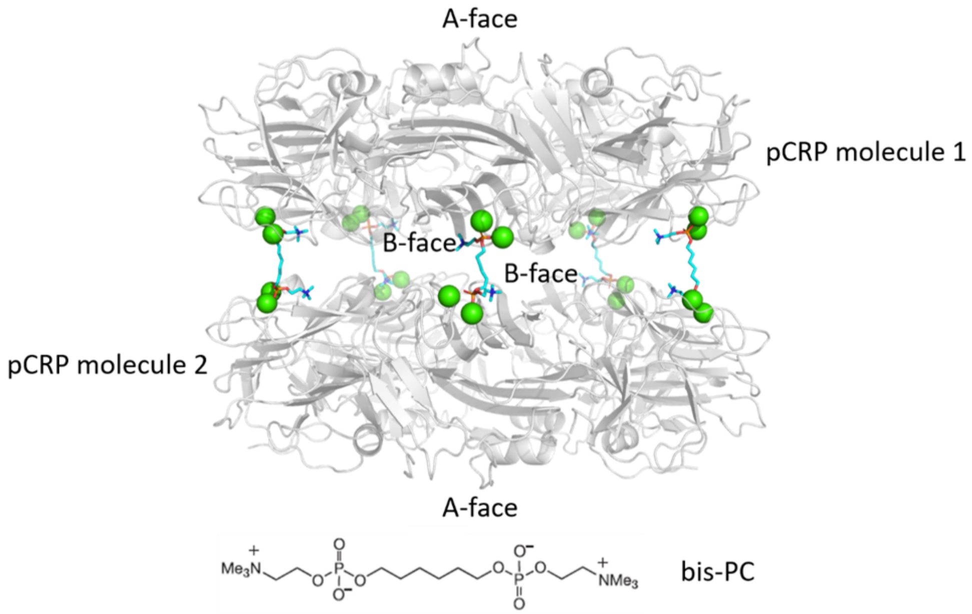

Modelled bis-PC:pCRP complex as per Pepys *et al* Nature 2006, 440:1217-21. Calcium ions are shown as green spheres, bis-PC is shown in stick format with light blue carbon atoms. Pentameric CRP is depicted as grey cartoon.

The mode of action of bis-PC is blocking the access to the whole B-side of two molecules of pCRP by interlocking these face-to-face (as illustrated in the figure above). However, C10M is blocking only the PC binding sites, leaving the remainder of the B-face of pCRP otherwise accessible; thereby, we assume that (beneficial) additional protein-protein interaction(s) can still take place. We now discuss our approach in more detail (line 443ff):

"The monovalent compound strategy allows both the A-face and B-face, with the exception of the PC binding pocket, of the CRP pentamer to remain available to interacting partner proteins and their associated functions. In contrast, only the A-face of the CRP pentamer is available for interaction when a bivalent compound like bis-PC is utilized. Proteins known to interact with the B-face of pCRP and/or pCRP* include the fibrinogen-like domain of M-ficolin, FH-like protein-1, and human neutrophils (Buchta et al, 1987; Okemefuna et al, 2010a; Zhang et al, 2011). Furthermore, there is evidence that the B-face of pCRP binds to proteins whose secondary structure is predominantly β-sheet (e.g., Amyloid-β1-38), as well as misfolded or aggregated proteins; and that this interaction is independent of the PC binding pocket and located in the vicinity of the interfaces between the monomers in the CRP pentamer (Singh et al, 2009; Hammond et al, 2010)."

Further, ongoing research in our labs suggests an interaction of misfolded proteins with the B-face of pCRP without utilization of the PC binding site (unpublished confidential data, illustrated by some of our modelling results below). Intriguingly, pentameric CRP and SAP were described to function as extracellular chaperones, and pCRP appears to interact calcium-independently with β-sheets forming amyloid, showing anti-amyloidogenic behavior (Ozawa *et al*, 2016). The calcium-independent interaction however, indicates an interaction outside the PC binding site.

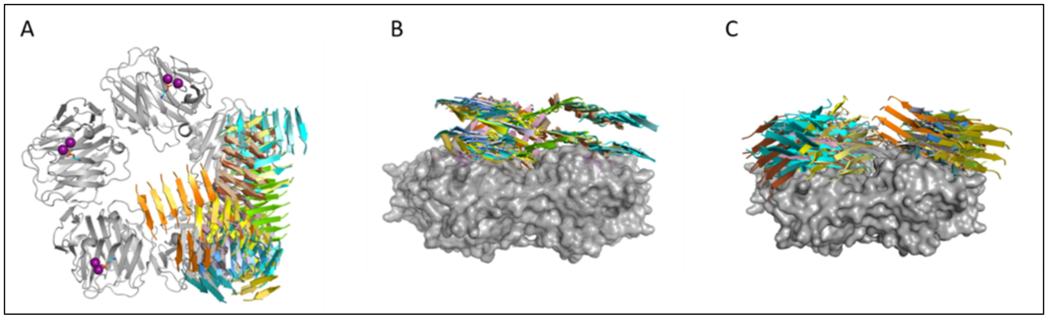

Results of Amyloid-β16-21 (KLVFFA, PDB ID:30W9) and Amyloid-β35-42 (MVGGVVIA, PDB ID:2Y3L) β-sheet blind docking with two monomers from the PE:pCRP complex (PDB ID: 3L2Y). In panel **A**, pCRP is shown as a grey cartoon, Ca^{2+} as purple spheres and bound PE as colored sticks. **B**, Side view of the PE:pCRP complex, clearly shows the orientation of the Amyloid-β16-21 β-sheets. **C**, Side view of the PE:pCRP complex, showing the orientation of the longer Amyloid-β35-42 β-sheets. In B and C, pCRP is depicted as a grey molecular surface.

However, since we are not showing any proof in this study that these protein interactions are primarily beneficial, we have changed the wording accordingly and incorporating the Referee's comment, no longer call the monovalent compound strategy advantageous over the previously published approach of bis-PC (line 137ff):

"We set out to design a low molecular weight monovalent tool compound that targets the PC/PE binding pocket on pCRP and thereby has the potential to prevent the formation of the pro-inflammatory pCRP* and mCRP species. Our strategy follows a distinctly different design compared to the previously published bivalent approach, as it allows the CRP pentamer to remain intact and in circulation, with both the A- and B-faces available to interact with ligands and retain associated functions."

The last sentence of the introduction reads like a conclusion. However, this should only occur at the end or in the abstract.

Answer:
As suggested by the referee we have now deleted the last sentence of the introduction.

Results
Line 173: please provide moles and calculate the molar excess for C10M/pCRP. This is the more scientifically valuable description in this case.

Answer:
We thank the referee for this important comment. We have updated the information accordingly to molar excess for C10M:pCRP throughout the manuscript.

Figure 1C: that's pretty small. Depending on the size of the image in print, you may not see C10M.

Answer:
We understand the concern of the Referee. The purpose of Figure 1C was to show the packing of the four CRP pentamers within the unit cell of the crystal structure, however, we have now changed the display of the C10M molecules in this figure

panel from sticks to spheres so that the C10M molecules can be clearly identified, regardless of image size. Figure 1D is the figure panel that shows in detail how C10M interacts with residues in a CRP monomer.

Supplemental figures: It is annoying because it is inconvenient for the reader if information is frequently referred to in the supplements, especially if it is new information. Especially in the case of Figure S3, it seems to me advisable to reconsider whether it should not be in the main text, at least the part about the NETs. This will interest the NETologists.

Answer:
Following the referee's advice and formatting style of EMBO Molecular Medicine, we have now rearranged several data sets and transferred data about pCRP*/mCRP-induced NETosis previously shown in Supplemental Figures to Figure 4 and the additional information to Expanded View 4 (please find below). We hope these changes improved the clarity of the manuscript.

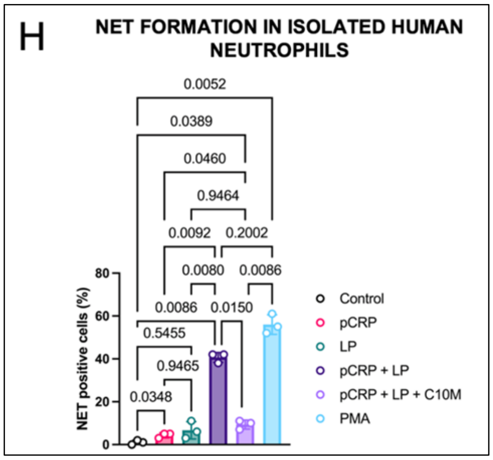

Figure 4: pCRP binding to ADP-activated platelets is inhibited by C10M, reducing expression of adhesion molecules involved in leukocyte diapedesis in endothelial cells and leukocytes, ROS formation and NET formation.
H pCRP*/mCRP dependent NETosis in isolated human neutrophils detected by confocal immunofluorescence microscopy. Isolated neutrophils incubated for 3 hours at 37 °C, 5% CO_2 with 100 µg/ml pCRP with and without PC:LPC liposomes and C10M (molar ratio 1:100, pCRP:C10M), respectively. Control was left unstimulated and 100 nM PMA served as positive control. Cells were washed, fixed and stained and analyzed by confocal microscopy. Results are given as a ratio of NETing cells/all cells per ROI, with mean ± SEM. P values were calculated with ANOVA and Tukey's post-hoc test. n=3.

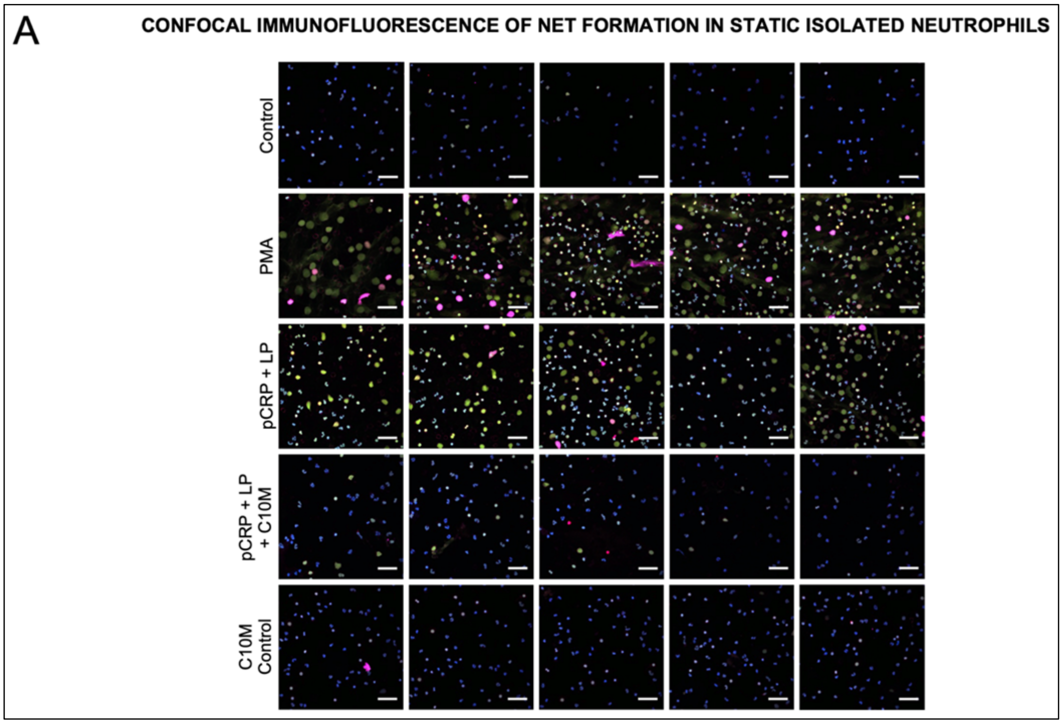

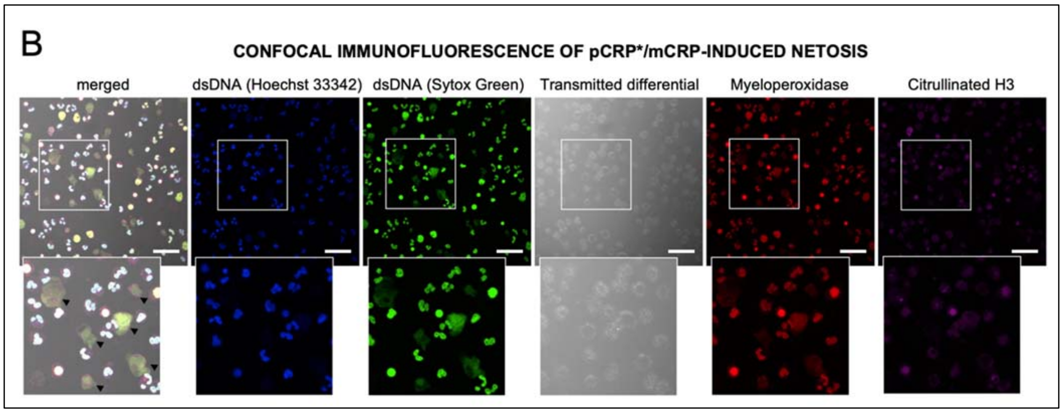

Figure EV2: Inhibitory effects of C10M on pCRP*/mCRP-induced NETosis and leukocyte-endothelial interaction

A Representative confocal immunofluorescence images of isolated human neutrophils as summarized in Fig. 4H. Cells were stained with anti-dsDNA dyes Hoechst 33342 and Sytox Green, and anti-MPO and anti-citH3 antibodies. Shown are uncropped images of merged channels and at 40x magnification. Scale bars indicate 50 µm.

B Representative single-channel confocal immunofluorescence images of pCRP*/mCRP-induced NETosis. Cells demonstrating NETs in the pCRP*/mCRP-stimulated group showed disrupted cell membranes as visible in the transmitted differential and merged channel (black arrow heads), indicating a suicidal mode of NETosis. Scale bars indicate 50 µm.

Line 204: Did you always use the same Ca2+ concentration in theses assays?

Answer:
We used two different experimental setups: (1) calcium-depleted platelet solutions, which were prepared by isolation of platelet rich plasma from whole blood anticoagulated with 3.2% trisodium citrate solution and washed two times against sequestrene buffer (containing 8.9 mM Na_2EDTA, pH 6.9). And (2) calcium-depleted platelet solutions (as described above) supplemented with calcium chloride (13 mM). No other concentrations were used. In response to the referee's comment, we now clearly state this (line 225ff):

"Controls showed calcium-dependent binding of CRP to platelets and no binding to isolated platelets in the absence of calcium: washed platelets in EDTA buffer showed no CRP binding, while washed platelets reconstituted with calcium showed CRP binding (shown in Appendix S1)."

We give the precise concentrations of calcium used in the methods (line 820 ff):

"100 µl of the solution was incubated with 50 µg/ml pCRP-Atto 594 with or without 13 mM $CaCl_2$ for 15 min at 37 °C, 5% CO_2. 2 µl of anti-CD62P PE (clone AK-4, BD Pharmingen™) were added to each sample and transferred to 350 µl FACS buffer 0.5% BSA and fixed with 50 µl 4% paraformaldehyde in PBS. Flow cytometry was performed within 30 min."

Line 274-275: NETosis is a mediator of inflammation but not only for sterile inflammation. So please remove the word "sterile".

Answer:
We followed the Referee's suggestion and removed "sterile" (line 304ff):

"A novel finding of our investigation into the mode of action of CRP-regulated inflammation is that pCRP*/mCRP induces formation of neutrophil extracellular traps (NETs) (Fig 4H and EV2A), a process called NETosis. NETosis is a mediator of inflammation, a key event that modulates tissue and organ damage (Cahilog et al, 2020)."

Line 302ff: "C10M inhibits CRP-mediated allograft rejection in hindlimb transplantation". Can an effect of the high concentration of rat CRP be excluded? I don´t see it being even mentioned.

Answer:
We thank the Referee for this important question. Yes, we previously performed experiments without exogenous human CRP added in hindlimb transplantation. Furthermore, the survival time of the allograft in the control animals of the hindlimb transplantation model (transplantation Lewis→Brown-Norway without application of human CRP) is given in Figure 6. Regarding the endogenous rat CRP, we hypnotize that the initial ischemia-reperfusion-injury during the hindlimb transplantation affect all animals equally regardless of the later administered human CRP, and prolonged and significantly differently periods of ischemia, which might would lead to a bias by endogenous effects, was an exclusion criterion for all rats. Rat CRP, however, is described to show no significant dynamic or function as an acute phase reactant (de Beer *et al*, 1982; Griselli *et al*, 1999).

Brown-Norway rats with transplanted Lewis hindlimbs showed acute rejection after a mean of 8 days (control). These animals served as a baseline assuming that the tissue injury due to the autologous hindlimb resection would lead to an equal increased of rat CRP throughout the treated groups. Further, we attributed the accelerated allograft rejection as a human CRP-specific effect, since a C10M control alone showed no effects on the allograft survival.
We added information regarding this issue in our introduction (line 67ff):

"Furthermore, human CRP activates the rat complement system after dissociation to pCRP*/mCRP on the sites of inflamed tissue and thereby aggravates the ongoing inflammation, while rat CRP has been described not to activate autologous complement (de Beer et al, 1982; Griselli et al, 1999; Thiele et al, 2014; Braig et al, 2017)."

and discussion (line 591ff and 599ff):

"For our *in vivo* experiments we utilized rat models. Although rats have abundant pCRP (300 – 600 µg/ml plasma in normal healthy pathogen-free rats), rat CRP is not utilized as an acute phase protein and rat complement is not activated by rat CRP (de Beer et al, 1982)."

"The application of human pCRP in rats is a widely accepted animal model, as it mimics most effects human CRP has in humans in a small animal model. Therefore, it has become particularly valuable in models testing therapeutic strategies (Pepys *et al*, 2006) as it is the only model where the final protein target, human pCRP *in vivo*, can be tested. However, the application of human pCRP into the rat has limitations based on the xenogenic nature of this approach and may also not fully reflect CRP's pathophysiological role in humans. Therefore, in the future additional validation should be conducted in animal models with endogenous CRP, such as rabbits and pigs (Barrett *et al*, 2002; Sheriff *et al*, 2015), to further preclinical drug development."

Line 325-326: Does CRP-8 bind rat CRP or pCRP*/mCRP?

Answer:
We thank the Referee for this question and pointing out the ambiguity of our wording.
The producer (Sigma-Aldrich) of the antibody anti-human CRP clone CRP-8 describes a specificity for human C-reactive protein. Cross-reactivity of the antibody clone CRP-8 is not known. Additionally, tissue samples from control rats (without administration of human CRP) undergoing the renal I/R-model or hindlimb transplantation model, respectively, were consistently negative for CRP-8 binding, e.g., Fig 5C, upper right panel "IRI". Therefore, to our best knowledge, clone CRP-8 neither detects rat pCRP nor neo-epitope expressing forms of rat CRP, if existing.
Clone CRP-8 is known to detect the pro-inflammatory isoforms of human CRP, i.e., pCRP* and mCRP, with higher affinity than human pCRP (Schwedler *et al*, 2003; Zhang *et al*, 2018). Whilst we cannot exclude a deposit of rat CRP in the tissue, the design of C10M targets primarily the inhibition of the conformational change of human CRP, and clone CRP-8 detects the active isoforms of the exogenous human CRP deposited in injured rat tissue.
We changed the wording to underline that we detected human pCRP*/mCRP and avoid ambiguity (line 368ff):

"Furthermore, we analyzed the amount of deposited human pCRP*/mCRP in the tissue by Western blotting with antibody clone CRP-8 and found human pCRP*/mCRP significantly reduced in both muscle and skin (Fig 6F)."

General remark: What is the rational to use different CRP concentrations in the individual experiments? Please explain it in the manuscript.

Answer:
We thank the Reviewer for noting this. Concentrations in the *in vivo* rat experiments followed earlier works of our group (Thiele *et al*, 2014; Braig *et al*, 2017; Thiele *et al*, 2018) and were based on measurements after i.p. administration of human pCRP. We added this information in the manuscript (line 561ff):

"To validate the effect of C10M *in vivo*, we used two distinct animal models of inflammation. Firstly, we exploited an established renal IRI model in rats (Thiele *et al*, 2018). In this model, the administration of human pCRP at previously described concentrations (25 µg/ml serum) demonstrated an aggravated inflammatory response to IRI (Thiele *et al*, 2014; Braig *et al*, 2017; Thiele *et al*, 2018). We demonstrated that the dissociation of human pCRP to pCRP*/mCRP leads to enhanced leukocyte activation, tissue infiltration and generation of ROS resulting in aggravation of tissue injury (Thiele *et al*, 2018). This model represents an established model to investigate the anti-inflammatory properties of C10M as IRI represents the prototypic, sterile inflammatory setting that results in increased tissue damage."

And line 583:

"In the VCA model the acceleration of acute allograft rejection by dissociation of exogenous human CRP in previously characterized concentrations is reversed by administration of C10M."

Further, the concentrations used in the *in vitro* assays followed either previously described methods (100 µg/ml in opsono-phagocytosis (Mold *et al*, 2001)), or were 25 µg/ml and 50 µg/ml, respectively, since these concentrations showed significant activating effects in the respective assay and publications. All used concentrations are well within the CRP levels seen in acute phase reactions (Pepys *et al*, 1978). We state this now in line 524ff:

"Further, we adjusted the initial pCRP concentration in our *in vitro* assays (25 µg and 50 µg per ml) so that we were able to show a significant increase in leukocyte activation (CD11b expression, ROS generation, expression of pro-inflammatory cytokines) and endothelial cell activation (ICAM-1 and VCAM-1 expression, leukocyte adhesion), respectively."

Discussion:
Line 405-409: Why would blocked pCRP still be helpful in beneficial inflammatory processes? Is there any evidence for this?

Answer:
The design of the presented small molecule inhibitor is based on the concept that blocking the PC binding site and not removing pCRP from circulation may still allow for protein-protein interaction(s) outside of the PC binding site and utilizing the B-face

of pCRP. However, this is a theoretical benefit and we cannot provide proof for this and it requires further investigations.
We added additional references describing the interaction of various proteins with the B-face of pCRP which do not involve the PC binding site (line 447ff):

"Proteins known to interact with the B-face of pCRP and/or pCRP* include the fibrinogen-like domain of M-ficolin, FH-like protein-1, and human neutrophils (Buchta et al, 1987; Okemefuna et al, 2010a; Zhang et al, 2011). Furthermore, there is evidence that the B-face of pCRP binds to proteins whose secondary structure is predominantly β-sheet (e.g., Amyloid-β1-38), as well as misfolded or aggregated proteins; and that this interaction is independent of the PC binding pocket and located in the vicinity of the interfaces between the monomers in the CRP pentamer (Singh et al, 2009; Hammond et al, 2010)."

Line 411: Why is the "oral route" mentioned? This is a step too far for C10M as long as there is no evidence for it.

Answer:
We agree with the Referee's to be more cautious with our perspective.
We do have data demonstrating the administration of derivatives of the tool compound C10M via intravenous, oral and subcutaneous route (confidential unpublished data). However, due to ongoing patent work we are unable to provide this data in detail in the manuscript. We understand that this answer might be not satisfying and therefore changed the wording in our manuscript towards a more general tone and state the limitations of C10M for prospective oral drug use (line 494ff):

"Generally, small molecular weight drugs can be tailored for oral administration. An oral anti-CRP compound could complement the pre-existing therapy options of either ASO, which would be administered intravenously as typical biologics, or the clinically proven, highly effective CRP-apheresis, which has been tested in hospitalized patients. Given the potential ability to administer small-molecule inhibitors via the oral or parenteral route, in addition to having an immediate onset, we assume C10M suitable to complement these existing CRP targeting therapies. However, based on physiochemical properties particularly the presence of a charged phosphonate group, we anticipate that C10M would be poorly bioavailable due to inefficient gut permeability. Charged phosphonates and phosphates in particular are functional groups that have to be incorporated in some small molecular inhibitors as they are crucial for targeting certain biological processes. As such, many chemical strategies are available to develop prodrugs or delivery systems for phosphonates that mask charged groups during absorption, but are unmasked during first pass liver metabolism; a promising strategy that could be applied to compounds like C10M (Wiemer, 2020)."

Line 414-416: One would like to see a neutral discussion of other approaches. If potential disadvantages of CRP apheresis are to be discussed, then please also mention the advantages, such as practically no side effects and large amounts of CRP are removed from the body within hours instead of being blocked and still in the organism.

Answer:

We apologize for the rather one-sided discussion of formidable approaches like the apheresis of CRP.
We understand the hereby described approach as a complementary therapeutic treatment option. We changed the wording and now also list the advantages of CRP apheresis, i.e., the high efficacy and specificity, but also describing why a small molecule inhibitor might be an additional therapeutic option by adding two potential limitations of CRP apheresis, that were stated by the authors of this approach (line 511ff):

"Whilst CRP-apheresis is a highly effective and specific treatment option at removing large amounts of CRP acutely and thus highly attractive in the acute/emergency setting, the use of apheresis takes approximately 5 hours and needs repeated runs on successive days depending on the indication and the additional use of anticoagulation, which might be critical in some patients (Torzewski et al, 2022). Therefore, a small-molecule CRP inhibitor is a desirable addition to the established techniques given the potential ease of administration and suitability for acute and chronic indications."

Line 452-476: In this section, curiously, CRP is mentioned in general and no longer the individual conformational variants. This leaves the stringent line of argumentation of the rest of the text.

Answer:
We thank the Referee for pointing this out. Following this remark, we changed the wording accordingly and appreciate the improvement our manuscript underwent thanks to the Referee's thorough reading of our manuscript. Please find the paragraphs following line 580ff revised with a more stringent wording:

"We demonstrate that administration of human pCRP and following dissociation to pCRP*/mCRP accelerates allograft rejection via aggravation of IRI and activation of the innate immune response. In both animal models, we establish C10M's unique benefits in reducing pCRP*/mCRP-mediated tissue damage. In the VCA model the acceleration of acute allograft rejection by dissociation of exogenous human pCRP in previously characterized concentrations is reversed by administration of C10M. In the IRI model, renal function is significantly improved and histological signs of kidney injury are markedly reduced. The deposition of human pCRP*/mCRP in the tissue of renal IRI is significantly reduced after administration of C10M, confirming our *in vitro* findings that pCRP binding to activated cell membranes, which we have demonstrated to be a prerequisite for subsequent tissue deposition in the area of inflammation, is reduced (Thiele *et al*, 2014). For our *in vivo* experiments we utilized rat models. Although rats have abundant pCRP (300 – 600 µg/ml plasma in normal healthy pathogen-free rats), rat CRP is not utilized as an acute phase protein and rat complement is not activated by rat CRP (de Beer *et al*, 1982). This is in contrast to human pCRP*/mCRP that activates both rat and human complement, but not mouse complement (Reifenberg *et al*, 2005). Therefore, rats supplemented with human pCRP are a suitable animal model for CRP research (Braig *et al*, 2017; Thiele *et al*, 2014, 2018; Gill *et al*, 2004; Griselli *et al*, 1999; Pepys *et al*, 2006).
The application of human pCRP in rats is a widely accepted animal model, as it mimics most effects human CRP has in humans in a small animal model. Therefore, it has become particularly valuable in models testing therapeutic strategies (Pepys *et al*, 2006) as it is the only model where the final protein target, human pCRP *in vivo*,

can be tested. However, the application of human pCRP into the rat has limitations based on the xenogenic nature of this approach and may also not fully reflect CRP's pathophysiological role in humans. Therefore, in the future additional validation should be conducted in animal models with endogenous CRP, such as rabbits and pigs (Barrett *et al*, 2002; Sheriff *et al*, 2015), to further preclinical drug development. Another limitation of our study is the limited time period over which the effects of pCRP and its inhibition were assessed. CRP inhibition might have to be timed in a tightly fashion as to not interfere with resolution of inflammation required after the acute inflammatory disease state.

Whilst purified native human pCRP itself is described not to be pro-inflammatory when injected into healthy individuals (Lane *et al*, 2014), earlier studies using recombinant human CRP reported endothelial dysfunction and augmented pro-coagulant responses, which were not attributed to potential LPS contaminations (Bisoendial *et al*, 2005, 2007). Notably, purified native human pCRP injected in healthy rabbits showed a significant blood pressure drop (Bock *et al*, 2020). These partially contradictory findings highlight the need for further research on the direct effects of pCRP.

Importantly, C10M i.v. application was successful in obtaining protective effects in two animal models of severe, localized inflammation. The development of the tool compound C10M as a novel small-molecule inhibitor of human pCRP provides important proof-of-concept that inhibition of pCRP conformational change towards pro-inflammatory isoforms, i.e., pCRP*/mCRP represents a highly effective anti-inflammatory approach and paves the way for future design of pharmacologically tailored anti-inflammatory drugs. However, C10M is not designed to interfere with either mCRP or signaling through Fcγ receptors or lipid rafts. The design of C10M rather aims to inhibit the interactions that result in the formation of pCRP*/mCRP. In conclusion, competitively blocking the PC/PE binding pocket on pCRP is a highly promising and attractive strategy towards reduction of CRP-mediated inflammation. Given the wide range of clinical conditions where pCRP*/mCRP-mediated tissue damage has been demonstrated, the therapeutic targeting of pCRP with small-molecule inhibitors is likely to be of broad clinical relevance and can potentially be used in many diseases driven by inflammation."

Figures:

While it is a good idea to provide figures for lectures with headings describing the result of the experiment, one does not wish to do so for a publication, as these results are (supposed to be) described in the results section, please. Examples:

Fig. 1: C10M reduces binding of pCRP to immobilized PC.

Figure 2: C10M prevents pCRP binding to activated platelets. ... pCRP localizes on platelets, which can be inhibited by C10M. ... Platelets bind less CRP when incubated with C10M...

Figure 3: CRP-dependent expression of pro-inflammatory cytokines is abolished by C10M. ... Addition of C10M to the whole blood samples inhibits the CRP-dependent expression of pro-inflammatory cytokines.

And so on for all Figure legends. Please describe only what is needed to understand the figure.

Answer:

Following the Referee's suggestion, we removed the heading and changed the figure legend titles (listed below) and text accordingly. We acknowledge the concern that a

too far-reaching/interpretative title may bias the readers' interpretation and perception of the results. We thank the Referee for pointing this out.

Figure 1:	Binding studies of tool compound C10M to pCRP *in vitro*.
Figure EV1:	Crystallographic structure of pCRP in complex with tool compound C10M.
Table EV1:	Statistics of crystal structure determination of pCRP in complex with C10M.
Table EV2:	Interactions of C10M and PC with pCRP.
Figure 2:	Studies on pCRP binding to activated platelets in the presence of C10M.
Figure 3:	Studies on CRP-dependent expression of pro-inflammatory cytokines and inhibiting effects of C10M.
Figure 4:	pCRP binding to ADP-activated platelets is inhibited by C10M, reducing expression of adhesion molecules involved in leukocyte diapedesis in endothelial cells and leukocytes, ROS formation and NET formation.
Figure EV2:	Inhibitory effects of C10M on pCRP*/mCRP-induced NETosis and leukocyte-endothelial interaction.
Figure 5:	pCRP*/mCRP-driven exacerbation of renal ischemia/reperfusion injury is reduced by C10M.
Figure 6:	Compound C10M delays CRP-driven transplant rejection in a hindlimb transplantation model.
Figure 7:	Flow cytometry-based analysis of CRP-dependent opsono-phagocytosis of S. pneumoniae, E. coli, and zymosan in the presence of C10M.
Figure EV3:	Flow cytometry-based analysis of phagocytosis of S. pneumoniae, E. coli, and zymosan in the presence of C10M.
Figure 8:	Schematic model of CRP conformational changes and C10M's *in vitro* and *in vivo* anti-inflammatory effects.

Line 1463: what does the 35 mean?

Answer:
We apologize for this mistake. The reference was not transferred correctly by the citation software and stayed as a plain number rather than a citation link. The cited work is "Pharmacokinetic study of the novel phosphocholine derivative 3-dibutylaminopropylphosphonic acid by LC-MS coupling" (Kather et al, J Chromatogr B Analyt Technol Biomed Life Sci, 2021, DOI: 10.1016/j.jchromb.2021.122998).

Line 1468: Why do we see 3 CRP bands? This should be discussed in the results section.

Answer:
We thank the Referee for pointing this out. We assume that this effect was due to inadequate storage of the kidney tissue samples and degradation of the protein as shown before, e.g., in Fig 3B (see below, red arrow) by Taylor and van den Berg (Taylor & van den Berg, 2007). We understand the irritation of the referee and therefore ask to use the implemented Western blot in the revised version of our manuscript (Fig 3B, please find below), which was produced using fresh samples and show the uncropped Western blots in the Appendix.

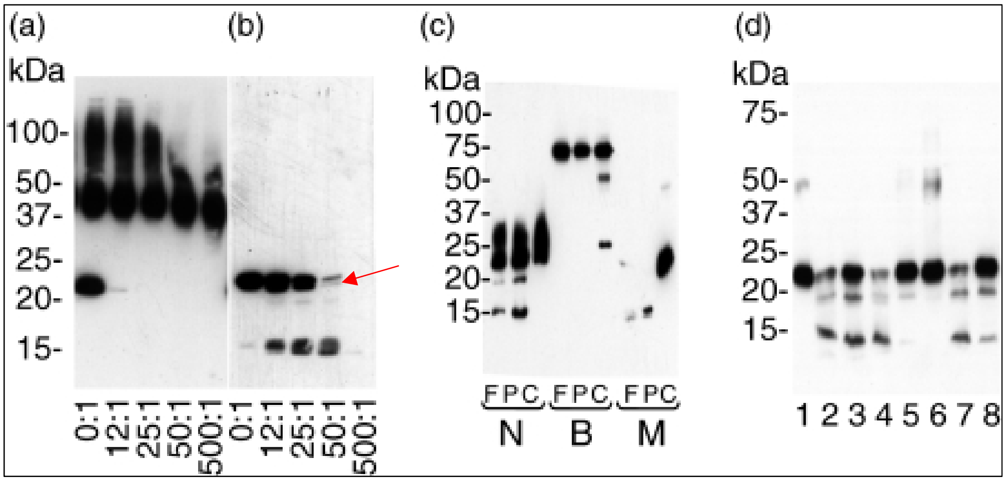

Figure 3B from "Taylor and van den Berg, Structural and functional comparison of native pentameric, denatured monomeric and biotinylated C-reactive protein, Immunology (2007)".

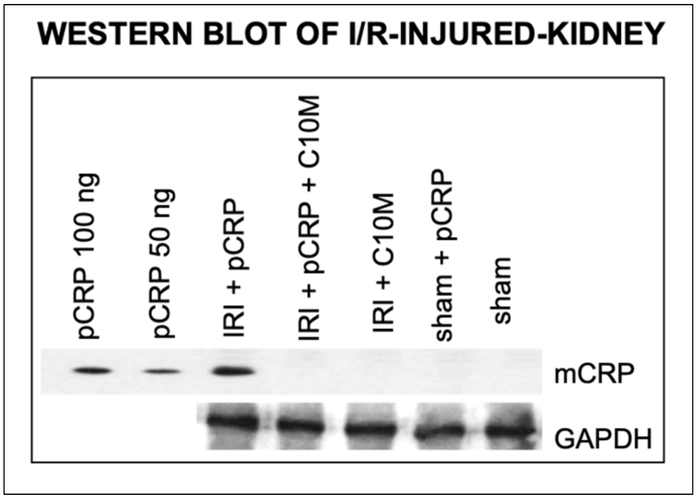

Figure 5: pCRP*/mCRP-driven exacerbation of renal ischemia/reperfusion injury is reduced by C10M.

D Tissue lysates of rat kidney were separated on SDS-PAGE and total CRP was identified with anti-CRP antibody. A band at the size of mCRP (~23 kDa) was detected in kidneys subjected to IRI and pCRP, but not in animals treated additionally with C10M. The household gene protein GAPDH served as a control for loading equal amounts of protein. 50 and 100 ng human pCRP, respectively, served as a positive control. Representative results are shown for replicated assays (n=3).

Line 1489: it is not explained in the figure what exactly the control is?

Answer:
Thank you for pointing this out. We clarified this now in our figure legend:

"Tissue lysates of rat kidney were separated on SDS-PAGE and total CRP was identified with anti-CRP antibody. A band at the size of mCRP (~23 kDa) was detected in kidneys subjected to IRI and pCRP, but not in animals treated additionally with C10M. The household gene protein GAPDH served as a control for loading equal amounts of protein. 50 and 100 ng human pCRP, respectively, served as a positive control. Representative results are shown for replicated assays (n=3)."

Line 1543: of the PC groups of microvesicles, only the LPC derived PC groups can bind to the B-face of pCRP. The reader may not get the impression, that all PC groups are accessible.

Answer:
We thank the Referee for raising this concern. We understand that the referee was concerned that the wording in our previous manuscript draft would give the wrong impression that all PC groups are accessible, and that the second sentence of the referee might contain a typo and the "not" might be superfluous. We hope we understood the Referee correctly. In response, we changed the text accordingly to clarify this point as following (line 1746ff):

"Exposed phospholipids (PL) in activated cell membranes (e.g., endothelial cells or platelets) or microvesicles contain PC head groups derived from LPC and PE head groups derived from lysophosphatidylethanolamine (LPE), which bind to the PC/PE binding sites on the B-face of pCRP thereby anchoring pCRP to the membrane surface."

Figure 8: Which CRP conformation is expected on the vesicles in the upper left scheme? To me it looks like pCRP.

Answer:
The referee is correct and we are grateful for the thorough review of all figures. In the previous figure this detail was not illustrated correctly. We have corrected this now in the new Figure 8. In the upper left scheme of Figure 8 we are illustrating the first step in the destabilization process of CRP, i.e., it is pCRP that we are showing in this scheme. As we describe in the Figure 8 legend, the B-face of pCRP can now bind to the exposed PC/PE head groups of bioactive lipids present on activated endothelial cells and activated platelets, and also on the surface of microvesicles released by damaged cells. Once pCRP binds (as shown in the scheme), destabilization/dissociation starts and subsequently pCRP* is formed and ultimately mCRP is formed. We changed the graphic accordingly (see below) and hope that the new graphical abstract gives a more precise idea of the underlying processes. Again, thanks for pointing this out.

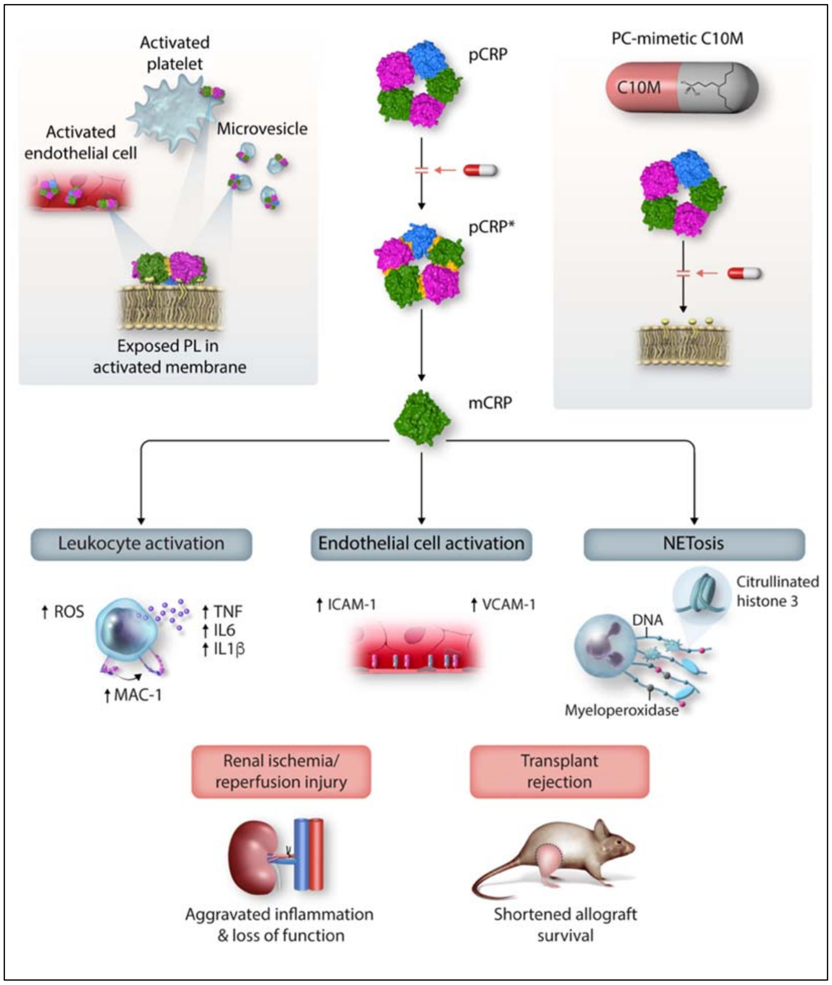

Figure 8: Schematic model of CRP conformational changes and C10M's *in vitro* and *in vivo* anti-inflammatory effects. Exposed phospholipids (PL) in activated cell membranes (e.g., endothelial cells or platelets) or microvesicles contain PC head groups derived from LPC and PE head groups derived from lysophosphatidylethanolamine (LPE), which bind to the PC/PE binding sites on the B-face of pCRP thereby anchoring pCRP to the membrane surface. Once bound to the activated membrane, pCRP dissociates into pCRP* and ultimately mCRP. This pro-inflammatory conformational change results in the activation of leukocytes and endothelial cells as well as NETosis and contributes substantially to renal ischemia/reperfusion injury (IRI) and transplant rejection. Administration of C10M prevents the conformational change of pCRP towards pCRP*/mCRP, the consequent pro-inflammatory cellular effects and reduces renal IRI and transplant rejection.

Referee #2 (Comments on Novelty/Model System for Author):

This manuscript reports on the development and functional characterization of C10M, a phosphocholine-mimicking compound that inhibit formation of pCRP*/mCRP, which mediate the potent pro-inflammatory activities associated with CRP. The experiments are well-designed, properly controlled and powered, and the main conclusions are adequately supported by experimental data. The manuscript is of interest, but the authors need to address some specific points to increase the quality of the work.

> Answer:
> We would like to thank the referee for the thorough assessment of our manuscript and the overall positive review. We appreciate the highly constructive suggestions, which helped to increase the quality of the manuscript. Please find detailed answers to the Referee's requests below.

While overall the model systems are appropriate, functional adhesion assays or assessment of NET formation in more depth could enhance the value the of the study.

> Answer:
> We thank the referee for this suggestion. We have added a more depth description of our results and included the quantification of leukocyte-endothelial cell-interaction and pCRP*/mCRP induced NETosis. This novel data was added to Fig 4 and Expanded View 4, respectively.

Referee #2 (Remarks for Author):

The manuscript by Zeller and collaborators reports development of a phosphocholine-mimicking compound, compound C10M ((3-dibutylamino-propyl) phosphonic acid) that functions as a monovalent inhibitor of structural rearrangements in pCRP. The study combines X-ray crystallography, in vitro experiments and experiments in rat models of renal ischemia-reperfusion and graft rejection. The authors report that C10M binds to the PC/PE binding pocket of pCRP, and inhibits pCRP*/mCRP-evoked activation of monocytes, neutrophils and endothelial cells. Furthermore, treatment with C10M significantly attenuated CRP-mediated renal ischemia-reperfusion-induced and allograft rejection of hindlimb transplantation-associated tissue injury. The findings are of interest but the authors need to address some specific points to increase the quality of the work.

Major Comments.

1. The authors refer to CRP "activating" mechanism. Since native pCRP is known to play important roles in host defense (e.g. opsonization of pathogens, activation of complement, inhibition of leukocyte adherence to the endothelium), "activation" is not a prerequisite for exerting biological functions. Indeed, "activation" appears to refer to changes in CRP conformation, leading to formation of pCRP* and then dissociation to mCRP. Is there a more precise way of describing "activation" (e.g. replacing with "conformational changes" or something similar)?

Answer:
We thank the referee for pointing this out. The term "activation" may indeed be misleading as it may provide the false perception of pCRP being inert. Accordingly, we changed the title to a more precise wording:

"A novel phosphocholine-mimetic inhibits a pro-inflammatory conformational change of C-reactive protein"

2. What was the rationale for using pCRP at various concentrations (ranging from 25-100 ug/ml) in the various experiments? It is unclear how these concentrations were chosen for the specific assays. 100 ug/ml appears to be a rather high concentration. Likewise, some information will be required on how C10M concentrations and in vivo doses were chosen.

Answer:
We thank the referee for this important question. The higher concentrations (100 µg/ml) are supposed to reflect CRP levels in inflammatory, infectious settings in patients. They were used in the opsono-phagocytosis assays which have been previously described (Mold *et al*, 2001).
The CRP concentrations used in the *in vivo* assays (25 µg/ml serum) were chosen to be in line with earlier publications of our group (Thiele *et al*, 2014; Braig *et al*, 2017; Thiele *et al*, 2018). The plasma levels were confirmed by measurements after i.p. administration of human pCRP. The *in vivo* doses of C10M were chosen based on *in vitro* effects of C10M and were used in the molar ratio of C10M/pCRP demonstrating significant reduction of pCRP*/mCRP-induced activation in the monocyte binding assays.
The concentrations used in the *in vitro* assays were 25 µg/ml and 50 µg/ml, respectively, since these concentrations showed significant effects in the respective assay. These concentrations also reflect plasma concentrations that are commonly seen in patients with inflammatory diseases. Since the goal of each independent assay was to show an inhibitory effect of a significant increase of a pro-inflammatory measurement by pCRP*/mCRP-stimulation, the concentrations varied to achieve the significant pro-inflammatory effects. However, all used concentrations are within the

CRP levels seen in acute phase reactions (Pepys *et al*, 1978). We state this now in our discussion (line 524ff, and 561ff):

"Further, we adjusted the initial pCRP concentration in the *in vitro* assays (25 µg and 50 µg per ml) so that we were able to show a significant increase in leukocyte activation (CD11b expression, ROS generation, expression of pro-inflammatory cytokines) and endothelial cell activation (ICAM-1 and VCAM-1 expression, leukocyte adhesion), respectively."

and

"To validate the effect of C10M *in vivo*, we used two distinct animal models of inflammation. Firstly, we exploited an established renal IRI model in rats (Thiele *et al*, 2018). In this model, the administration of human pCRP at previously described concentrations (25 µg/ml serum) demonstrated an aggravated inflammatory response to IRI (Thiele *et al*, 2014; Braig *et al*, 2017; Thiele *et al*, 2018). We demonstrated that the dissociation of human pCRP to pCRP*/mCRP leads to enhanced leukocyte activation, tissue infiltration and generation of ROS resulting in aggravation of tissue injury (Thiele *et al*, 2018). This model represents an established model to investigate the anti-inflammatory properties of C10M as IRI represents the prototypic, sterile inflammatory setting that results in increased tissue damage."

3. Previous studies showed that monocyte recruitment is essential for timely resolution of inflammation. How could then C10M inhibition of pCRP*/mCRP-induced monocyte adhesion be reconciled with anti-inflammatory action?

Answer:
We thank the Referee for addressing this important issue. We do not really have enough data to exclude that C10M influences the timely resolution of inflammation. We are now acknowledging this in the discussion (line 607ff):

"Another limitation of our study is the limited time period over which the effects of pCRP and its inhibition were assessed. CRP inhibition might have to be timed in a tightly fashion as to not interfere with resolution of inflammation required after the acute inflammatory disease state."

We agree that the effects of CRP inhibition on chronic inflammation cannot be deduced from our results. It remains elusive what the effects of CRP inhibition on the timely resolution of inflammation are exactly. Further, the results of our study do not address subtype specific effects in the different monocyte subpopulations, i.e., classical and non-classical monocytes. We agree that inflammation is a complex event in which monocytes and their different subtypes may play opposing roles in the specific temporospatial context. Besides, our models reflect largely the initial phase of inflammation and therefore, we do not provide data on CRP and CRP inhibition on chronic inflammation, respectively. However, our animal models suggest that the detrimental impact of CRP dissociation in IRI and allograft rejection in this acute phase is blocked by C10M and beneficial in this context. Therefore, more detailed and long-term studies are needed to answer these questions.
Interestingly from the data we have so far, it appears that the inhibition of the aggravating effects of CRP dissociation seems to target the overshooting inflammation rather than being immunosuppressive in general.
Indeed, we demonstrated in both animal models that the transmigration of CD68 cells is not inhibited in general.

Biphasic monocyte response in IR injury: we understand the demonstrated effects are based on the reduction of transmigrating monocytes in the early phase of monocyte recruitment described to be more inflammatory and deleterious, whereas the second phase, i.e., from day 5-8, is beneficial in the removal of dead cells and wound healing (Nahrendorf *et al*, 2010).

We hypothesize that the therapeutic effect of C10M is limited to the early phase, and hence, does not disrupt the reparative phase of monocyte recruitment.

For the renal I/R-model we saw preserved kidney function compared to a CRP treated rat group, while C10M did not change the kidney function nor the number of transmigrated cells in absence of human CRP (Fig 5E, F, and H). However, again, we cannot deduct from these results that CRP might mediate the resolution of the inflammation in a later phase and its inhibition, therefore, would not be desirable.

Further, it seems that the pCRP*/mCRP effects are more notable in neutrophils than in monocytes (ROS, CD11b expression). This difference might be partly due to the expression of CD16 in neutrophils, an established receptor for mCRP (Heuertz *et al*, 2005). We agree with the referee that the effects of any inhibition cannot be easily deducted from one surrogate, e.g., transmigrated leukocytes, however, the presence of human CRP in both animal models showed a holistic deterioration for both organs (kidney and hindlimb) as analyzed by overall excretion function and allograft survival. The number of transmigrated monocytes in the injured tissue was not significantly different between the IRI group and the IRI + C10M group, but only increased in the CRP treated animals. This is most likely due to an aggravated inflammatory response mediated by CRP (Griselli *et al*, 1999; Gill *et al*, 2004; Thiele *et al*, 2014; Braig *et al*, 2017).

Furthermore, the small molecule inhibitor C10M does not affect the CRP-independent inflammation and monocyte transmigration, which is indeed needed to resolve the state of inflammation, as shown in the VCA transplantation group, which was treated with C10M in the absence of human CRP. Here, the transplant survival was not significantly affected by the inhibitor C10M (Fig 6B, lower panel; see below).

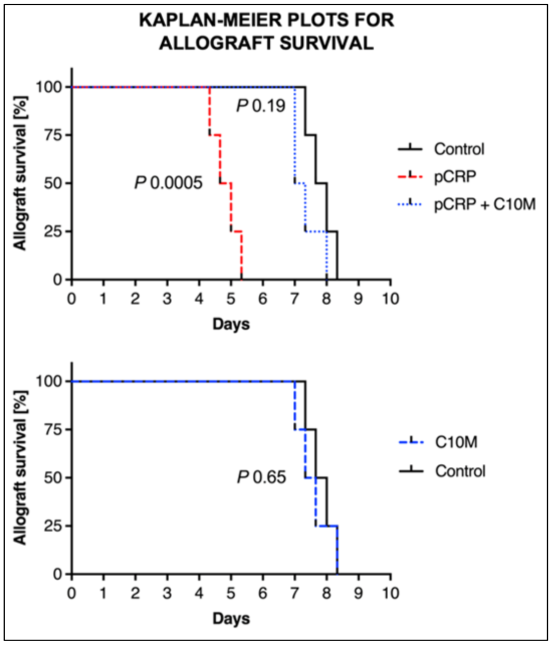

Figure 6: Compound C10M delays CRP-driven transplant rejection in a hindlimb transplantation model.

B Kaplan-Meier plots for control, pCRP, and pCRP+C10M (above) and C10M vs control (bottom) treatment for total allograft survival with and without CRP ± C10M. Kaplan-Meier curves for different treatments were compared by Mantel-Cox log-rank test. Allograft survival was significantly reduced by pCRP administration (P=0.0005, median survival control vs pCRP, 7.8 vs 4.8 days). C10M masks the CRP-accelerated hindlimb rejection (median survival 7.2 days). n=4 per group.

Furthermore, we demonstrated that distinct pro-inflammatory effects of pCRP*/mCRP are inhibited by C10M and pro-inflammatory mediators expressed by monocytes in inflammation, i.e., TNF, IL-1, IL-6 (Freire & Van Dyke, 2013) are significantly reduced (Fig 3E, see below).

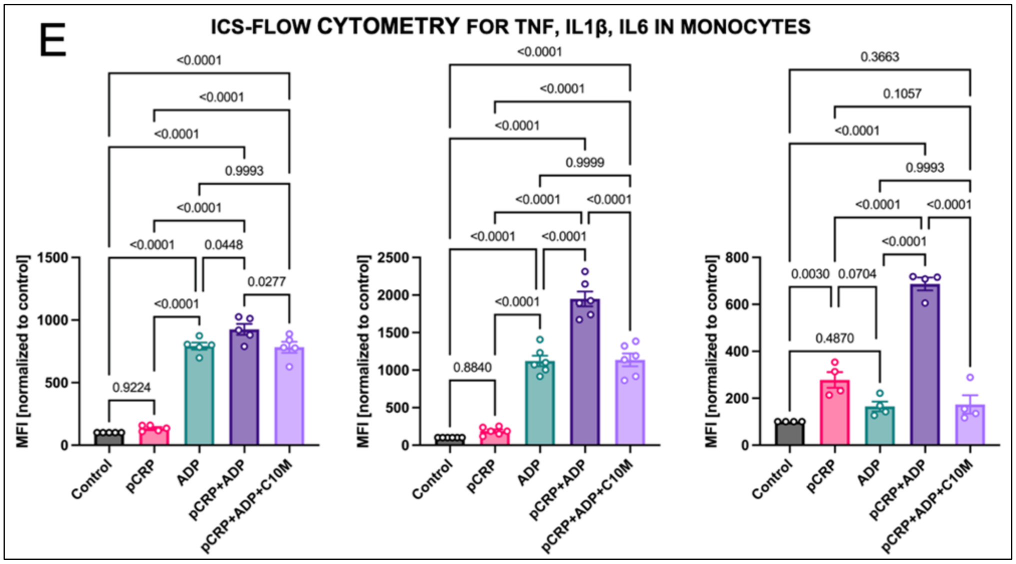

Figure 3: Studies on CRP-dependent expression of pro-inflammatory cytokines and inhibiting effects of C10M.

E Expression of TNF, IL-1β and IL-6 (from left to right) in monocytes analyzed by flow cytometry. Addition of C10M to the whole blood samples inhibits the CRP-dependent expression of pro-inflammatory cytokines. P values were calculated with ANOVA and Tukey's post-hoc test. n=6 (IL-6), n=5 (TNF) and n=4 (IL-1β), respectively, bars indicate mean ± SEM.

4. C10M reduction of NETosis is interesting. However, the biological consequences of this effect cannot be deduced from the results presented for viable NETosis (as opposed to suicidal NETosis) is thought to play protective role when pathogens cannot be phagocytosed. Labeling of Figure S3 may be misleading for it depicts pCRP*/mCRP vs. CRP+C10M.

Answer:
We thank the referee for their comment in regard to the interesting effects of pCRP*/mCRP on NETosis and its potential inhibition by C10M. We agree that the biological relevance of this is not defined and that we need to be cautious in the interpretation of our results. Indeed, this model was used as another *in vitro* readout for pCRP*/mCRP induced inflammation and how blocking the conformational change to pCRP*/mCRP by C10M can alleviate pro-inflammatory CRP effects. In the *in vivo* scenario inflammation is a highly complex, multi-staged process that is almost

impossible to mimic *in vitro* in its whole complexity. We therefore try to avoid overinterpretation of our findings in the revised manuscript. However, following the referee's advice, we went one step further and analyzed the mode of NET formation in the pCRP*/mCRP stimulated neutrophils. In response to the reviewer's comments, we performed a novel set of experiments to elucidate the mode of pCRP*/mCRP induced NETosis in regard to cell viability. In our observations, the pro-inflammatory isoforms of CRP induce suicidal NETosis, which led to consistent loss of cell membrane integrity as demonstrated in Expanded View 4B. However, further investigations are needed to determine the exact pathway of pCRP*/mCRP-induced NETosis. We have now added the following text to our manuscript (line 364ff):

"A novel finding of our investigation into the mode of action of CRP-regulated inflammation is that pCRP*/mCRP induces formation of neutrophil extracellular traps (NETs) (Fig 4H and EV2A), a process called NETosis. NETosis is a mediator of inflammation, a key event that modulates tissue and organ damage (Cahilog *et al*, 2020). Our finding that C10M reduces pCRP*/mCRP-induced NETosis further highlights the relevance of CRP regulation as an important immune checkpoint and the therapeutic potential of C10M. pCRP*/mCRP induces suicidal NETosis after three hours of stimulation (Fig EV2B), a mode of NETosis where the membrane integrity is lost during the process (Yipp & Kubes, 2013). However, further analyses of the exact pathway of pCRP*/mCRP-induced NETosis has to be conducted to ultimately elucidate the underlying mechanism."

According to the formatting style of EMBO Molecular Medicine, we omitted the supplementary figures. Confocal immunofluorescence microscopy images showing pCRP*/mCRP-induced NETosis have been transferred to Expanded View 4 (please find below).

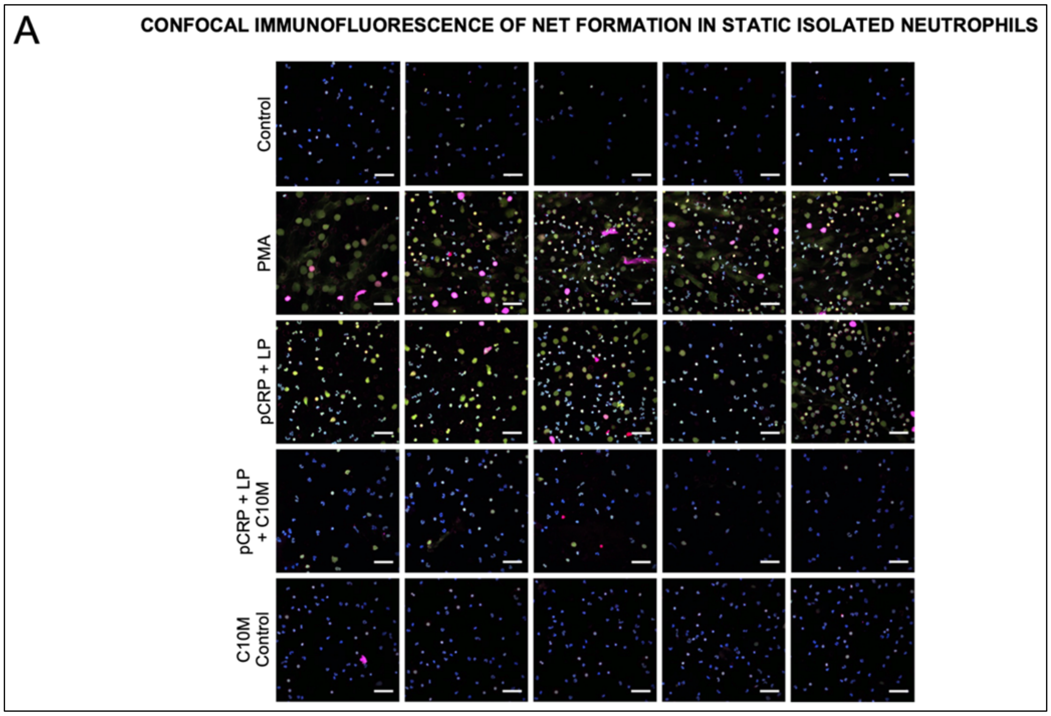

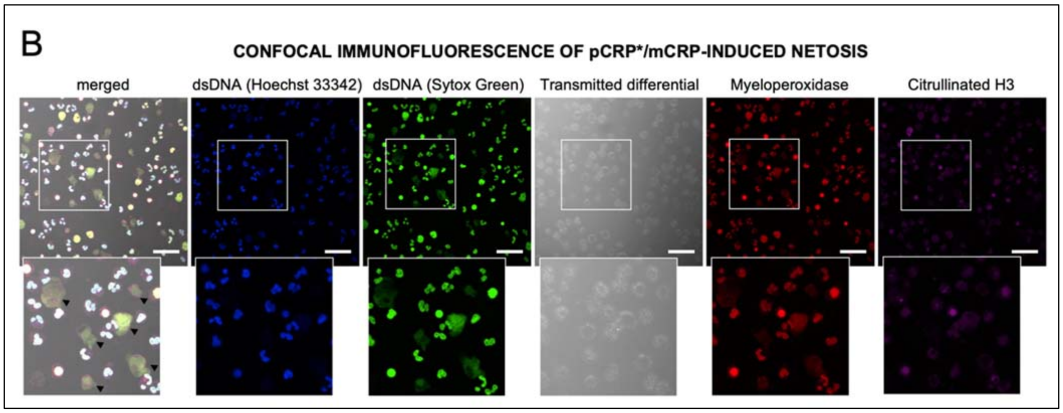

Figure EV2: Inhibitory effects of C10M on pCRP*/mCRP-induced NETosis and leukocyte-endothelial interaction

A Representative confocal immunofluorescence images of isolated human neutrophils as summarized in Fig. 4H. Cells were stained with anti-dsDNA dyes Hoechst 33342 and Sytox Green, and anti-MPO and anti-citH3 antibodies. Shown are uncropped images of merged channels and at 40x magnification. Scale bars indicate 50 µm.

B Representative single-channel confocal immunofluorescence images of pCRP*/mCRP-induced NETosis. Cells demonstrating NETs in the pCRP*/mCRP-stimulated group showed disrupted cell membranes as visible in the transmitted differential and merged channel (black arrow heads), indicating a suicidal mode of NETosis. Scale bars indicate 50 µm.

5. Can C10M interfere with mCRP binding or signaling through CD16 or lipid rafts?

Answer:
We thank the Referee for this important question. We have tested inhibitory effects of C10M on mCRP-dependent activation of leukocytes with urea-dissociated mCRP but found no significant effects. This is in line with our previous finding that fully unfolded mCRP loses the PC binding site and thereby a C10M binding would not be effective ((Braig *et al*, 2017); Supplementary data Figure 9 (please find attached below)).
C10M binds directly to pCRP, thereby preventing the formation of mCRP – so in this respect, C10M does interfere with mCRP binding. By preventing the formation of mCRP, C10M also interferes with any process involving mCRP.
In response to the Referee's question, we have now added the following text to the manuscript (line 626f):

"However, C10M is not designed to interfere with either mCRP or signaling through Fcγ receptors or lipid rafts. The design of C10M aims to inhibit the interactions that result in the formation of pCRP*/mCRP."

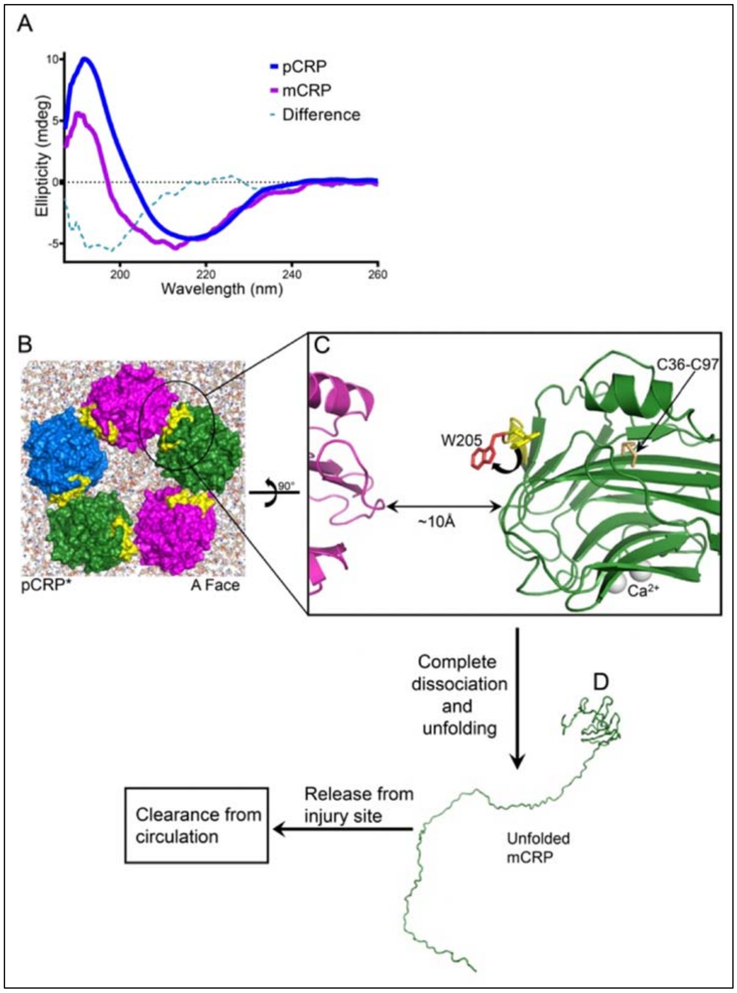

Supplementary Figure 9: Comparison of the UV CD spectra of pCRP and mCRP and model illustrating pCRP* dissociation to mCRP. (A) UV CD spectra of pCRP (solid blue line) and mCRP (solid purple line) in 20 mM sodium phosphate buffer (pH 7.5) and 25 mM NaCl. The difference between the two curves (Δ = mCRP – pCRP; dashed light blue line) indicates the secondary structure difference between these two CRP isoforms. (B) Model of pentameric pCRP* on a cholesterol:POPC lipid bilayer, same view and coloring as Fig. 7E. The antigenic neo-epitope for the mCRP-specific antibody anti-mCRP-9C9, i.e. CRP residues 199-206 (yellow), is exposed at the inter-subunit interface. (C) Close up view of the inter-subunit interface after a rotation of +90o about the X-axis. The lipid bilayer is not shown. pCRP* is shown in cartoon format and the distance between the protein backbone of adjacent CRP subunits is indicated. At this distance the electrostatic interactions (salt bridges and hydrogen bonds) between amino acid side-chains on adjacent CRP subunits are extremely weak (distance > 4 Å). Three main options are available to pCRP*: (i) maintain the current structure (eg. for C1q binding, Fig. 7G and H); (ii) reform the native pCRP structure (Fig. 7C and D) and (iii) completely dissociate. The location of the intra-subunit disulfide bond (C36-C97, cream colored sticks) and the two Ca2+ ions (grey spheres) are indicated. Trp205 (W205) in the neo-epitope (yellow) is now able to flip out becoming solvent exposed (red W205) and the neo-epitope is accessible for interaction with antibodies anti-mCRP 9C9 or anti-mCRP 3H12. (D) Reducing or Ca2+-deficient conditions will result in the breaking of the intra-subunit disulfide bond or removal of the Ca2+ ions, respectively, either of which will result in unfolding of the CRP monomer. The unfolded mCRP is then released from the site of injury, or inflammation, for clearance. (Braig *et al*, 2017)

Minor Points

1. Are reductions in ICAM-1 and VCAM-1 expression sufficient to prevent neutrophil or monocyte adherence to HUVEC?

Answer:
We thank the referee for raising this important question. In order to address this question and to gain more pathomechanistic insight we performed a novel set of experiments. We investigated the leukocyte binding to HUVEC incubated with different CRP isoforms and inhibited the dissociation of pCRP to pCRP*/mCRP using C10M.

We investigated two different leukocytes, THP-1 cells (monocyte cell line) and neutrophils (isolated from human whole blood) in their capability to adhere to HUVEC monolayers treated with the same conditions previously shown to effectively reduce the pCRP*/mCRP induced expression of ICAM-1 and VCAM-1. To exclude platelet dependent effects on the cell-cell interaction (Rossaint *et al*, 2018), we employed generic LPC:PC liposomes rather than activated platelets to serve as activated surfaces for CRP dissociation (Braig *et al*, 2017). After the indicated period of incubation, cells were washed and analyzed by fluorescence microscopy. Images were analyzed using an automated cell counting tool in ImageJ as previously described (Grishagin, 2015). We provide a detailed description of the method in the methods section (line 1496).
Intriguingly, C10M demonstrated clear reduced expression of cell adhesion molecules as measured by a significant decrease of both leukocyte subtypes bound to the endothelial cell monolayer (Expanded View Fig 4C, D, E). We added the additional data in Expanded View Fig 4C, D, and E (see below) and in line 299ff:

"Intriguingly, the pCRP*/mCRP-induced upregulation of ICAM-1 and VCAM-1 in HUVEC was reduced by C10M sufficiently to suppress an aggravated adhesion of the monocytic cell line THP-1 (Fig EV2C) and neutrophils (Fig EV2D) to the monolayer."

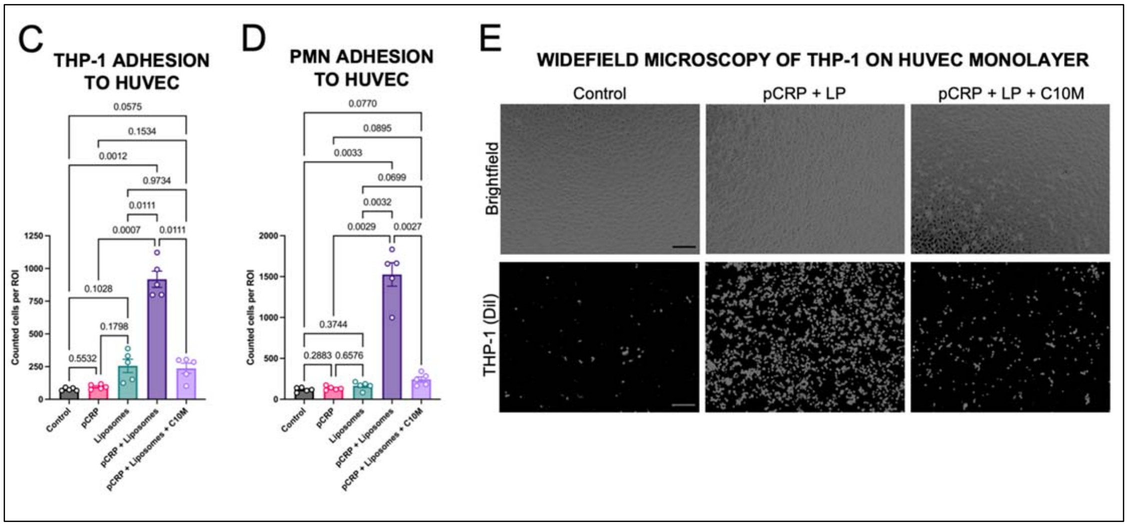

Figure EV2: Inhibitory effects of C10M on pCRP*/mCRP-induced NETosis and leukocyte-endothelial interaction

C,D,E Leukocyte adhesion on endothelial cells. HUVEC monolayers treated with different isoforms of CRP as described for Fig. 4B and C were incubated with

fluorescently-labeled THP-1 cells (C,E) and neutrophils isolated from human whole blood (PMN, D) for 30 min. After incubation, the monolayer was washed and adherent cells were fixed. THP-1 cell and neutrophil binding to HUVEC monolayers were then evaluated by automated cell counting in five non-overlapping ROIs at 10x magnification as demonstrated for THP-1 in (E). Scale bars indicate 100 µm. Graph shows mean ± SEM. P values were calculated with ANOVA and Tukey's post-hoc test. n=5 for THP-1 and PMN, respectively.

2. The title to Figure 4 lists ROS and NET formation, while these measurements are not depicted on the figure.

Answer:
We apologize for this oversight. The respective data was shown in the supplementary files but has now been transferred to Fig 4. In the revised version of our manuscript, we show ROS and NET data in Fig 4 F,G, and H and EV2.

3. Figure 5B. Please explain what the three curves illustrate. Also for panel D, please indicate the number of experiments for which representative results are shown.

Answer:
We apologize for the ambiguity of the graph. For clarification we added (1) a precise panel title as required for EMBO Molecular Medicine, i.e., "mass spectrometry of renal excretion of C10M", and (2) a graphical demonstration of the experiment showing a Wistar rat injected with C10M (blue syringe) and the following urine excretion and measurement by MS (Fig 5B; see below). The experiment was performed for three animals (n=3), each represented by one curve. We now state (line 1634ff):

"Detection of C10M by mass spectrometry in rat urine. Renal excretion of C10M detected by mass spectrometry in three rats (n=3) intravenously injected with C10M. Urine samples were collected at the indicated timepoints. 80% of the applied C10M mass was excreted after 90 min (Kather et al, 2021)."

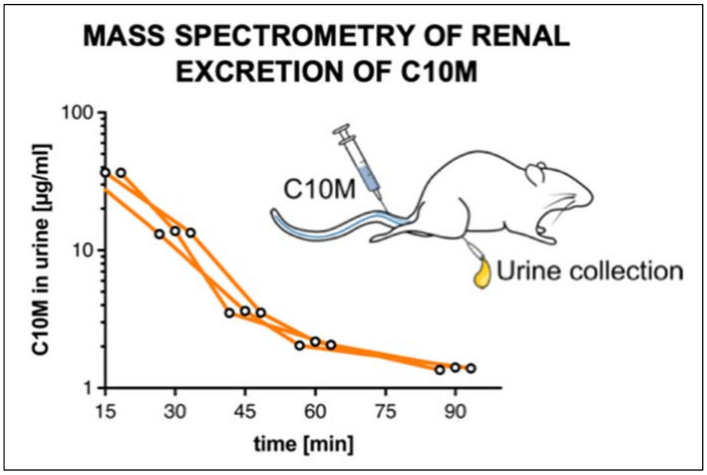

Figure 5: pCRP*/mCRP-driven exacerbation of renal ischemia/reperfusion injury is reduced by C10M.

B Detection of C10M by mass spectrometry in rat urine. Renal excretion of C10M detected by mass spectrometry in three rats (n=3) intravenously injected with C10M. Urine samples were collected at the indicated timepoints. 80% of the applied C10M mass was excreted after 90 min (Kather et al, 2021).

4. Figure 8 depicts molecules/mechanisms (e.g. histone, elastase or chatepsin G) that are not addressed in the manuscript. Does C10M prevent pCRP* formation or binding of pCRP to cell membrane (as shown on the right panel)?

Answer:
We thank the referee for bringing this to our attention. We changed the graphical abstract accordingly (see below).
By occupying the PC/PE binding pockets on the B-face of each CRP monomer in pCRP, C10M prevents pCRP from binding to damaged or activated cells, thereby stopping the subsequent dissociation/destabilization of pCRP to pCRP*, and ultimately the dissociation of pCRP* to mCRP.
As requested for the revisions of our manuscript, we added further data showing the pCRP*/mCRP dependent NETosis (Fig. 4H) and added citrullinated histone 3 and MPO detection (confocal immunofluorescence microscopy). We deleted cathepsin G since we are not showing this in our assays.

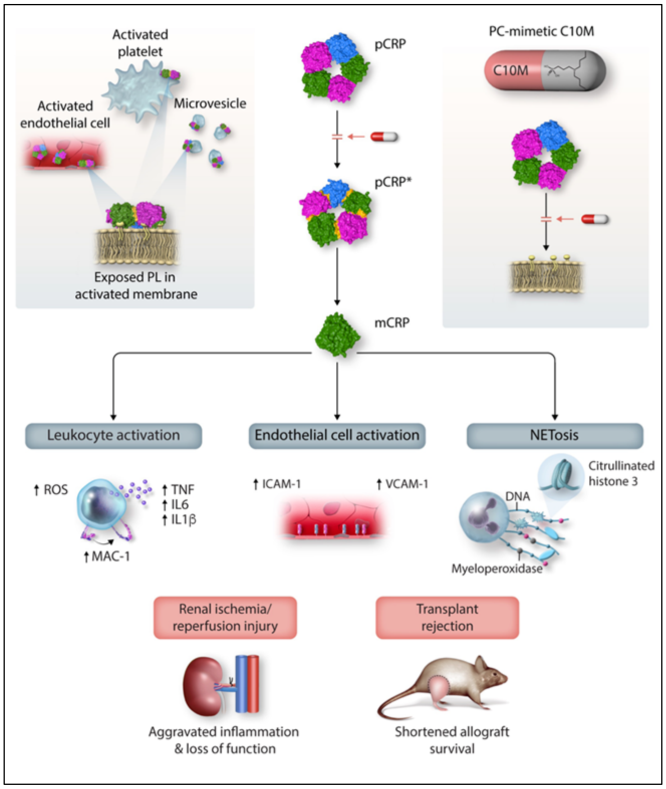

Figure 8: Schematic model of CRP conformational changes and C10M's *in vitro* and *in vivo* anti-inflammatory effects. Exposed phospholipids (PL) in activated cell membranes (e.g., endothelial cells or platelets) or microvesicles contain PC head groups derived from LPC and PE head groups derived from

lysophosphatidylethanolamine (LPE), which bind to the PC/PE binding sites on the B-face of pCRP thereby anchoring pCRP to the membrane surface. Once bound to the activated membrane, pCRP dissociates into pCRP* and ultimately mCRP. This pro-inflammatory conformational change results in the activation of leukocytes and endothelial cells as well as NETosis and contributes substantially to renal ischemia/reperfusion injury (IRI) and transplant rejection. Administration of C10M prevents the conformational change of pCRP towards pCRP*/mCRP, the consequent pro-inflammatory cellular effects and reduces renal IRI and transplant rejection.

5. Lines 260 and 757. "HUVEC cell" is superfluous for "C" in HUVEC stands for "cell or cells".

Answer:
We apologize for this oversight. We have changed this accordingly in both lines (line 282f and 942, respectively) and throughout the text. Furthermore, we took care to use the correct term in the added experiments for the revised manuscript.

“pCRP on platelets and microvesicles, respectively, bind to HUVEC monolayers (Fig 4A, first row and Appendix S2A).”

“HUVEC interaction with platelet-bound CRP”

Referee #3 (Comments on Novelty/Model System for Author):

The generated small molecule C10M able to inhibit the active forms of CRP is of great interest. The in vitro experiments are convincing of its efficacy but I have concerns about the suitability of the in vivo models that are based in the injection of human active CRP; a somehow artificial model.

Referee #3 (Remarks for Author):

This study reports the generation of a phosphocholine-mimicking tool compound (C10M) able to inhibit CRP activation. The design is based in the mechanism of activation of CRP identified by the authors in a previous study. It has the advantage of binding to inactive pentameric CRP (pCRP) preventing its interaction with bioactive lipids on activated cell membranes and its subsequent activation. X-ray crystallography studies show binding of C10M to pCRP. In vitro and in vivo studies reveal the ability of C10M to inhibit CRP-driven exacerbation of inflammatory responses. The in vitro models are convincing, but I have some concerns about the in vivo models.

Answer:
We thank the referee for the overall positive review and their interest in the topic. We acknowledge the referee's concern regarding the rat models in combination with the application of human CRP. We prepared detailed responses below to explain the rationale behind the rat *in vivo* model applied in our manuscript.

Major points:
1. Figs 4D, 4E and S3B: the effect of C10 M is not statistically significant. Is is possible to use pre-activated platelets and analyzed other markers?

Answer:
We thank the referee for this question. Indeed, we realized that we made a mistake and presented the same graph for monocytes and neutrophils in Fig 4D and 4E. We have now corrected this (see below).

The inhibitory effects of C10M on neutrophil activation measured by CD11b expression were significant. However, the trend seen in CD11b expression in the monocyte population was not significant. By showing the whole scale of results rather than only showing significant data (Fig 4D, 4E) we were aiming to provide a detailed picture of CRP effects and how C10M can potentially be used as an anti-inflammatory agent.
For the measurement of ROS generation in neutrophils and monocytes, we excluded one outlier (after we performed an outlier test) and re-did the experiment. C10M significantly reduced ROS generation in both neutrophils and monocytes (Fig 4F, 4G).

Additionally, we have now added a quantitative read-out of pCRP*/mCRP-induced NETosis in our manuscript rather than the qualitative assay we provided in the previous submission. Here, we found C10M to significantly reduce the dissociation of pCRP and the following induction of NETosis in human isolated neutrophils (Fig 4H). We utilized the dissociation of pCRP on LPC:PC liposomes to minimize the effects on NETosis of other CRP-independent factors, theoretically expressed by activated platelets for example (Caudrillier *et al*, 2012). Phorbol 12-myristate 13-acetate (PMA) was utilized as the most commonly used positive control. Intriguingly, the CRP induced NET formation was limited to the pro-inflammatory isoforms pCRP*/mCRP generated on LPC:PC liposomes. This effect based on the binding of pCRP to activated membranes and the subsequential dissociation towards pCRP*/mCRP, as previously described (Braig *et al*, 2017), was sufficiently inhibited by C10M.

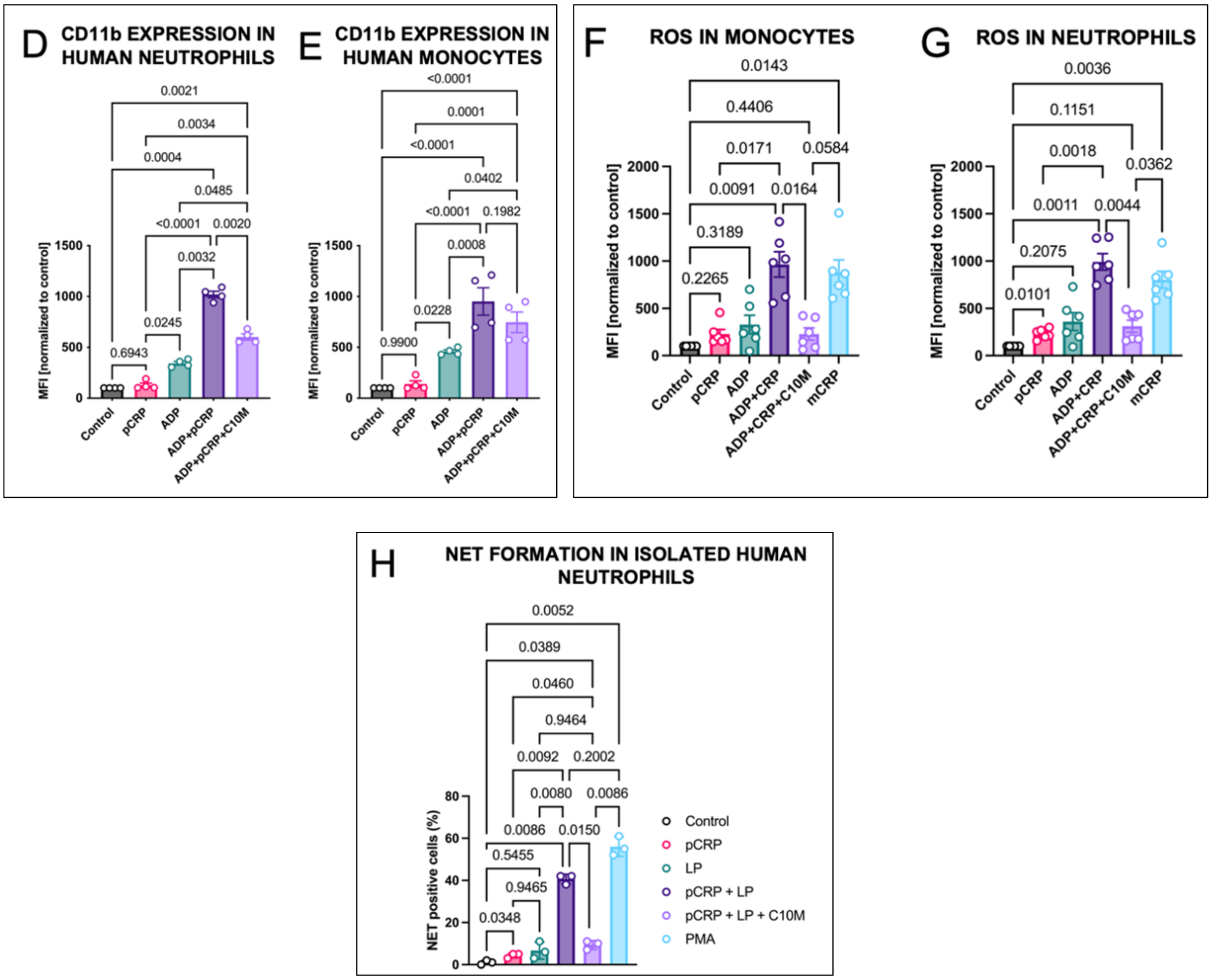

Figure 4: pCRP binding to ADP-activated platelets is inhibited by C10M, reducing expression of adhesion molecules involved in leukocyte diapedesis in endothelial cells and leukocytes, ROS formation and NET formation.

D,E Expression of integrin subunit αM (CD11b) in neutrophils (D) and CD14+ monocytes (E) was accessed by flow cytometry as described previously (Kiefer et al, 2021). Human whole blood was incubated with 25 µg/ml pCRP, 20 µM ADP and C10M (molar ratio 1:100, pCRP:C10M), respectively. CD11b expression was analyzed by flow cytometry in neutrophils (CD16+, SSC high) and monocytes (CD14+, SSC low). Shown are scatter plots of MFI results in flow cytometry with results normalized to control, mean ± SEM. P values were calculated with ANOVA and Tukey's post-hoc test. n=6.

F,G ROS generation in whole blood detected in CD14+ monocytes (F) and neutrophils (G) by redox-indicator dihydroethidium (DHE; 10 µg/ml). Blood samples incubated for 3 hours at 37 °C, 5% CO_2 with 50 µg/ml pCRP and mCRP, 20 µM ADP and C10M (molar ratio 1:100, pCRP:C10M), respectively. Control was left unstimulated and mCRP served as positive control (Thiele et al, 2018). Cells were washed after red blood cell lysis and analyzed by flow cytometry. Shown are MFI results with results normalized to control, mean ± SEM. P values were calculated with ANOVA and Tukey's post-hoc test. n=5.

H pCRP*/mCRP dependent NETosis in isolated human neutrophils detected by confocal immunofluorescence microscopy. Isolated neutrophils incubated for 3 hours at 37 °C, 5% CO_2 with 100 µg/ml pCRP with and without PC:LPC liposomes and C10M (molar ratio 1:100, pCRP:C10M), respectively. Control was left unstimulated and 100 nM PMA served as positive control. Cells were washed, fixed and stained and analyzed by confocal microscopy. Results are given as a ratio of NETing cells/all cells per ROI, with mean ± SEM. P values were calculated with ANOVA and Tukey's post-hoc test. n=3.

2. Figs 5 and 6: It can be read in the discussion that the rat model is ideal to investigate the role of human CRP to inflammation and the impact of C10M. However, I think injection of human pCRP is an artificial model, since it is observed the impact of C10M in the exacerbated pathology caused by human pCRP. Is is possible to use alternative animal models to analyze the impact of C10M in endogenously activated CRP?

Answer:
We thank the Referee for raising this important question. Whilst we acknowledge the concerns regarding the potentially artificial nature of the rat model and the request for a model in which endogenous CRP plays a more central role, we wish to highlight that the use of rats as an animal model in combination with human CRP (the actual target of our compounds is indeed tested in this approach) is nonetheless well established, and commonly utilized, in the field of CRP biology by other groups (Griselli *et al*, 1999; Gill *et al*, 2004; Pepys *et al*, 2006) and our group (Thiele *et al*, 2014; Braig *et al*, 2017). The rational for this model is that endogenous CRP is not an acute phase reactant in rats (de Beer *et al*, 1982), however, human pCRP*/mCRP activates complement and leukocytes in rats similar to its *in vivo* role in human. This model is indeed unique in that it mimics the *in vivo* in human situation and the actual pharmacological target for our tool compound, namely human pCRP, is present in this animal model. Thus, the rat supplemented with human pCRP is a well-established animal model in CRP research.
We agree, that a model with endogenous CRP could potentially provide further proof of concept. Nevertheless, using human CRP has the critical advantage that we are testing our tool compound in its effect against the final target for human therapy. Furthermore, having the ability to include a control without human CRP provides a unique control experiment that controls for the effects of endogenous rat CRP. We believe that our current *in vivo* models and data, which are based on several years of work towards establishing these models, especially the quite unique and clinically relevant hindlimb transplant model in rats, is very strong and well-controlled.

However, to incorporate the referee's concern in our manuscript, we now suggest further animal models that could be used to provide proof of concept addressing the different CRP roles in different animal models. Accommodating the referee's concern, we also do not use the term "ideal" for the currently used rat model with exogenous CRP application. We now state both the shortcomings and advantages of the rat model in our manuscript (line 599ff):

"The application of human pCRP in rats is a widely accepted animal model, as it mimics most effects human CRP has in humans in a small animal model. Therefore, it has become particularly valuable in models testing therapeutic strategies (Pepys *et al*, 2006) as it is the only model where the final protein target, human pCRP *in vivo*, can be tested. However, the application of human pCRP into the rat has limitations based on the xenogenic nature of this approach and may also not fully reflect CRP's pathophysiological role in humans. Therefore, in the future additional validation

should be conducted in animal models with endogenous CRP, such as rabbits and pigs (Barrett *et al*, 2002; Sheriff *et al*, 2015), to further preclinical drug development."

3. Fig. 7: This experiment is important to evaluate if C10M only inhibit the pathological effects of CRP. Therefore, the effects of C10M in pCRP-induced phagocytosis needs to be studied. In addition, this in vitro result may be further validated by analyzing the effects of C10M in in vivo infection experiments.

Answer:
To broaden and strengthen the relevance of our data, following this reviewer's advice we performed a novel set of experiments investigating the phagocytosis of more pathogens, Escherichia coli and zymosan (a glucan and ligand found on the surface of fungi and commonly used in phagocytosis assays). We have added the results in Figure 7 and Figure EV3.
We now present data on the effects of C10M on phagocytosis for *S. pneumoniae*, *E. coli*, and zymosan, respectively, in the presence and in the absence of pCRP, in a flow cytometry-based phagocytosis assay (line 380ff):

"pCRP led to an increase in phagocytosis of *S. pneumoniae* serotype 27 in monocytes and neutrophils (Fig 7A and G), which was reduced by addition of C10M (Fig 7D and J). Intriguingly, serotype 27 expresses PC as part of its capsule (Bennett & Bishop, 1977a, 1977b). Therefore, the capsule makes serotype 27 a target for pCRP opsonization (Edwards *et al*, 1982; Pilishvili *et al*, 2010) and a competitor for the C10M binding. However, there was no significant increase of phagocytosed *E. coli*, a gram-negative bacterium, in the presence of pCRP nor were effects of C10M measured in monocytes or neutrophils (Fig 7 B,E and H,K), respectively. The effects of pCRP opsonization on phagocytosis of *E. coli* as a gram-negative bacterium play a minor role (Mold *et al*, 1982), and the innate immune response is based on toll-like receptors and CRP-independent activation and opsonization by the complement system (C1q, mannose-binding lectin, C4b, C3b/iC3b) and immunoglobins (Van Dijk *et al*, 1979; Newman & Mikus, 1985). For zymosan, we detected an increase (albeit non-significant except for 20 min in monocytes) of phagocytosis in both leukocyte subtypes after pCRP opsonization (Fig 7C and I) and incubation with pCRP and C10M (Fig 7F and L), and the phagocytosis in the absence of pCRP was not affected by C10M for any pathogen target or leukocyte, respectively (Fig EV3). This suggests that the protective capacities of the innate immune system remain largely maintained in CRP targeting therapy, which is in line with earlier reports of clinical studies utilizing a CRP lowering approach as CRP ASO and CRP-apheresis (Warren *et al*, 2015; Torzewski *et al*, 2020; Ries *et al*, 2021; Torzewski *et al*, 2021; Ringel *et al*, 2021; Schumann *et al*, 2021; Torzewski *et al*, 2022)."

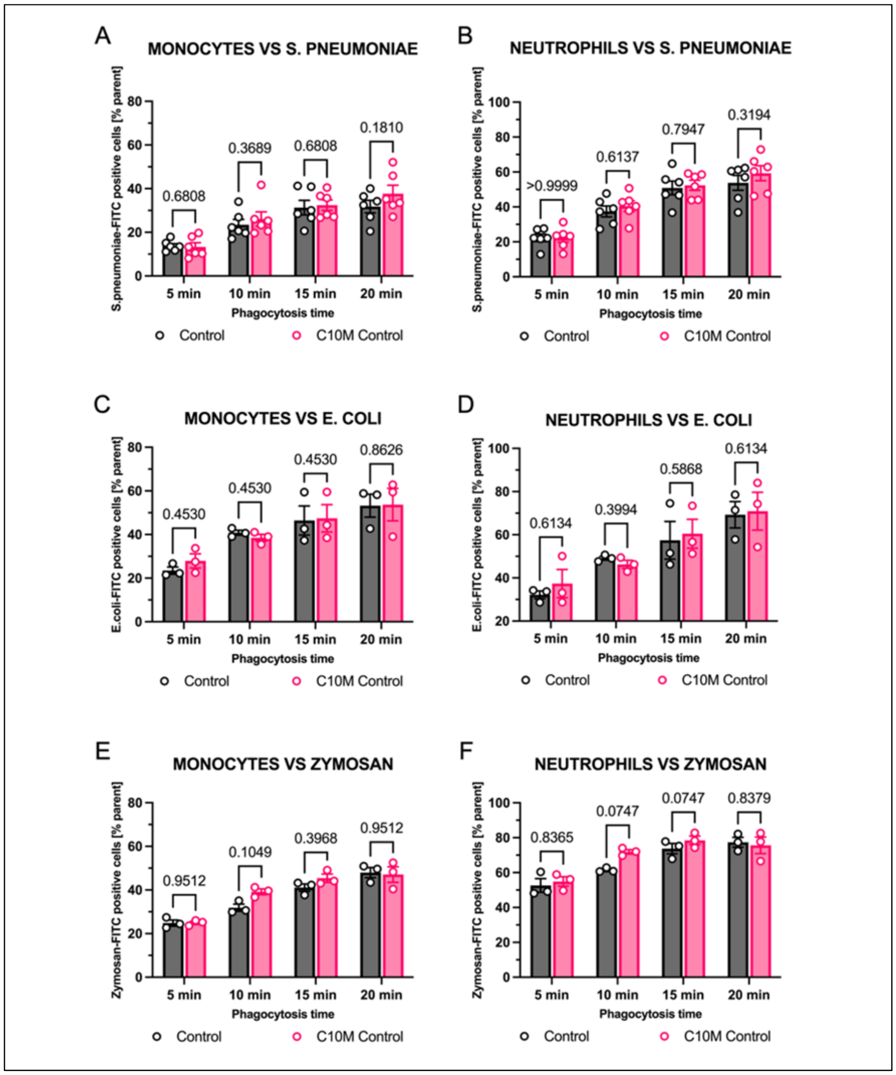

Figure EV3: Flow cytometry-based analysis of phagocytosis of S. pneumoniae, E. coli, and zymosan in the presence and the absence of C10M.

A,B Phagocytosis of heat-killed and FITC-labeled *S. pneumoniae* by monocytes (A) and neutrophils (B) was analyzed with or without C10M (in equal concentrations used as demonstrated in Fig 7). Scatter plot shows phagocytic index (percentage of target positive cells of subtype / all cells of subtype) of an untreated control versus C10M treated cells after 5, 10, 15, and 20 min, respectively. Mean ± SEM are indicated.

C,D Phagocytosis assay for heat-killed and FITC-labeled *E. coli* by monocytes (C) and neutrophils (D)

E,F Phagocytosis of the yeast cell wall ligand zymosan (*Saccharomyces cerevisiae*) by monocytes (E) and neutrophils (F). Mean ± SEM are indicated.

Statistical analysis for all assays shown was performed using multiple matched t-tests. n=3 and 6. Precise p-values are given.

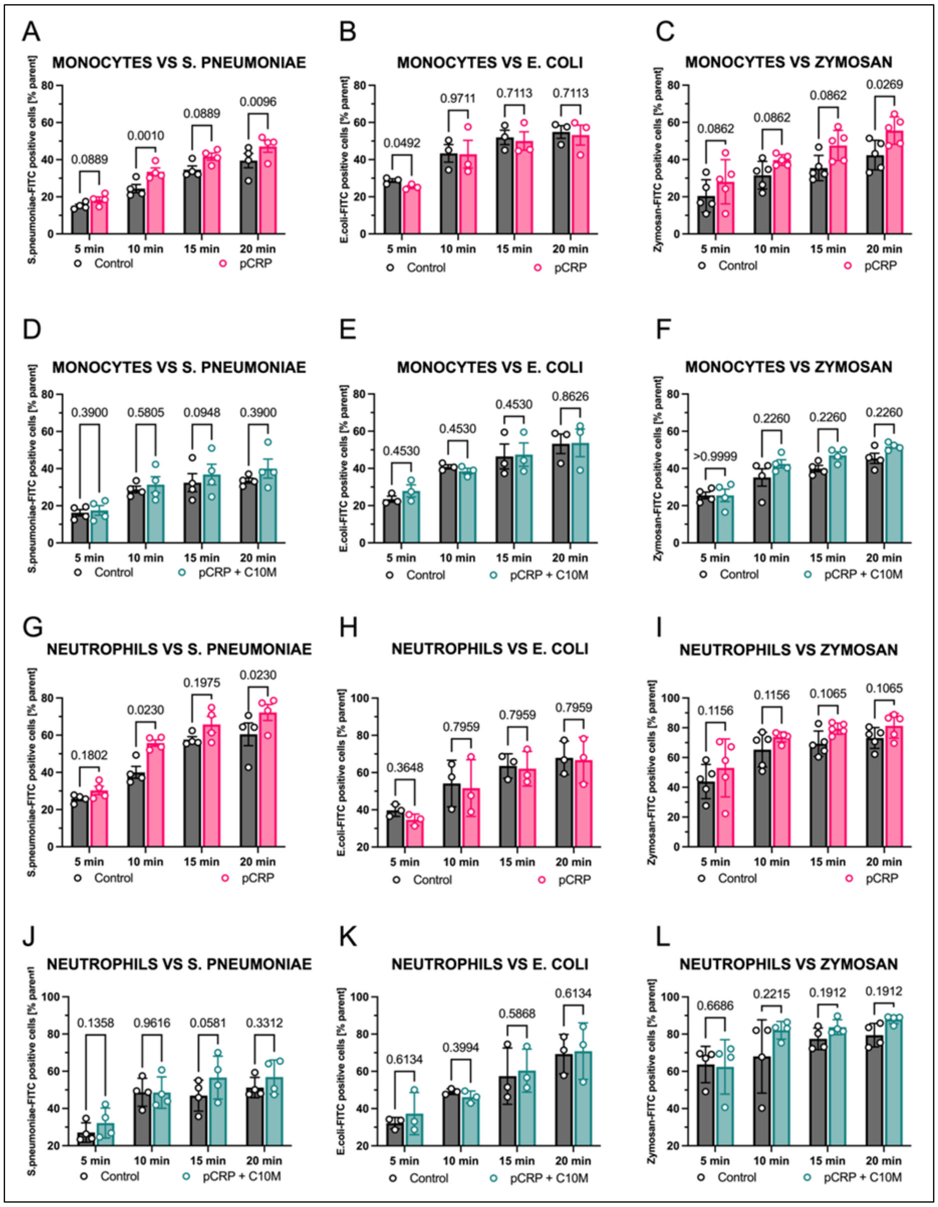

Figure 7: Flow cytometry-based analysis of CRP-dependent opsono-phagocytosis of *S. pneumoniae*, *E. coli*, and zymosan in the presence of C10M.

A Phagocytosis of pCRP-opsonized, heat-killed and FITC-labeled *S. pneumoniae* by human monocytes serves as exemplary flow cytometry-based phagocytosis assay. Bar chart shows phagocytic index (percentage of target positive cells of subtype / all cells of subtype) of un-opsonized (control, dark-grey) and pCRP-opsonized targets (red) after 5, 10, 15, and 20 min, respectively.

B Phagocytosis of pCRP-opsonized, heat-killed and FITC-labeled *E. coli* by human monocytes serves as a second exemplary flow cytometry-based phagocytosis assay. Bar chart shows phagocytic index as described above of un-opsonized (control, dark-grey) and pCRP-opsonized targets (red) after 5, 10, 15, and 20 min, respectively.

C Phagocytosis of pCRP-opsonized, heat-treated and FITC-labeled zymosan by human monocytes serves as a third exemplary flow cytometry-based phagocytosis assay. Bar chart shows phagocytic index as described above of un-opsonized (control, dark-grey) and CRP-opsonized targets (red) after 5, 10, 15, and 20 min, respectively.

D Experiments described in (A) were repeated but with targets incubated with pCRP (100 µg/ml) and C10M (1:100 molar ratio) for 30 min, 37 °C. Bar chart shows phagocytic index of *S. pneumoniae*-positive monocytes for un-opsonized (control, dark-grey) and targets incubated with pCRP and C10M (green) after 5, 10, 15, and 20 min, respectively.

E Experiments described in (B) were repeated but with targets incubated with pCRP (100 µg/ml) and C10M (1:100 molar ratio) for 30 min, 37 °C. Bar chart shows phagocytic index of *E. coli*-positive monocytes for un-opsonized (control, dark-grey) and targets incubated with pCRP and C10M (green) after 5, 10, 15, and 20 min, respectively.

F Experiments described in (C) were repeated but with targets incubated with pCRP (100 µg/ml) and C10M (1:100 molar ratio) for 30 min, 37 °C. Bar chart shows phagocytic index of zymosan-positive monocytes for un-opsonized (control, dark-grey) and targets incubated with pCRP and C10M (green) after 5, 10, 15, and 20 min, respectively.

G Phagocytosis of pCRP-opsonized, heat-killed and FITC-labeled *S. pneumoniae* by human neutrophils. The same blood samples described in (A) were analyzed for the phagocytic index of *S. pneumoniae* by human neutrophils by flow cytometry. Bar chart shows phagocytic index of un-opsonized (control, dark-grey) and pCRP-opsonized targets (red) after 5, 10, 15, and 20 min, respectively.

H Phagocytosis of pCRP-opsonized, heat-killed and FITC-labeled *E. coli* by human neutrophils. The same blood samples described in (B) were analyzed for the phagocytic index of *S. pneumoniae* by human neutrophils by flow cytometry. Bar chart shows phagocytic index of un-opsonized (control, dark-grey) and pCRP-opsonized targets (red) after 5, 10, 15, and 20 min, respectively.

I Phagocytosis of pCRP-opsonized, heat-killed and FITC-labeled zymosan by human neutrophils. The same blood samples described in (C) were analyzed for the phagocytic index of *S. pneumoniae* by human neutrophils. Bar chart shows phagocytic index of un-opsonized (control, dark-grey) and pCRP-opsonized targets (red) after 5, 10, 15, and 20 min, respectively.

J Phagocytosis of pCRP+C10M-treated *S. pneumoniae* by human neutrophils. Results from experiments described in (D) were analyzed for the phagocytic index of *S. pneumoniae* by human neutrophils by flow cytometry. Bar chart shows phagocytic index of *S. pneumoniae*-positive neutrophils for un-opsonized (control, dark-grey) and targets incubated with pCRP and C10M (green) after 5, 10, 15, and 20 min, respectively.

K Phagocytosis of pCRP+C10M-treated *E. coli* by human neutrophils. Results from experiments described in (E) were analyzed for the phagocytic index of *S. pneumoniae* by human neutrophils by flow cytometry. Bar chart shows phagocytic index of *E. coli*-positive neutrophils for un-opsonized (control, dark-grey) and targets incubated with pCRP and C10M (green) after 5, 10, 15, and 20 min, respectively.

L Phagocytosis of pCRP+C10M-treated zymosan by human neutrophils. Results from experiments described in (F) were analyzed for the phagocytic index of *S. pneumoniae* by human neutrophils by flow cytometry. Bar chart shows phagocytic

index of zymosan-positive neutrophils for un-opsonized (control, dark-grey) and targets incubated with pCRP and C10M (green) after 5, 10, 15, and 20 min, respectively.

Data information: Bar graphs with individual experiments (A-L) show mean ± SD of the phagocytic index (percentage of target positive cells of subtype / all cells of subtype) of un-opsonized (control, dark-grey), CRP-opsonized targets (red), and CRP-opsonized targets in the presence of C10M after 5, 10, 15, and 20 min, respectively. *P* values were calculated using multiple matched t-tests. n=3-5. Precise p-values are given.

We agree that additional *in vivo* experiments, including toxicology studies and the mentioned *in vivo* infection studies, validating the use of the small molecule inhibitor tool compound C10M, have to be performed to advance this compound to clinical translation, including clinical trials. We are currently applying for funding for this expensive and time-intensive package of preclinical experiments and a phase 1 first in man study.
Also, the referee raises an important question regarding the theoretical risk of immunosuppression by therapies lowering CRP levels. We understand that there is no indication that the reduction of CRP levels achieved may compromise the immune response. Two elegant approaches in lowering CRP levels, i.e., CRP antisense oligonucleotides (ASO) and CRP apheresis, have shown no immunosuppressive effects, especially in regard to bacterial infections (line 122ff):

"However, one theoretical concern of CRP reducing therapies is the potential risk of immunosuppression, especially in bacterial infections and sepsis, given CRP's well-described role in the innate immune response. Intriguingly, in the ISIS studies, evaluating the reduction of endotoxin-induced increase in CRP levels by pre-treatment with ASO, healthy endotoxin-challenged volunteers did not demonstrate any evidence of a compromised immune response and the ASO was well tolerated with no serious adverse events (Noveck *et al*, 2014). Moreover, in the phase II clinical trial, patients treated with a CRP ASO demonstrated no increase in infections compared to control patients (Warren *et al*, 2015). Other CRP lowering approaches, such as apheresis, have also shown that the therapeutic targeting of CRP does not appear to increase the risk of infection (Torzewski *et al*, 2020; Ries *et al*, 2021; Torzewski *et al*, 2021; Ringel *et al*, 2021; Schumann *et al*, 2021; Torzewski *et al*, 2022). Therefore, therapeutic targeting of CRP is an attractive anti-inflammatory therapy approach and different strategies have been developed and are being tested with promising initial results."

Minor points:
1. All figures: labeling each panel will facilitate the interpretations of the results w/o the need of the legend that it is quite tedious.

Answer:
We thank the referee for this helpful advice. We added a title/heading for each panel of each figure in uppercase letters. We thereby hope to facilitate the interpretations of our figures.

2. Fig. 5C: label the CRP deposition

Answer:
We apologize for this oversight. We have added arrows for Fig 5C as suggested to help to identify the deposition of the human CRP in the injured tubules of the rat kidney.

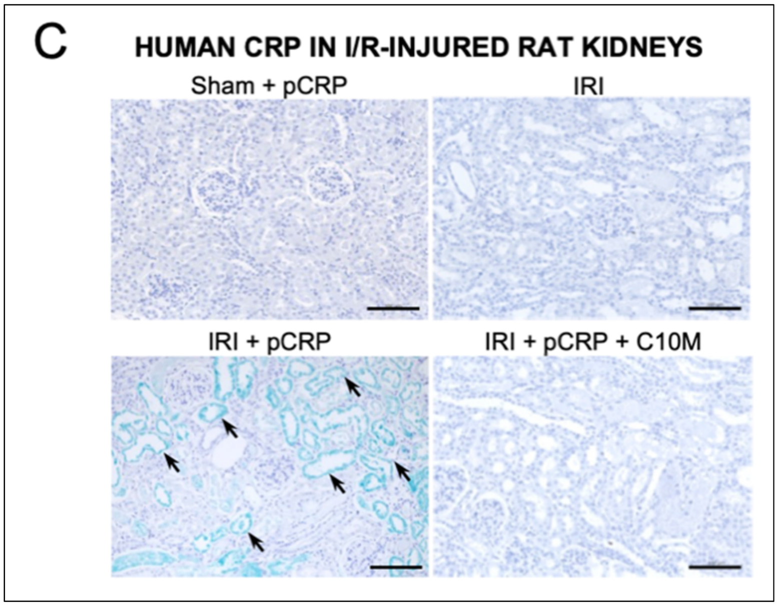

Figure 5: pCRP*/mCRP-driven exacerbation of renal ischemia/reperfusion injury is reduced by C10M.

C Immunohistochemistry of rat kidneys subjected to IRI and i.p. pCRP application revealed distinct staining by anti-pCRP*/mCRP-9C9 antibody (green, arrows). C10M reduces the deposition of total CRP in the impaired tissue. No deposits in the non-ischemic tissue (sham). Exemplary stainings out of at least three are shown. Scale bars, 100 µm.

3. Fig. S3C: the labeling used is confusing and does not match the legend. The effects of C10M in NDET formation needs quantification.

Answer:
We apologize for this oversight. The respective data for pCRP*/mCRP-dependent NETosis was shown in the supplementary files but has now been transferred to Fig 4H and EV2. In the revised version of our manuscript, we give a quantitative analysis of pCRP*/mCRP-dependent NETosis and the inhibitory effects of C10M. In this additional assay, we quantified NETs from isolated human neutrophils by confocal

microscopy after stimulation for three hours with pCRP. The neo-epitope expressing forms pCRP/mCRP and PMA (positive control) are also shown. LPC:PC liposomes served as activated bio-membranes as previously described by our group (Braig *et al*, 2017) and others (Ji *et al*, 2007; Wang *et al*, 2012). The binding of pCRP to LPC:PC liposomes was successfully inhibited by C10M (see below).

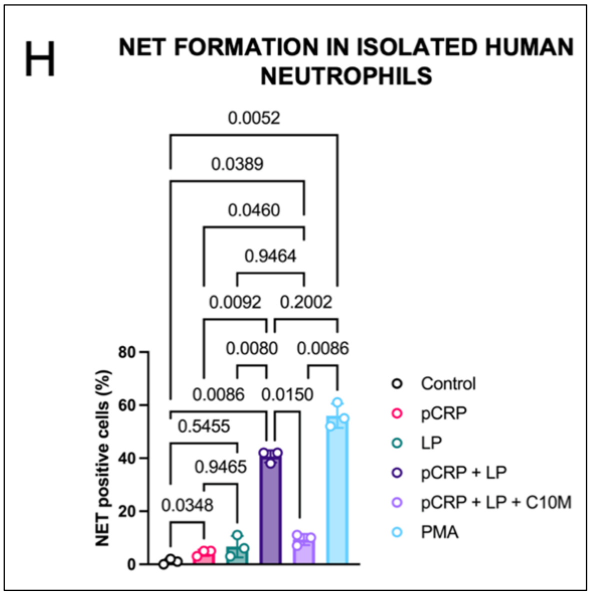

Figure 4: pCRP binding to ADP-activated platelets is inhibited by C10M, reducing expression of adhesion molecules involved in leukocyte diapedesis in endothelial cells and leukocytes, ROS formation and NET formation.
H pCRP*/mCRP dependent NETosis in isolated human neutrophils detected by confocal immunofluorescence microscopy. Isolated neutrophils incubated for 3 hours at 37 °C, 5% CO_2 with 100 µg/ml pCRP with and without PC:LPC liposomes and C10M (molar ratio 1:100, pCRP:C10M), respectively. Control was left unstimulated and 100 nM PMA served as positive control. Cells were washed, fixed and stained and analyzed by confocal microscopy. Results are given as a ratio of NETing cells/all cells per ROI, with mean ± SEM. P values were calculated with ANOVA and Tukey's post-hoc test. n=3.

4. Discussion: is there any advantage of C10M over bis-PC? Please, discuss.

Answer:
We thank the Referee for this question and the opportunity to extend the discussion comparing the monovalent C10M and the bivalent bis-PC (please find a figure above in response to Referee 1).

C10M binds to the PC/PE binding pocket on the B-face of each CRP monomer in the non-inflammatory pCRP as illustrated in Fig 1 and Fig EV1. Once C10M is bound, CRP continues to circulate as pCRP as the ability to bind to damaged cells/membranes is then blocked. The A-face of the pentamer remains available to interact with other proteins, e.g., Fcγ receptors. Any function, independent of the PC binding pocket, that the B-face of pCRP has should be retained, e.g., binding to β-sheets, such as those found in β-amyloid plaques and other misfolded proteins, and binding the fibrinogen-like domain (FBG) of M-ficolin, FH-like protein-1, and human neutrophils. Such PC-independent binding has been described before for non-native

CRP formed by acidic pH, which exposes a hidden ligand binding site for non-PC ligands. This was suggested to enable CRP to bind to denatured and aggregated proteins, e.g., the binding of oxidized LDL (Hammond *et al*, 2010). Importantly, complement factor H (FH) binds to different binding sites on pCRP (Okemefuna *et al*, 2010b).

However, C10M is blocking only the PC binding site, leaving the remainder of the B-face of pCRP otherwise accessible. We hypothesize that other, potentially beneficial, additional protein-protein interaction can still take place. We discuss our approach in the revised manuscript now in more detail (line 443ff):

"The monovalent compound strategy allows both the A-face and B-face, with the exception of the PC binding pocket, of the CRP pentamer to remain available to interacting partner proteins and their associated functions. In contrast, only the A-face of the CRP pentamer is available for interaction when a bivalent compound like bis-PC is utilized. Proteins known to interact with the B-face of pCRP and/or pCRP* include the fibrinogen-like domain of M-ficolin, FH-like protein-1, and human neutrophils (Buchta et al, 1987; Okemefuna et al, 2010a; Zhang et al, 2011). Furthermore, there is evidence that the B-face of pCRP binds to proteins whose secondary structure is predominantly β-sheet (e.g., Amyloid-β1-38), as well as misfolded or aggregated proteins; and that this interaction is independent of the PC binding pocket and located in the vicinity of the interfaces between the monomers in the CRP pentamer (Singh et al, 2009; Hammond et al, 2010)."

Further, ongoing research in our labs suggests an interaction of misfolded proteins with the B-face of pCRP without utilization of the PC binding site (unpublished confidential data, illustrated by some of our modelling results below). Intriguingly, pentameric CRP and SAP were described to function as extracellular chaperones, and pCRP appears to interact calcium-independently with β-sheets forming amyloid, showing anti-amyloidogenic behavior (Ozawa *et al*, 2016). The calcium-independent interaction however, indicates an interaction outside the PC binding site.

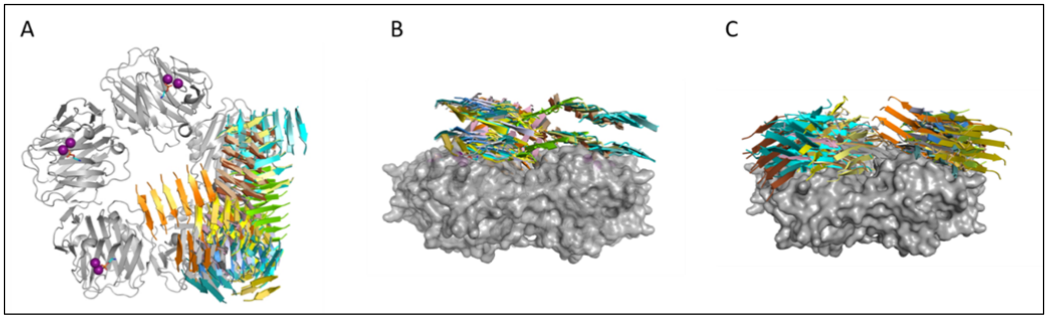

Results of Amyloid-β16-21 (KLVFFA, PDB ID:30W9) and Amyloid-β35-42 (MVGGVVIA, PDB ID:2Y3L) β-sheet blind docking with two monomers from the PE:pCRP complex (PDB ID: 3L2Y). In panel **A**, pCRP is shown as a grey cartoon, Ca2+ as purple spheres and bound PE as colored sticks. **B**, Side view of the PE:pCRP complex, clearly shows the orientation of the Amyloid-β16-21 β-sheets. **C**, Side view of the PE:pCRP complex, showing the orientation of the longer Amyloid-β35-42 β-sheets. In B and C, pCRP is depicted as a grey molecular surface.

However, since we are not showing any proof in this study that these protein interactions are primarily beneficial, we have changed the wording accordingly and

incorporating the Referee's comment, no longer call it advantageous over the previously published approach of bis-PC (line 137ff):

"We set out to design a low molecular weight monovalent tool compound that targets the PC/PE binding pocket on pCRP and thereby has the potential to prevent the formation of the pro-inflammatory pCRP* and mCRP species. Our strategy follows a distinctly different design compared to the previously published bivalent approach, as it allows the CRP pentamer to remain intact and in circulation, with both the A- and B-faces available to interact with ligands and retain associated functions."

References

Barrett TD, Hennan JK, Marks RM & Lucchesi BR (2002) C-Reactive-Protein-Associated Increase in Myocardial Infarct Size After Ischemia/Reperfusion. *J Pharmacol Exp Ther* 303: 1007–1013

de Beer FC, Baltz ML, Munn EA, Feinstein A, Taylor J, Bruton C, Clamp JR & Pepys MB (1982) Isolation and characterization of C-reactive protein and serum amyloid P component in the rat. *Immunology* 45: 55–70

Bennett LG & Bishop CT (1977a) Structure of the type XXVII *Streptococcus pneumoniae* (pneumococcal) capsular polysaccharide. *Can J Chem* 55: 8–16

Bennett LG & Bishop CT (1977b) The pyruvate ketal as a stereospecific immunodeterminant in the type XXVII Streptococcus pneumoniae (pneumococcal) capsular polysaccharide. *Immunochemistry* 14: 693–696

Bisoendial R, Birjmohun R, Keller T, van Leuven S, Levels H, Levi M, Kastelein J & Stroes E (2005) In vivo effects of C-reactive protein (CRP)-infusion into humans. *Circ Res* 97: e115-116

Bisoendial RJ, Kastelein JJP, Peters SLM, Levels JHM, Birjmohun R, Rotmans JI, Hartman D, Meijers JCM, Levi M & Stroes ESG (2007) Effects of CRP infusion on endothelial function and coagulation in normocholesterolemic and hypercholesterolemic subjects. *J Lipid Res* 48: 952–960

Bock C, Vogt B, Mattecka S, Yapici G, Brunner P, Fimpel S, Unger JK & Sheriff A (2020) C-Reactive Protein Causes Blood Pressure Drop in Rabbits and Induces Intracellular Calcium Signaling. *Front Immunol* 11: 1978

Braig D, Nero TL, Koch H-G, Kaiser B, Wang X, Thiele JR, Morton CJ, Zeller J, Kiefer J, Potempa LA, *et al* (2017) Transitional changes in the CRP structure lead to the exposure of proinflammatory binding sites. *Nat Commun* 8: 14188

Buchta R, Pontet M & Fridkin M (1987) Binding of C-reactive protein to human neutrophils. *FEBS Lett* 211: 165–168

Cahilog Z, Zhao H, Wu L, Alam A, Eguchi S, Weng H & Ma D (2020) The Role of Neutrophil NETosis in Organ Injury: Novel Inflammatory Cell Death Mechanisms. *Inflammation* 43: 2021–2032

Caudrillier A, Kessenbrock K, Gilliss BM, Nguyen JX, Marques MB, Monestier M, Toy P, Werb Z & Looney MR (2012) Platelets induce neutrophil extracellular traps in transfusion-related acute lung injury. *J Clin Invest* 122: 2661–2671

Edwards KM, Gewurz H, Lint TF & Mold C (1982) A role for C-reactive protein in the complement-mediated stimulation of human neutrophils by type 27 Streptococcus pneumoniae. *J Immunol Baltim Md 1950* 128: 2493–2496

Esposito F, Matthes H & Schad F (2022) Seven COVID-19 Patients Treated with C-Reactive Protein (CRP) Apheresis. *J Clin Med* 11: 1956

Freire MO & Van Dyke TE (2013) Natural resolution of inflammation. *Periodontol 2000* 63: 149–164

Gill R, Kemp JA, Sabin C & Pepys MB (2004) Human C-reactive protein increases cerebral

infarct size after middle cerebral artery occlusion in adult rats. *J Cereb Blood Flow Metab Off J Int Soc Cereb Blood Flow Metab* 24: 1214–1218
Griselli M, Herbert J, Hutchinson WL, Taylor KM, Sohail M, Krausz T & Pepys MB (1999) C-reactive protein and complement are important mediators of tissue damage in acute myocardial infarction. *J Exp Med* 190: 1733–1740
Grishagin IV (2015) Automatic cell counting with ImageJ. *Anal Biochem* 473: 63–65
Hammond DJ, Singh SK, Thompson JA, Beeler BW, Rusiñol AE, Pangburn MK, Potempa LA & Agrawal A (2010) Identification of acidic pH-dependent ligands of pentameric C-reactive protein. *J Biol Chem* 285: 36235–36244
Heuertz RM, Schneider GP, Potempa LA & Webster RO (2005) Native and modified C-reactive protein bind different receptors on human neutrophils. *Int J Biochem Cell Biol* 37: 320–335
Ji S-R, Wu Y, Zhu L, Potempa LA, Sheng F, Lu W & Zhao J (2007) Cell membranes and liposomes dissociate C-reactive protein (CRP) to form a new, biologically active structural intermediate: mCRP $_{m}$. *FASEB J* 21: 284–294
Kather MG, Zeller J, Plattner D, Breit B, Kreuzaler S, Krippner G, Peter K, Eisenhardt SU & Kammerer B (2021) Pharmacokinetic study of the novel phosphocholine derivative 3-dibutylaminopropylphosphonic acid by LC-MS coupling. *J Chromatogr B* 1186: 122998
Kiefer J, Zeller J, Bogner B, Hörbrand IA, Lang F, Deiss E, Winninger O, Fricke M, Kreuzaler S, Smudde E, *et al* (2021) An Unbiased Flow Cytometry-Based Approach to Assess Subset-Specific Circulating Monocyte Activation and Cytokine Profile in Whole Blood. *Front Immunol* 12: 641224
Lane T, Wassef N, Poole S, Mistry Y, Lachmann HJ, Gillmore JD, Hawkins PN & Pepys MB (2014) Infusion of pharmaceutical-grade natural human C-reactive protein is not proinflammatory in healthy adult human volunteers. *Circ Res* 114: 672–676
McFadyen JD, Kiefer J, Braig D, Loseff-Silver J, Potempa LA, Eisenhardt SU & Peter K (2018) Dissociation of C-Reactive Protein Localizes and Amplifies Inflammation: Evidence for a Direct Biological Role of C-Reactive Protein and Its Conformational Changes. *Front Immunol* 9: 1351
Mold C, Gresham HD & Du Clos TW (2001) Serum Amyloid P Component and C-Reactive Protein Mediate Phagocytosis Through Murine FcγRs. *J Immunol* 166: 1200–1205
Mold C, Rodgers CP, Kaplan RL & Gewurz H (1982) Binding of human C-reactive protein to bacteria. *Infect Immun* 38: 392–395
Nahrendorf M, Pittet MJ & Swirski FK (2010) Monocytes: protagonists of infarct inflammation and repair after myocardial infarction. *Circulation* 121: 2437–2445
Newman SL & Mikus LK (1985) Deposition of C3b and iC3b onto particulate activators of the human complement system. Quantitation with monoclonal antibodies to human C3. *J Exp Med* 161: 1414–1431
Nijmeijer R, Lagrand WK, Baidoshvili A, Lubbers YTP, Hermens WT, Meijer CJLM, Visser CA, Hack CE & Niessen HWM (2002) Secretory type II phospholipase A(2) binds to ischemic myocardium during myocardial infarction in humans. *Cardiovasc Res* 53: 138–146
Nijmeijer R, Willemsen M, Meijer CJLM, Visser CA, Verheijen RH, Gottlieb RA, Hack CE & Niessen HWM (2003) Type II secretory phospholipase A2 binds to ischemic flip-flopped cardiomyocytes and subsequently induces cell death. *Am J Physiol Heart Circ Physiol* 285: H2218-2224
Noveck R, Stroes ESG, Flaim JD, Baker BF, Hughes S, Graham MJ, Crooke RM & Ridker PM (2014) Effects of an antisense oligonucleotide inhibitor of C-reactive protein synthesis on the endotoxin challenge response in healthy human male volunteers. *J Am Heart Assoc* 3: e001084
Okemefuna AI, Nan R, Miller A, Gor J & Perkins SJ (2010a) Complement factor H binds at two independent sites to C-reactive protein in acute phase concentrations. *J Biol Chem* 285: 1053–1065
Okemefuna AI, Nan R, Miller A, Gor J & Perkins SJ (2010b) Complement factor H binds at two independent sites to C-reactive protein in acute phase concentrations. *J Biol Chem* 285: 1053–1065
Ozawa D, Nomura R, Mangione PP, Hasegawa K, Okoshi T, Porcari R, Bellotti V & Naiki H

(2016) Multifaceted anti-amyloidogenic and pro-amyloidogenic effects of C-reactive protein and serum amyloid P component in vitro. *Sci Rep* 6: 29077
Padilla ND, van Vliet AK, Schoots IG, Seron MV, Maas MA, Peltenburg EEP, de Vries A, Niessen HWM, Hack CE & van Gulik TM (2007) C-reactive protein and natural IgM antibodies are activators of complement in a rat model of intestinal ischemia and reperfusion. *Surgery* 142: 722–733
Pepys MB (2021) C-reactive protein predicts outcome in COVID-19: is it also a therapeutic target? *Eur Heart J* 42: 2280–2283
Pepys MB, Dash AC, Markham RE, Thomas HC, Williams BD & Petrie A (1978) Comparative clinical study of protein SAP (amyloid P component) and C-reactive protein in serum. *Clin Exp Immunol* 32: 119–124
Pepys MB, Hirschfield GM, Tennent GA, Gallimore JR, Kahan MC, Bellotti V, Hawkins PN, Myers RM, Smith MD, Polara A, *et al* (2006) Targeting C-reactive protein for the treatment of cardiovascular disease. *Nature* 440: 1217–1221
Pilishvili T, Lexau C, Farley MM, Hadler J, Harrison LH, Bennett NM, Reingold A, Thomas A, Schaffner W, Craig AS, *et al* (2010) Sustained Reductions in Invasive Pneumococcal Disease in the Era of Conjugate Vaccine. *J Infect Dis* 201: 32–41
Reifenberg K, Lehr H-A, Baskal D, Wiese E, Schaefer SC, Black S, Samols D, Torzewski M, Lackner KJ, Husmann M, *et al* (2005) Role of C-Reactive Protein in Atherogenesis: Can the Apolipoprotein E Knockout Mouse Provide the Answer? *Arterioscler Thromb Vasc Biol* 25: 1641–1646
Ries W, Heigl F, Garlichs C, Sheriff A & Torzewski J (2019) Selective C-Reactive Protein-Apheresis in Patients. *Ther Apher Dial* 23: 570–574
Ries W, Torzewski J, Heigl F, Pfluecke C, Kelle S, Darius H, Ince H, Mitzner S, Nordbeck P, Butter C, *et al* (2021) C-Reactive Protein Apheresis as Anti-inflammatory Therapy in Acute Myocardial Infarction: Results of the CAMI-1 Study. *Front Cardiovasc Med* 8: 591714
Ringel J, Ramlow A, Bock C & Sheriff A (2021) Case Report: C-Reactive Protein Apheresis in a Patient With COVID-19 and Fulminant CRP Increase. *Front Immunol* 12: 708101
Rossaint J, Margraf A & Zarbock A (2018) Role of Platelets in Leukocyte Recruitment and Resolution of Inflammation. *Front Immunol* 9: 2712
Schumann C, Heigl F, Rohrbach IJ, Sheriff A, Wagner L, Wagner F & Torzewski J (2021) A Report on the First 7 Sequential Patients Treated Within the C-Reactive Protein Apheresis in COVID (CACOV) Registry. *Am J Case Rep* 23
Schwedler SB, Guderian F, Dämmrich J, Potempa LA & Wanner C (2003) Tubular staining of modified C-reactive protein in diabetic chronic kidney disease. *Nephrol Dial Transplant Off Publ Eur Dial Transpl Assoc - Eur Ren Assoc* 18: 2300–2307
Sheriff A, Schindler R, Vogt B, Abdel-Aty H, Unger JK, Bock C, Gebauer F, Slagman A, Jerichow T, Mans D, *et al* (2015) Selective apheresis of C-reactive protein: A new therapeutic option in myocardial infarction?: Selective CRP-Apheresis. *J Clin Apheresis* 30: 15–21
Singh SK, Hammond DJ, Beeler BW & Agrawal A (2009) The binding of C-reactive protein, in the presence of phosphoethanolamine, to low-density lipoproteins is due to phosphoethanolamine-generated acidic pH. *Clin Chim Acta Int J Clin Chem* 409: 143–144
Taylor KE & van den Berg CW (2007) Structural and functional comparison of native pentameric, denatured monomeric and biotinylated C-reactive protein. *Immunology* 120: 404–411
Thiele JR, Habersberger J, Braig D, Schmidt Y, Goerendt K, Maurer V, Bannasch H, Scheichl A, Woollard KJ, von Dobschütz E, *et al* (2014) Dissociation of Pentameric to Monomeric C-Reactive Protein Localizes and Aggravates Inflammation: In Vivo Proof of a Powerful Proinflammatory Mechanism and a New Anti-Inflammatory Strategy. *Circulation* 130: 35–50
Thiele JR, Zeller J, Kiefer J, Braig D, Kreuzaler S, Lenz Y, Potempa LA, Grahammer F, Huber TB, Huber-Lang M, *et al* (2018) A Conformational Change in C-Reactive Protein Enhances Leukocyte Recruitment and Reactive Oxygen Species Generation in Ischemia/Reperfusion Injury. *Front Immunol* 9: 675
Torzewski J, Brunner P, Ries W, Garlichs CD, Kayser S, Heigl F & Sheriff A (2022) Targeting

C-Reactive Protein by Selective Apheresis in Humans: Pros and Cons. *J Clin Med* 11: 1771
Torzewski J, Heigl F, Zimmermann O, Wagner F, Schumann C, Hettich R, Bock C, Kayser S & Sheriff A (2020) First-in-Man: Case Report of Selective C-Reactive Protein Apheresis in a Patient with SARS-CoV-2 Infection. *Am J Case Rep* 21
Torzewski J, Zimmermann O, Kayser S, Heigl F, Wagner F, Sheriff A & Schumann C (2021) Successful Treatment of a 39-Year-Old COVID-19 Patient with Respiratory Failure by Selective C-Reactive Protein Apheresis. *Am J Case Rep* 22: e932964
Van Dijk WC, Verbrugh HA, van der Tol ME, Peters R & Verhoef J (1979) Role of Escherichia coli K capsular antigens during complement activation, C3 fixation, and opsonization. *Infect Immun* 25: 603–609
Wang MS, Messersmith RE & Reed SM (2012) Membrane curvature recognition by C-reactive protein using lipoprotein mimics. *Soft Matter* 8: 7909
Warren MS, Hughes SG, Singleton W, Yamashita M & Genovese MC (2015) Results of a proof of concept, double-blind, randomized trial of a second generation antisense oligonucleotide targeting high-sensitivity C-reactive protein (hs-CRP) in rheumatoid arthritis. *Arthritis Res Ther* 17: 80
Wiemer AJ (2020) Metabolic Efficacy of Phosphate Prodrugs and the Remdesivir Paradigm. *ACS Pharmacol Transl Sci* 3: 613–626
Yipp BG & Kubes P (2013) NETosis: how vital is it? *Blood* 122: 2784–2794
Zhang J, Yang L, Anand GS, Ho B & Ding JL (2011) Pathophysiological condition changes the conformation of a flexible FBG-related protein, switching it from pathogen-recognition to host-interaction. *Biochimie* 93: 1710–1719
Zhang L, Li H-Y, Li W, Shen Z-Y, Wang Y-D, Ji S-R & Wu Y (2018) An ELISA Assay for Quantifying Monomeric C-Reactive Protein in Plasma. *Front Immunol* 9: 511

6th Oct 2022

Dear Prof. Eisenhardt,

Thank you for the submission of your revised manuscript to EMBO Molecular Medicine, and please accept my apologies for the delay in getting back to you. We have now received the enclosed reports from the referees who re-reviewed your manuscript. As you will see, they are now supportive of publication pending minor revisions, and I am therefore pleased to inform you that we will be able to accept your manuscript once the following editorial points will be addressed:

1/ Referees' comments:
Please address the remaining concerns from the referees.

2/ Main manuscript text:
- Please remove the coloured text and accept the changes. Only keep in track changes mode any new modification.
- Material and methods:
o Please provide the antibodies concentrations.
o Human studies: please include the full statement that informed consent was obtained from all subjects and that the experiments conformed to the principles set out in the WMA Declaration of Helsinki and the Department of Health and Human Services Belmont Report.
- Data availability: Please note that the datasets must be publicly available before online publication.

3/ Thank you for providing Source Data for your figures. Please carefully check their labeling, especially for EV figures.

4/ Checklist:
Please modify the Data Availability section (datasets should not be provided as Source Data but deposited in public repository).

5/ I slightly modified your synopsis text, please let me know if you agree with the following or amend as you see fit:
A novel low molecular weight compound C10M was designed to block the PC binding site on pCRP, thereby inhibiting the formation of the pro-inflammatory isoforms pCRP*/mCRP, and thus showing broad anti-inflammatory effects in vitro and in vivo.
- The feasibility of the monovalent approach of PC binding site inhibition was demonstrated directly by X-ray crystallography.
- The pro-inflammatory conformational change of pCRP was blocked by a novel monovalent inhibitor utilizing the PC binding site, leaving the B-face otherwise accessible.
- The compound C10M inhibited pCRP*/mCRP-dependent pro-inflammatory effects on endothelial cells, monocytes and leukocytes in vitro.
- pCRP*/mCRP-driven inflammation in renal ischemia/reperfusion-injury and VCA hindlimb rejection in vivo was markedly inhibited by C10M.
Thank you for providing a nice visual abstract. Please upload it as an individual PNG/JPEG/TIFF file 550 px wide x 300-600 px high and make sure that the text remains legible.

6/ As part of the EMBO Publications transparent editorial process initiative (see our Editorial at http://embomolmed.embopress.org/content/2/9/329), EMBO Molecular Medicine will publish online a Review Process File (RPF) to accompany accepted manuscripts.
This file will be published in conjunction with your paper and will include the anonymous referee reports, your point-by-point response and all pertinent correspondence relating to the manuscript. Let us know whether you agree with the publication of the RPF and as here, if you want to remove or not any figures from it prior to publication.
Please note that the Authors checklist will be published at the end of the RPF.

I look forward to receiving your revised manuscript.

Yours sincerely,

Lise Roth

Lise Roth, PhD
Senior Editor
EMBO Molecular Medicine

*Additional important information regarding Figures

Each figure should be given in a separate file and should have the following resolution:
Graphs 800-1,200 DPI
Photos 400-800 DPI
Colour (only CMYK) 300-400 DPI"

Figures are not edited by the production team. All lettering should be the same size and style; figure panels should be indicated by capital letters (A, B, C etc). Gridlines are not allowed except for log plots. Figures should be numbered in the order of their appearance in the text with Arabic numerals. Each Figure must have a separate legend and a caption is needed for each panel.

*Additional important information regarding figures and illustrations can be found at https://bit.ly/EMBOPressFigurePreparationGuideline. See also figure legend preparation guidelines: https://www.embopress.org/page/journal/17574684/authorguide#figureformat

The system will prompt you to fill in your funding and payment information. This will allow Wiley to send you a quote for the article processing charge (APC) in case of acceptance. This quote takes into account any reduction or fee waivers that you may be eligible for. Authors do not need to pay any fees before their manuscript is accepted and transferred to our publisher.

***** Reviewer's comments *****

Referee #1 (Comments on Novelty/Model System for Author):

This manuscript contains an impressively comprehensive and well thought-out scientific work, which also gives it the scope of a qualification thesis. CRP has fulfilled Koch's postulate for a pathogen for over 20 years. Therefore, advances in therapies targeting CRP are desirable.

Referee #1 (Remarks for Author):

I cannot find Fig 4 H as outlined in your Point-by-Point response. If I just missed it, please explain the abbreviations: LP, PMA. The revision has not been fully edited, which makes reviewing difficult (e.g. 1399ff, 1525ff, 1698ff). It is also inconvenient that the references to the lines where changes are to be found are not correct at any point.

Referee #2 (Comments on Novelty/Model System for Author):

The revised manuscript provides more detailed in-depth functional characterization of C10M, a phosphocholine-mimicking compound that inhibit formation of pCRP*/mCRP. The conclusions are supported by the experimental data. The manuscript is of interest, but some minor issues may need further attention to increase the clarity of the presentation.

Referee #2 (Remarks for Author):

The authors have gone a long way to address my previous concerns. The new data lend additional support to the beneficial actions of the CRP conformational change blocker C10M. While the revision has further strengthened the manuscript, few minor issues may need further attention.

1. Fig. 3F. Please indicate the name of cytokine on each panel.
2. Fig. 4D. Molecular weight markers are missing.
3. Fig. 5F. Bands for pCRP in muscle and skin appear to have different molecular weight. Please clarify and also include molecular weight markers.
4. Fig. 6. Names of bacteria should be in italics. Since the Y-axis indicate bacteria/particle, repeating the same information in the panel title may be superfluous.

Referee #3 (Comments on Novelty/Model System for Author):

Excellent study with high potential to be translated

Referee #3 (Remarks for Author):

All concerns were satisfactorily addressed

Manuscript Number EMM-2022-16236-V2, “A novel phosphocholine-mimetic inhibits a pro-inflammatory conformational change of C-reactive protein”

Response to the Reviewer comments

We thank the Editor and the Referees for their very positive evaluation of our manuscript and the highly constructive comments. In the following, we provide a detailed point-by-point response to the Editor’s and Referees’ comments. We repeat the Referees’ comments in bold and our answer in indented paragraphs. Changes in the revised manuscript are highlighted in blue color.

Response to Referee 1:

Referee #1 (Comments on Novelty/Model System for Author):
This manuscript contains an impressively comprehensive and well thought-out scientific work, which also gives it the scope of a qualification thesis. CRP has fulfilled Koch's postulate for a pathogen for over 20 years. Therefore, advances in therapies targeting CRP are desirable.

> Answer:
> We again thank the Referee for their very positive, encouraging and constructive comments. In the following, we provide a detailed response to the Referee’s remarks and concerns.

Referee #1 (Remarks for Author):
I cannot find Fig 4 H as outlined in your Point-by-Point response. If I just missed it, please explain the abbreviations: LP, PMA.
The revision has not been fully edited, which makes reviewing difficult (e.g. 1399ff, 1525ff, 1698ff). It is also inconvenient that the references to the lines where changes are to be found are not correct at any point.

> Answer:
> We thank the Referee for bringing this to our attention. We apologize for any inconvenience or confusion that arose due to incorrect line references. It appears that an unnoticed frame shift occurred during the manuscript submission process, resulting in the error in line references. We apologize for not noticing this.

We are not sure whether we understand the Referee's comment in regard to Figure 4H. We show the revised Figure 4H in this Response to the Reviewers and have also ensured that the respective Figure in the manuscript is depicted correctly. We hope that the Referee's comments regarding the location of Figure 4H have now been addressed.

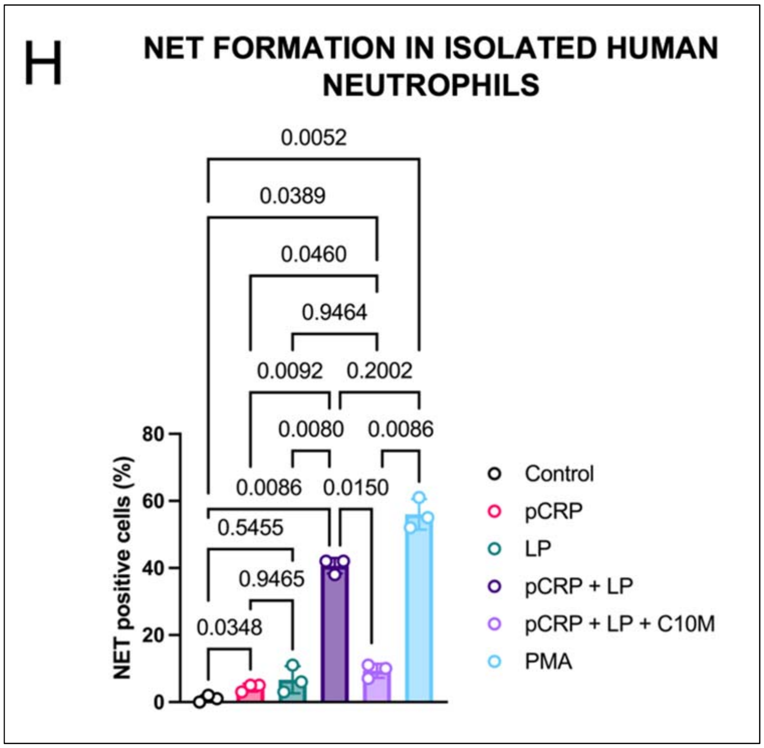

"Figure 4: pCRP binding to ADP-activated platelets is inhibited by C10M, reducing expression of adhesion molecules involved in leukocyte diapedesis in endothelial cells and leukocytes, ROS formation and NET formation.

H pCRP*/mCRP dependent NETosis in isolated human neutrophils detected by confocal immunofluorescence microscopy. Isolated neutrophils incubated for 3 hours at 37 °C, 5% CO_2 with 100 µg/ml pCRP with and without PC:LPC liposomes (LP) and C10M (molar ratio 1:100, pCRP:C10M), respectively. Control was left unstimulated and 100 nM phorbol 12-myristate 13-acetate (PMA) served as positive control. Cells were washed, fixed and stained, and analyzed by confocal microscopy. Results are given as a ratio of NETing cells/all cells per ROI, with mean ± SEM. *P* values were calculated with ANOVA and Tukey's post-hoc test. Biological replicates, n=3."

We now added an explanation for the two abbreviations, "LP" for liposomes and "PMA" for phorbol 12-myristate 13-acetate in the appropriate figure legends (please find above) and in the Methods section (line 1116ff):

"Cells were stimulated with pCRP (100 µg/ml), with or without PC:LPC liposomes (LP; 20 µg/ml) and C10M (1:100 molar ratio, pCRP:C10M) for 3 hours at 37°C, 5 %CO_2. Phorbol 12-myristate 13-acetate (PMA; 100 nM) served as a positive control. The cell activity was stopped by adding 4% PFA, as PFA at concentrations of 1–4% fix but do not permeate the plasma membrane (Masuda et al., 2016)."

Please find our newly submitted version of our manuscript without track-changes and high-lighted wording, except for newly added changes needed to answer the remaining Referees' questions/remarks, as requested by the handling editor.

Response to Referee 2:

Referee #2 (Comments on Novelty/Model System for Author):

The revised manuscript provides more detailed in-depth functional characterization of C10M, a phosphocholine-mimicking compound that inhibit formation of pCRP*/mCRP. The conclusions are supported by the experimental data. The manuscript is of interest, but some minor issues may need further attention to increase the clarity of the presentation.

Answer:
We would like to thank the Referee for the very positive review and insightful comments. We appreciate the highly constructive suggestions. Please find detailed answers to the Referee's remaining remarks below.

Referee #2 (Remarks for Author):

The authors have gone a long way to address my previous concerns. The new data lend additional support to the beneficial actions of the CRP conformational change blocker C10M. While the revision has further strengthened the manuscript, few minor issues may need further attention.

Answer:
We thank the Referee for appreciating our work and for the help they provided to improve this manuscript. We have now addressed the remaining concerns.

Remark 1: Fig. 3F. Please indicate the name of cytokine on each panel.

Answer:
We thank the Referee for highlighting this omission, the figure panel in question is 3E. We have now added titles to each graph indicating the specific cytokine detected (please see below).

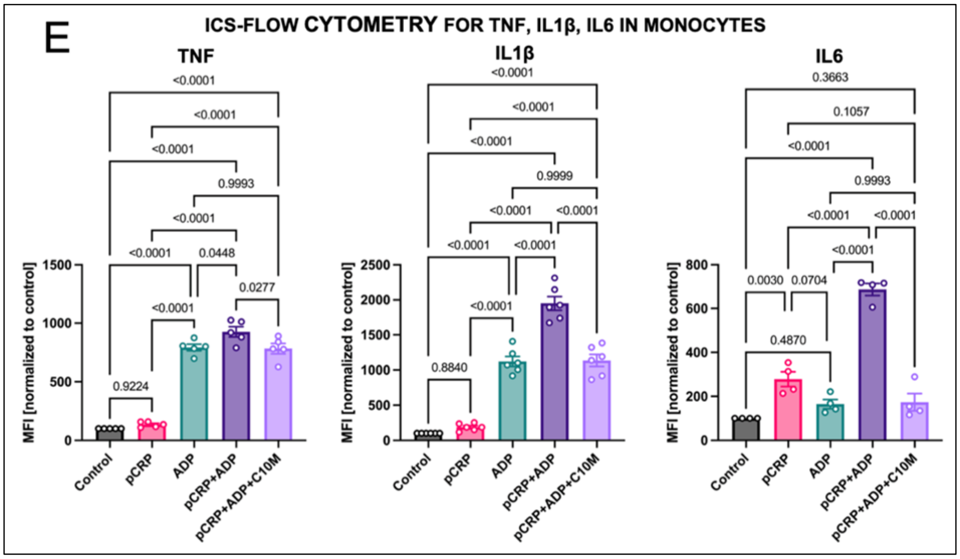

“**Figure 3: Studies on CRP-dependent expression of pro-inflammatory cytokines and inhibiting effects of C10M.**

E Expression of TNF, IL1β and IL6 (from left to right) in monocytes analyzed by flow cytometry. Addition of C10M to the whole blood samples inhibits the CRP-dependent expression of pro-inflammatory cytokines. P values were calculated with ANOVA and *Tukey’s* post-hoc test. Biological replicates, n=6 (IL-6), n=5 (TNF) and n=4 (IL-1β), respectively, bars indicate mean ± SEM.”

Remark 2: Fig. 4D. Molecular weight markers are missing.

Answer:

We thank the Referee for pointing this out. We presume the Referee is referring to the Western blot in Figure 5. We have now added an arrow to indicate what height the molecular weight runs on the SDS-PAGE shown in Figure 5D. We further provide the uncropped images of the Western blots.

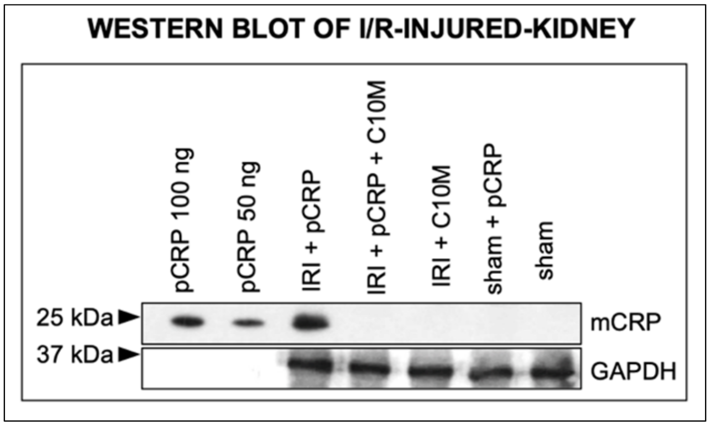

"Figure 5: pCRP*/mCRP-driven exacerbation of renal ischemia/reperfusion injury is reduced by C10M.

D Tissue lysates of rat kidney were separated on SDS-PAGE and total CRP was identified with anti-CRP antibody. A band at the size of mCRP (~23 kDa) was detected in kidneys subjected to IRI and pCRP, but not in animals treated additionally with C10M. The household gene protein GAPDH served as a control for loading equal amounts of protein. 50 and 100 ng human pCRP, respectively, served as a positive control. Representative results are shown for replicated assays (n=3)."

Remark 3: Fig. 5F. Bands for pCRP in muscle and skin appear to have different molecular weight. Please clarify and also include molecular weight markers.

Answer:

We thank the Referee for their remark. We presume the Referee is referring to the Western blot in Figure 6F showing data of the hind limb transplantation model. The different molecular weight might be due to different redox states of the tissue-bound mCRP as demonstrated in a previous study (Wang et al., 2011). In this paper, the authors reported that the redox state affects the relative size of the protein in SDS-PAGE. The rejected hind limb tissue (muscle and skin) is in itself challenging in preparation for Western blotting, especially compared to single cell suspensions or parenchymal tissue such as kidney. However, at the size of the CRP control (approximately 25 kDa), bands were only observed in the transplanted hindlimb treated with pCRP, in contrast the pCRP+C10M group showed only a faint signal. We have now added arrows to indicate the molecular weight and added the following sentence to the main text (line 377):

"Fig 6F; here, the CRP detection was found to run at slightly different sizes, which potentially reflects different redox states of the deposited protein (Wang et al., 2011)"

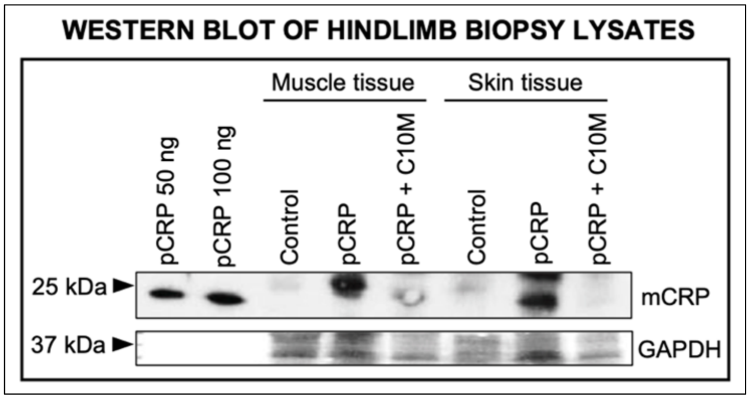

"Figure 6: Compound C10M delays CRP-driven transplant rejection in a hindlimb transplantation model.

F Exemplary tissue lysates of muscle and skin probes separated on SDS-PAGE are depicted. Total human CRP was identified with an anti-CRP antibody."

Remark 4: Fig. 6. Names of bacteria should be in italics. Since the Y-axis indicate bacteria/particle, repeating the same information in the panel title may be superfluous.

Directly following the Referee's recommendation for Figure 7, we amended the labeling of the graphs and the titles (please find the figure added below). We now provide the bacterial names in the title in italics and we removed the superfluous labeling from the y-axis. Thus, the labeling of the y-axis is no longer redundant and we were able to increase the font size for better readability. We changed EV Figure 3 accordingly (please find below). We thank the Referee for this constructive remark.

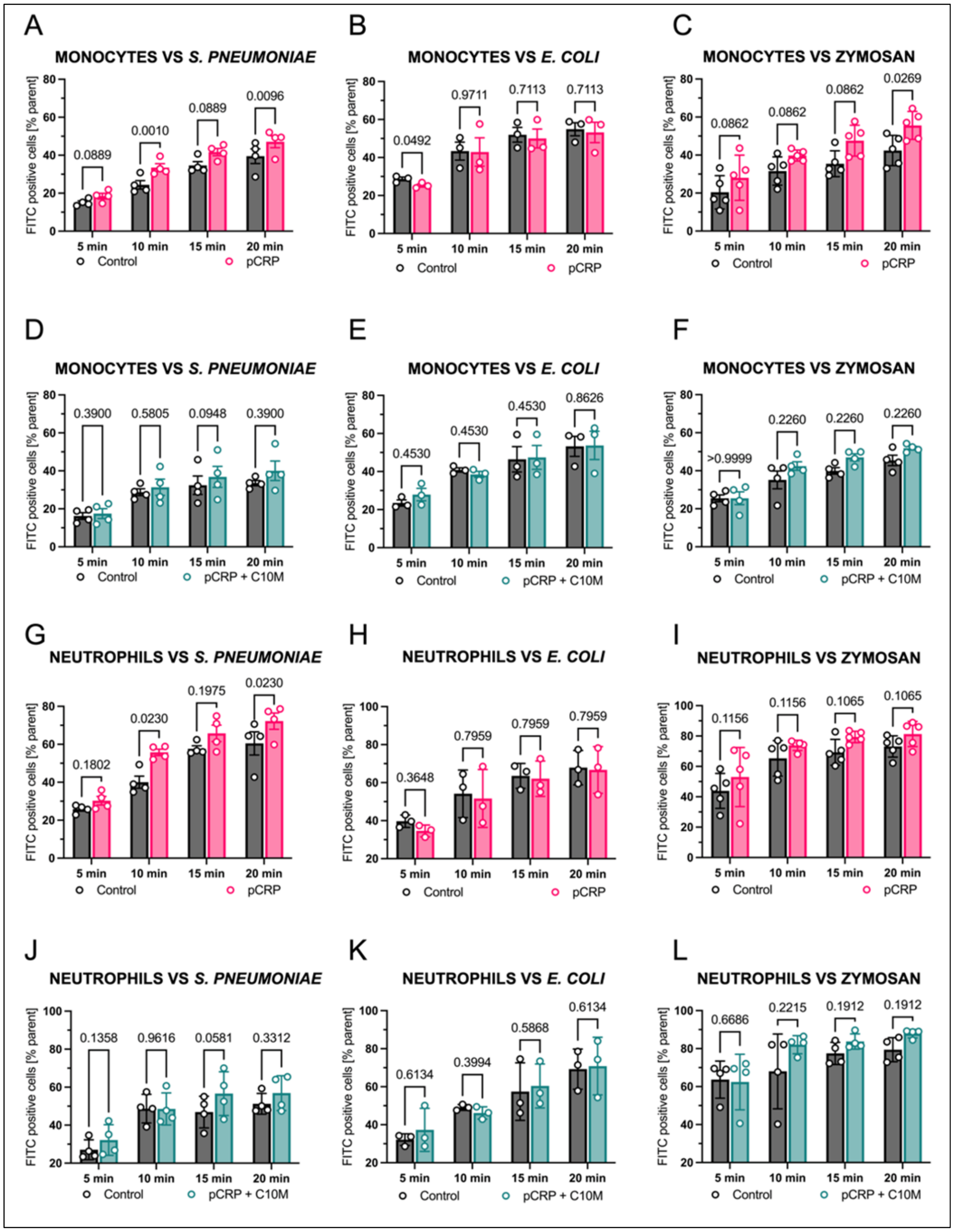

“Figure 7: Flow cytometry-based analysis of CRP-dependent opsonophagocytosis of *S. pneumoniae*, *E. coli*, and zymosan in the presence of C10M.”

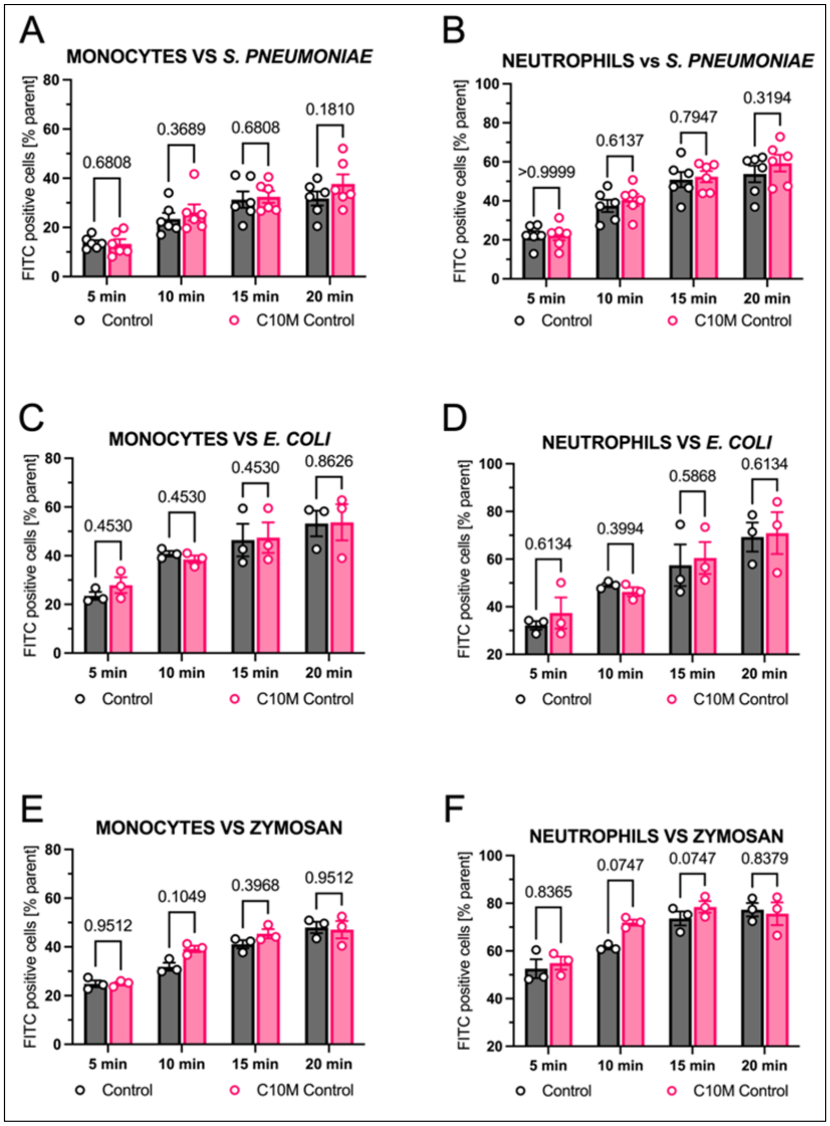

"Figure EV3: Flow cytometry-based analysis of phagocytosis of *S. pneumoniae*, *E. coli*, and zymosan in the presence and the absence of C10M."

Response to Referee 3:

Referee #3 (Comments on Novelty/Model System for Author):

Excellent study with high potential to be translated

Referee #3 (Remarks for Author):

All concerns were satisfactorily addressed

Answer:
We would like to thank the Referee for their highly positive feedback and their previous constructive comments, which clearly helped us to improve the revised manuscript.

References:

Masuda, S., Nakazawa, D., Shida, H., Miyoshi, A., Kusunoki, Y., Tomaru, U., & Ishizu, A. (2016). NETosis markers: Quest for specific, objective, and quantitative markers. *Clinica Chimica Acta; International Journal of Clinical Chemistry*, *459*, 89–93. https://doi.org/10.1016/j.cca.2016.05.029

Wang, M.-Y., Ji, S.-R., Bai, C.-J., El Kebir, D., Li, H.-Y., Shi, J.-M., Zhu, W., Costantino, S., Zhou, H.-H., Potempa, L. A., Zhao, J., Filep, J. G., & Wu, Y. (2011). A redox switch in C-reactive protein modulates activation of endothelial cells. *The FASEB Journal*, *25*(9), Article 9. https://doi.org/10.1096/fj.11-182741

6th Nov 2022

Dear Prof. Eisenhardt,

Thank you for submitting the revised files. I am pleased to inform you that your manuscript is accepted for publication and is now being sent to our publisher to be included in the next available issue of EMBO Molecular Medicine.

Please read below for additional IMPORTANT information regarding your article, its publication and the production process.

Congratulations on your interesting work!

With kind regards,

Lise Roth

Lise Roth, Ph.D
Senior Editor
EMBO Molecular Medicine

Follow us on Twitter @EmboMolMed
Sign up for eTOCs at embopress.org/alertsfeeds

*** *** *** IMPORTANT INFORMATION *** *** ***

SPEED OF PUBLICATION
The journal aims for rapid publication of papers, using using the advance online publication "Early View" to expedite the process: A properly copy-edited and formatted version will be published as "Early View" after the proofs have been corrected. Please help the Editors and publisher avoid delays by providing e-mail address(es), telephone and fax numbers at which author(s) can be contacted.

Should you be planning a Press Release on your article, please get in contact with embomolmed@wiley.com as early as possible, in order to coordinate publication and release dates.

LICENSE AND PAYMENT:

All articles published in EMBO Molecular Medicine are fully open access: immediately and freely available to read, download and share.

EMBO Molecular Medicine charges an article processing charge (APC) to cover the publication costs. You, as the corresponding author for this manuscript, should have already received a quote with the article processing fee separately. Please let us know in case this quote has not been received.

Once your article is at Wiley for editorial production you will receive an email from Wiley's Author Services system, which will ask you to log in and will present you with the publication license form for completion. Within the same system the publication fee can be paid by credit card, an invoice, pro forma invoice or purchase order can be requested.

Payment of the publication charge and the signed Open Access Agreement form must be received before the article can be published online.

PROOFS

You will receive the proofs by e-mail approximately 2 weeks after all relevant files have been sent o our Production Office. Please return them within 48 hours and if there should be any problems, please contact the production office at embopressproduction@wiley.com.

Please inform us if there is likely to be any difficulty in reaching you at the above address at that time. Failure to meet our deadlines may result in a delay of publication.

All further communications concerning your paper proofs should quote reference number EMM-2022-16236-V3 and be directed to the production office at embopressproduction@wiley.com.

EMBO Press Author Checklist

Corresponding Author Name: Prof. Steffen Ulrich Eisenhardt	
Journal Submitted to: EMBO Molecular Medicine	
Manuscript Number: # EMM-2022-16236	

USEFUL LINKS FOR COMPLETING THIS FORM
The EMBO Journal - Author Guidelines
EMBO Reports - Author Guidelines
Molecular Systems Biology - Author Guidelines
EMBO Molecular Medicine - Author Guidelines

Reporting Checklist for Life Science Articles (updated January 2022)
This checklist is adapted from Materials Design Analysis Reporting (MDAR) Checklist for Authors. MDAR establishes a minimum set of requirements in transparent reporting in the life sciences (see Statement of Task: 10.31222/osf.io/9sm4x). Please follow the journal's guidelines in preparing your manuscript.
Please note that a copy of this checklist will be published alongside your article.

Abridged guidelines for figures
1. Data
The data shown in figures should satisfy the following conditions:
- the data were obtained and processed according to the field's best practice and are presented to reflect the results of the experiments in an accurate and unbiased manner.
- ideally, figure panels should include only measurements that are directly comparable to each other and obtained with the same assay.
- plots include clearly labeled error bars for independent experiments and sample sizes. Unless justified, error bars should not be shown for technical replicates.
- if n<5, the individual data points from each experiment should be plotted. Any statistical test employed should be justified.
- Source Data should be included to report the data underlying figures according to the guidelines set out in the authorship guidelines on Data Presentation.

2. Captions
Each figure caption should contain the following information, for each panel where they are relevant:
- a specification of the experimental system investigated (eg cell line, species name).
- the assay(s) and method(s) used to carry out the reported observations and measurements.
- an explicit mention of the biological and chemical entity(ies) that are being measured.
- an explicit mention of the biological and chemical entity(ies) that are altered/varied/perturbed in a controlled manner.
- the exact sample size (n) for each experimental group/condition, given as a number, not a range;
- a description of the sample collection allowing the reader to understand whether the samples represent technical or biological replicates (including how many animals, litters, cultures, etc.).
- a statement of how many times the experiment shown was independently replicated in the laboratory.
- definitions of statistical methods and measures:
  - common tests, such as t-test (please specify whether paired vs. unpaired), simple χ2 tests, Wilcoxon and Mann-Whitney tests, can be unambiguously identified by name only, but more complex techniques should be described in the methods section;
  - are tests one-sided or two-sided?
  - are there adjustments for multiple comparisons?
  - exact statistical test results, e.g., P values = x but not P values < x;
  - definition of 'center values' as median or average;
  - definition of error bars as s.d. or s.e.m.

Please complete ALL of the questions below.
Select "Not Applicable" only when the requested information is not relevant for your study.

Materials

Newly Created Materials	Information included in the manuscript?	In which section is the information available? (Reagents and Tools Table, Materials and Methods, Figures, Data Availability Section)
New materials and reagents need to be available; do any restrictions apply?	Yes	Materials and Methods, Synthesis of compound C10M; Conflict of interest statement

Antibodies	Information included in the manuscript?	In which section is the information available? (Reagents and Tools Table, Materials and Methods, Figures, Data Availability Section)
For **antibodies** provide the following information:<br>- Commercial antibodies: RRID (if possible) or supplier name, catalogue number and or/clone number<br>- Non-commercial: RRID or citation	Yes	Reagent and Tools Table; respective assay in Materials and Methods

DNA and RNA sequences	Information included in the manuscript?	In which section is the information available? (Reagents and Tools Table, Materials and Methods, Figures, Data Availability Section)
**Short novel DNA or RNA including primers, probes**: provide the sequences.	Not Applicable	

Cell materials	Information included in the manuscript?	In which section is the information available? (Reagents and Tools Table, Materials and Methods, Figures, Data Availability Section)
**Cell lines:** Provide species information, strain. Provide accession number in repository **OR** supplier name, catalog number, clone number, and/**OR** RRID.	Yes	Reagent and Tool Table in "Material and Methods" section
**Primary cultures:** Provide species, strain, sex of origin, genetic modification status.	Yes	"Human ex vivo studies" in "Material and Methods" section
Report if the cell lines were recently **authenticated** (e.g., by STR profiling) and tested for mycoplasma contamination.	Yes	For THP-1 cell line in Appendix

Experimental animals	Information included in the manuscript?	In which section is the information available? (Reagents and Tools Table, Materials and Methods, Figures, Data Availability Section)
**Laboratory animals or Model organisms:** Provide species, strain, sex, age, genetic modification status. Provide accession number in repository **OR** supplier name, catalog number, clone number, **OR** RRID.	Yes	Species: Rattus norvegicus; Strain: Brown-Norway, Lewis, Wistar; Age: six-weeks old; Genetic modifications: none; Supplier: Charles River Research Models and Services, Sulzfeld, Germany; "ARRIVE guideline information" in Appendix
**Animal observed in or captured from the field:** Provide species, sex, and age where possible.	Not Applicable	
Please detail **housing and husbandry conditions**.	Yes	Animals were cared for by the professional animal keepers of the Center for Experimental Models and Transgenic Service (CEMT), University of Freiburg

Plants and microbes	Information included in the manuscript?	In which section is the information available? (Reagents and Tools Table, Materials and Methods, Figures, Data Availability Section)
**Plants:** provide species and strain, ecotype and cultivar where relevant, unique accession number if available, and source (including location for collected wild specimens).	Not Applicable	
**Microbes:** provide species and strain, unique accession number if available, and source.	Not Applicable	

Human research participants	Information included in the manuscript?	In which section is the information available? (Reagents and Tools Table, Materials and Methods, Figures, Data Availability Section)
If collected and within the bounds of privacy constraints report on age, sex and gender or ethnicity for all study participants.	Yes	Blood donors: Age: 21 - 36 years; Sex: male and female; Ethnicity: caucasian; under "Human ex vivo studies" in Material and Methods

Core facilities	Information included in the manuscript?	In which section is the information available? (Reagents and Tools Table, Materials and Methods, Figures, Data Availability Section)
If your work benefited from core facilities, was their service mentioned in the acknowledgments section?	Not Applicable	

Design

**Study protocol**	**Information included in the manuscript?**	**In which section is the information available?** (Reagents and Tools Table, Materials and Methods, Figures, Data Availability Section)
If study protocol has been **pre-registered, provide DOI in the manuscript**. For clinical trials, provide the trial registration number **OR** cite DOI.	Not Applicable	
Report the **clinical trial registration number** (at ClinicalTrials.gov or equivalent), where applicable.	Not Applicable	

**Laboratory protocol**	**Information included in the manuscript?**	**In which section is the information available?** (Reagents and Tools Table, Materials and Methods, Figures, Data Availability Section)
Provide DOI OR other citation details if **external detailed step-by-step protocols** are available.	Yes	Kiefer J, Zeller J, Bogner B, Hörbrand IA, Lang F, Deiss E, Winninger O, Fricke M, Kreuzaler S, Smudde E, Huber-Lang M, Peter K, Woollard KJ and Eisenhardt SU (2021) An Unbiased Flow Cytometry-Based Approach to [illegible]

**Experimental study design and statistics**	**Information included in the manuscript?**	**In which section is the information available?** (Reagents and Tools Table, Materials and Methods, Figures, Data Availability Section)
Include a statement about **sample size** estimate even if no statistical methods were used.	Yes	Appendix, ARRIVE guideline information
Were any steps taken to minimize the effects of subjective bias when allocating animals/samples to treatment (e.g. **randomization procedure**)? If yes, have they been described?	Yes	Appendix, ARRIVE guideline information
Include a statement about **blinding** even if no blinding was done.	Yes	Appendix, ARRIVE guideline information
Describe **inclusion/exclusion criteria** if samples or animals were excluded from the analysis. Were the criteria pre-established?<br><br>If sample or data points were omitted from analysis, report if this was due to attrition or intentional exclusion and provide justification.	Yes	Appendix, ARRIVE guideline information
For every figure, are **statistical tests** justified as appropriate? Do the data meet the assumptions of the tests (e.g., normal distribution)? Describe any methods used to assess it. Is there an estimate of variation within each group of data? Is the variance similar between the groups that are being statistically compared?	Yes	Employed statistics are given in detail in Source data file and in figure legends.

**Sample definition and in-laboratory replication**	**Information included in the manuscript?**	**In which section is the information available?** (Reagents and Tools Table, Materials and Methods, Figures, Data Availability Section)
In the figure legends: state number of times the experiment was **replicated** in laboratory.	Yes	The number of replicates/animals/sample size is given in each figure legend.
In the figure legends: define whether data describe **technical or biological replicates**.	Yes	Information is given in the figure legends.

Ethics

**Ethics**	**Information included in the manuscript?**	**In which section is the information available?** (Reagents and Tools Table, Materials and Methods, Figures, Data Availability Section)
Studies involving **human participants**: State details of **authority granting ethics approval** (IRB or equivalent committee(s), provide reference number for approval.	Yes	Material and Methods, "Human ex vivo studies"
Studies involving **human participants**: Include a statement confirming that **informed consent** was obtained from all subjects and that the experiments conformed to the principles set out in the WMA Declaration of Helsinki and the Department of Health and Human Services Belmont Report.	Yes	Material and Methods, "Human ex vivo studies"
Studies involving **human participants:** For publication of **patient photos**, include a statement confirming that consent to publish was obtained.	Not Applicable	
Studies involving experimental **animals**: State details of **authority granting ethics approva**l (IRB or equivalent committee(s), provide reference number for approval. Include a statement of compliance with ethical regulations.	Yes	Material and Methods, "Animal ethics and experiments"; "ARRIVE guideline information" in Appendix
Studies involving **specimen and field samples:** State if relevant **permits** obtained, provide details of authority approving study; if none were required, explain why.	Not Applicable	

**Dual Use Research of Concern (DURC)**	**Information included in the manuscript?**	**In which section is the information available?** (Reagents and Tools Table, Materials and Methods, Figures, Data Availability Section)
Could your study fall under dual use research restrictions? Please check biosecurity documents and list of **select agents and toxins** (CDC): https://www.selectagents.gov/sat/list.htm	Not Applicable	
If you used a select agent, is the security level of the lab appropriate and reported in the manuscript?	Not Applicable	
If a study is subject to dual use research of concern regulations, is the name of the **authority granting approval and reference number** for the regulatory approval provided in the manuscript?	Not Applicable	

Reporting

The MDAR framework recommends adoption of discipline-specific guidelines, established and endorsed through community initiatives. Journals have their own policy about requiring specific guidelines and recommendations to complement MDAR.

**Adherence to community standards**	**Information included in the manuscript?**	**In which section is the information available?** (Reagents and Tools Table, Materials and Methods, Figures, Data Availability Section)
State if relevant guidelines or checklists (e.g., **ICMJE, MIBBI, ARRIVE, PRISMA**) have been followed or provided.	Yes	ARRIVE guidelines have been followed and information is given in Appendix
For **tumor marker prognostic studies**, we recommend that you follow the **REMARK** reporting guidelines (see link list at top right). See author guidelines, under 'Reporting Guidelines'. Please confirm you have followed these guidelines.	Not Applicable	
For **phase II and III randomized controlled trials**, please refer to the **CONSORT** flow diagram (see link list at top right) and submit the CONSORT checklist (see link list at top right) with your submission. See author guidelines, under 'Reporting Guidelines'. Please confirm you have submitted this list.	Not Applicable	

Data Availability

**Data availability**	**Information included in the manuscript?**	**In which section is the information available?** (Reagents and Tools Table, Materials and Methods, Figures, Data Availability Section)
Have **primary datasets** been deposited according to the journal's guidelines (see 'Data Deposition' section) and the respective accession numbers provided in the Data Availability Section?	Yes	The coordinates of compound C10M:pCRP complex have been deposited under PDB ID: 7TBA (https://www.rcsb.org/structure/7TBA). This study includes no additional data deposited in external repositories.
Were **human clinical and genomic datasets** deposited in a public access-controlled repository in accordance to ethical obligations to the patients and to the applicable consent agreement?	Not Applicable	
Are **computational models** that are central and integral to a study available without restrictions in a machine-readable form? Were the relevant accession numbers or links provided?	Not Applicable	
If publicly available data were reused, provide the respective **data citations in the reference list**.	Not Applicable	
